# Function Meets Circularity: Metal–Ionomer Cross-Links Toughen and Recycle CO_2_‑Derived Polymers

**DOI:** 10.1021/acs.macromol.6c00188

**Published:** 2026-03-12

**Authors:** Kam C. Poon, Thomas M. McGuire, Chang Gao, Gregory S. Sulley, Charlotte K. Williams

**Affiliations:** Chemistry Research Laboratory, Department of Chemistry, 6396 University of Oxford, Oxford OX1 3TA, U.K.

## Abstract

Designing polymers that combine performance with sustainability remains a critical challenge. Here, we report high-performance elastomers derived from CO_2_ and biobased monomers that integrate both mechanical toughness and closed-loop chemical recyclability through a single material feature: dynamic metal–ionomer cross-links. These ABA block polymers, synthesized from ε-decalactone, δ-jasmolactone, CO_2_, and bicyclic epoxides, incorporate abundant and inexpensive metal carboxylates (Na­(I), Zn­(II), and Al­(III)) into the midblock, forming reversible networks that enhance tensile strength by 150% while maintaining high strain at break (>1500%) and elastic recovery (>85%). The same cross-links act as built-in catalysts, enabling energy-efficient depolymerization of both polyester and polycarbonate domains at 200 °C, recovering the original monomers. This dual-function approach advances circular polymer design by combining enhanced performance with efficient, low-energy, closed-loop recycling.

## Graphical abstract

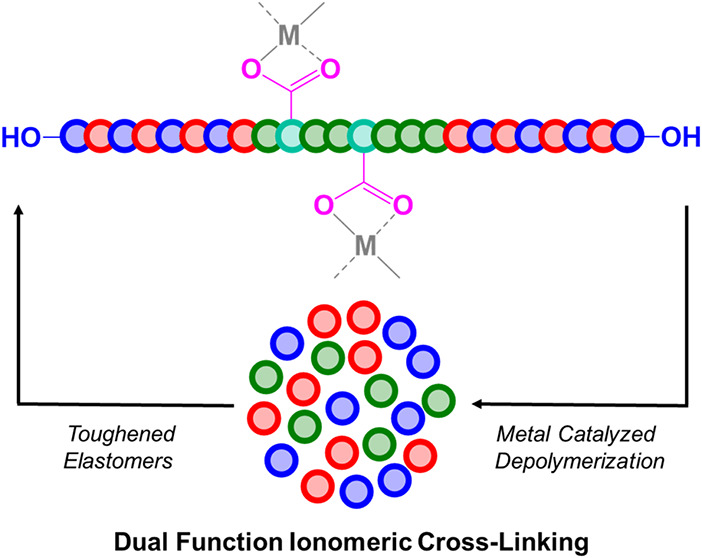

## Introduction

Polymers are deeply woven into the fabric of modern life, shaping everything from the materials we wear to the technologies we rely on. Plastic production now exceeds 460 megatonnes a year, but only 9% of global plastic waste is recycled into new products.^1^ Most of the plastic produced either ends up in landfill, terrestrial and marine ecosystems, or is incinerated.^2^ This plastic consumption results in the emission of more than 1.8 gigatonnes of CO_2_-equivalents annually which must be reduced if global warming is to be kept below 1.5 °C. This can only be achieved through the use of sustainable feedstocks and effective, widespread closed-loop recycling.^3,4^ Even under the most ambitious forecasts for the future plastics economy, with the elimination of petrochemical feedstocks and drastically improved recycling, there will remain the need for the production of new biobased materials.^5^

One of the most pressing frontiers in polymer science is the creation of chemistries that unite high-performance properties with chemical circularity.^6,7^ Until now, advances in thermomechanical properties have too often undermined recyclability, and *vice versa*.^8,9^ The future of sustainable polymers depends on breaking this trade-off, designing materials that deliver uncompromising performance while enabling full chemical recovery and reuse.

To simultaneously meet property demands for polymeric materials while remaining on course to meet waste targets, fully recyclable polymers produced from carbon dioxide (CO_2_) and biomass are important alternatives to incumbent petroleum-derived materials.^10^ Moreover, it is essential that new polymers are designed holistically to ensure there are multiple viable end-of-life options.^11^ Utilizing CO_2_ as a monomer in the ring-opening copolymerization (ROCOP) with epoxides is particularly appealing as it not only reduces pollution in polymer manufacturing but valorizes the cheap and overly abundant feedstock.^12–14^ Furthermore, the utilization of CO_2_ to produce polycarbonates via ROCOP results in a 3-fold reduction in greenhouse gas emissions relative to the corresponding polyether: two molecules of CO_2_ are saved through the replacement of the epoxide in addition to the one CO_2_ molecules incorporated into every repeat unit.^15–17^

Recent developments in epoxide/heteroallene ROCOP have yielded well-defined oxygenated block polymers with potential applications as engineering plastics and elastomers.^18^ These ABA block copolymers are composed of a “soft” (glass transition temperature (*T*_g_) < room temperature) central block flanked either side by glassy (*T*_g_ > room temperature) outer blocks.^19^ Wu and co-workers have reported all polycarbonate ABA block polymer elastomers composed of poly­(cyclohexene carbonate) A blocks and poly­(allyl glycidyl ether carbonate) B blocks. Later, Feng and co-workers demonstrated that the B block could be replaced with poly­(octene carbonate) to also yield moderately strong elastomers.^20,21^ Our group has reported the use of switch catalysis, coupling lactone ring-opening polymerization (ROP) with epoxide/CO_2_ ROCOP to produce poly­(carbonate-*b*-ester-*b*-carbonate) materials. Sulley et al. reported a series of triblock copolymers also with poly­(cyclohexene carbonate) A blocks but with a poly­(decalactone) midblock.^19^ By tuning the polycarbonate content pressure sensitive adhesives, elastomers, and engineering plastics were produced.

However, the thermomechanical performance of these materials, in particular the ultimate tensile strength and strain at break, often falls short of those required to replace current petrochemical elastomers. Postpolymerization functionalization strategies including hydrogen bonding, stereocomplexation, and dynamic covalent cross-links have all been leveraged to improve the mechanical properties of soft polymers.^7^ One particularly attractive approach is to employ a small amount of biomimetic metal–ligand interactions to produce ionomers.^22^ These reversible cross-links allow improved and tunable bulk mechanical properties through variation of both the metal and ligand employed.^23–25^ This strategy is employed in the commercial food packaging material Surlyn, a random copolymer of ethylene and methacrylic acid cross-linked with sodium ions, however the poorly defined polymer architecture, makes it difficult to isolate and study the specific effects of metal coordination chemistry on polymer properties.

Ionomeric interactions are well-known to improve the tensile mechanical properties of gels, where iron-catechol,^22^ sodium-carboxylate,^26^ zinc-carboxylate,^27^ iron-pyridyl,^28^ and zinc imidazole bonds are all reversible cross-links.^29^ Johnson and co-workers exploited such metal–ligand interactions to form cages within gels as a means to both tune material properties but and transform the gels into heterogeneous catalysts.^7,30,31^ These findings highlight the potential of metal–ligand cross-linking to not only improve mechanical properties but install further chemical functionality into a material.^31^ There remains a relatively small body of literature exploring ionomeric cross-links in elastomers and plastics.^32,33^

With ultimate tensile strengths and stiffnesses multiple orders of magnitude greater than that of gels, the potential improvements in mechanical performance with the introduction of metal–ligand cross-linking in elastomers and plastics is an attractive option. We have recently incorporated reversible metal–ligand ionomeric cross-links into the glassy polyester and polycarbonate blocks of oxygenated ABA polymers, strengthening elastomers while preserving thermal reprocessability and recyclability.^32–34^ Although mechanical recyclability of these materials was established, chemical recycling of these elastomeric networks was challenging. In prior CO_2_-derived systems, introducing ionomeric cross-links into the rigid polycarbonate domains reduced the strain at break, likely due to restricted segmental mobility and hindered dynamic exchange within the glassy phase. Only a handful of studies have explored the introduction of reversible and dynamic ionomeric cross-linking in the low *T*_g_ central block, yet result in materials which are both strong and tough.^35–37^

In addition to the use of renewable monomer feedstocks and competitive mechanical properties, future polymers should be designed for closed loop-recycling.^5^ Materials should be able to undergo both mechanical recycling (thermal reprocessing into new products) and chemical recycling to monomer (CRM).^6^ Chen and co-workers developed various monomer/polymer systems derived from γ-butyrolatones,^38,39^ δ-valerolactones,^40–42^ and polyhydroxyalkanoates,^43,44^ which are fully chemically recycled. These novel polyester plastics offer competitive material properties and are efficiently recycled to their constituent monomers. Coates and co-workers reported the chemical recycling and upcycling of polyhydroxybutyrates produced from 2-butene and carbon monoxide.^45^ Finally, Cai, Zhu, and co-workers report the CRM of substituted ε-caprolactones.^46^

Darensbourg and co-workers reported the first CO_2_-derived polycarbonate chemical recycling, transforming poly­(cyclopentene carbonate) to CO_2_ and cyclopentene oxide using a chromium salen catalyst.^47,48^ Recently, we reported the solid state depolymerization of a range of high molar mass homopolycarbonates and terpolymers to CO_2_ and the respective epoxides using high activity heterodinuclear catalysts.^49–51^ Subsequently, the same solid-state method enabled the successful depolymerization of poly­(lactones) to the constituent lactones using simple metal-carboxylate catalysts.^52,53^ In all these chemical recycling examples, the addition of a catalyst is required and/or temperatures above the ceiling temperature are used to drive the formation of monomers.^54,55^ The intentional incorporation of depolymerization catalysts into polymers would simplify chemical recycling, requiring only heating to trigger depolymerization, and represents an unexplored strategy for creating materials that simultaneously exhibit enhanced properties and controlled, catalyzed chemical recyclability.

Here, we report a new class of elastomers that resolve the long-standing tension between enhanced mechanical performance and chemical recyclability. These materials are based on poly­(carbonate-*b*-ester-*b*-carbonate) block polymers in which the central “soft” block is functionalized with carboxylate ligands via the copolymerization of bioderived ε-decalactone and δ-jasmolactone. Incorporation of metal–ligand cross-links into this low *T*_g_ block generates a reversible and dynamic network, in which the metal centers simultaneously toughen the material and serve as embedded depolymerization catalysts. In this way, mechanical robustness and chemical circularity are no longer competing objectives but are integrated within a single molecular design.

## Ionomer Synthesis

The initial, unfunctionalized, ABA triblock polymer was prepared utilizing a heterodinuclear [Zn­(II)­Mg­(II)] catalyst, capable of controlled and switchable polymerizations in a one-pot process. The catalyst was combined with the initiator 1,4-benzenedimethanol (BDM) together with the ε-decalactone (ε-DL), δ-jasmolactone (δ-JL), cyclohexene oxide (CHO), and cyclopentene oxide (CPO) monomers (Figure 1). The ROP of both ε-DL and δ-JL occurred first to form the hydroxy telechelic copolymer poly­(ε-decalactone-*co*-δ-jasmolactone) (PDL-*co*-PJL) as the central B block, with 10 mol % PJL content targeted. Meyer-Lowry analysis of the lactone polymerization determined the reactivity ratios of ε-DL and δ-JL to be 0.803 and 0.800, respectively (Figures S1–S2). Therefore, the resulting block possessed a random/statistical structure, composed mostly of PDL with PJL repeat units evenly enchained throughout.

**Figure 1:**
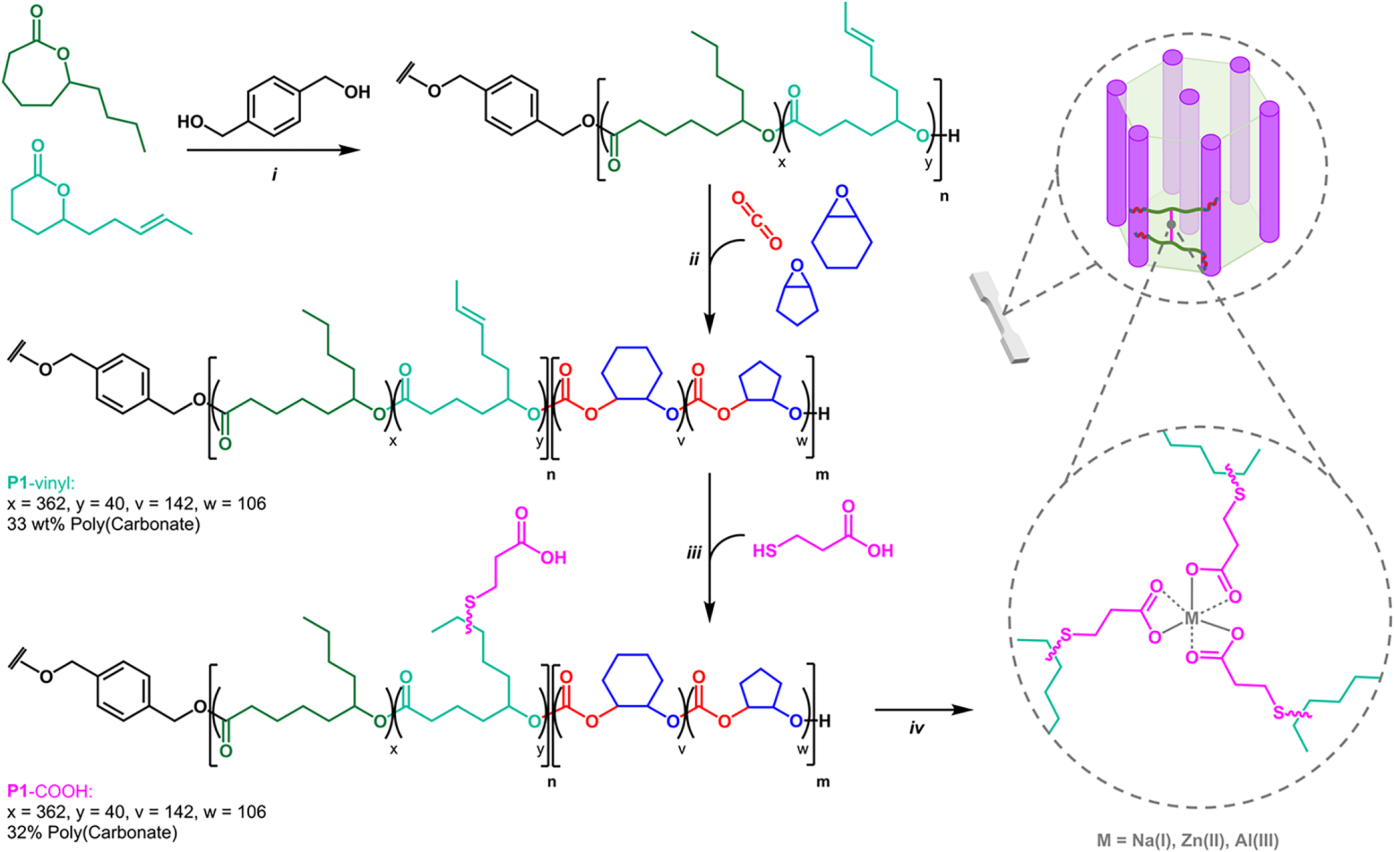
Synthesis of poly­(carbonate-*b*-ester-*b*-carbonate) ionomers. (i) DL/JL ROP at r.t catalyzed by [LZnMg­(C_6_F_5_)_2_] with benzene di-methanol (BDM) as bifunctional initiator, where [Cat]_0_/[BDM]_0_/[ε-DL]_0_/[δ-JL]_0_ = 1/2/740/82, [ε-DL and δ-JL]_0_ = 1.7 M, toluene. (ii) CO_2_/CHO/CPO ROCOP, CO_2_ (20 bar), 80 °C. (PCHC-*co*-PCPC)-*b*-(PDL-*co*-PJL)-*b*-(PCHC-*co*-PCPC) (**P1**-vinyl): *M*_n,SEC_ = 67.2 kg mol^–1^(*Đ*_M_ = 1.16), 33 wt % polycarbonate by ^1^H NMR spectroscopy. (iii) Thiol–ene functionalization (r.t., 1 h) with 3-mercaptopropionic (3-MPA) and DMPA photoinitiator, 365 nm, [**P1**-vinyl] = 5 wt %, THF. **P1**-COOH: *M*_n,SEC_ = 72.1 kg mol^–1^(*Đ*_M_ = 1.40), 32 wt % polycarbonate by ^1^H NMR spectroscopy. (iv) Coordination of Na­(I), Zn­(II), and Al­(III) to **P1**-COOH carboxylate ligands, **P1**-Na, **P1**-Zn, **P1**-Al. [**P1**-COOH] = 5 wt %, THF, NaOH, ZnEt_2_, AlEt_3_. [Na­(I)]/[COOH] = 1, [Zn­(II)]/[COOH] = 1/2, [Al]/[COOH] = 1/3.

By switching the reaction atmosphere from nitrogen to CO_2_ (20 bar), a mechanistic switch was activated, halting the lactone ROP and starting the ROCOP of CO_2_ and the epoxides. This resulted in the installation of the two outer polycarbonate A blocks and produced the desired ABA block polymer architecture. The poly­(cyclohexene carbonate-*co*-cyclopentene carbonate) (PCHC-*co*-PCPC) gradient terpolymer has been previously shown to have both a high glass transition temperature (*T*_g_) and a low entanglement molar mass (*M*_e_ = 10.7 kg mol^–1^), when synthesized from an equimolar mixture of CHO and CPO.^56^ Furthermore, while CPO and CHO are commonly petrochemical derived, they can also be prepared in on-pot chemo-enzymatic reactions from triglycerides.^57^ Consequently, PCHC-*co*-PCPC is a promising A block to produce high performance thermoplastic elastomers (TPEs).

The resulting triblock polymer (**P1**-vinyl) was produced on a 12 g scale and ^1^H NMR spectroscopy, after purification, established it comprised of the desired PDL/PJL of 90:10, PCHC/PCPC of 58:42, and, by comparing the integrals of characteristic signals for each repeat unit, an overall polycarbonate content of 33 wt % (Figure S3). Furthermore, by comparing these integrals to the aromatic signals of the initiator, the average DP values for (PCHC-*co*-PCPC)-*b*-(PDL-*co*-PJL)-*b*-(PCHC-*co*-PCPC) were determined to be (72–53)-(362–40)-(72–53).

The molar mass of **P1**-vinyl was moderately high (*M*_n,SEC_ = 67.2 kg mol^–1^) and narrowly disperse (*Đ*_M_ = 1.16) by size-exclusion chromatography (SEC, Figure S4). The ABA architecture was confirmed through end-group analysis, ^31^P­{^1^H} NMR spectroscopy after the addition of the a phospholane reagent, revealed the exclusive presence of PCHC and PCPC end-group corresponding to the outer polycarbonate blocks (Figure S5).^56,58^ 2D DOSY NMR spectroscopy displayed a single diffusion coefficient, further consistent with the formation of a block polymer (Figure S6). The double bond functionality in the PJL repeat units, installed throughout the central B block of the ABA block polymer, allows for precise functionalization. Specifically, the UV-initiated thiol–ene addition of 3-mercaptopropionic acid (3-MPA) to **P1**-vinyl. Functionalization was confirmed by the loss of vinyl signals at 5.39 ppm, paired with the emergence of signals at 2.62 and 2.76 ppm corresponding to the newly installed carboxylate ligands in the ^1^H NMR spectra (Figures S7–S9). Further analysis of the ^1^H NMR spectrum of the carboxylate functionalized polymer (**P1**-COOH) confirmed the slight decrease in polycarbonate content to 32 wt %. The molar mass of **P1**-COOH increased from the unfunctionalized precursor to *M*_n_ = 72.1 kg mol^–1^ with a slight broadening in dispersity (*Đ*_M_ = 1.40), attributed to interchain hydrogen bonding interactions (Figure S10). 2D DOSY NMR spectroscopy and ^31^P­{^1^H} NMR end-group analysis both confirm the retention of the ABA block architecture after functionalization (Figures S5 and S11).

Full-neutralization of the carboxylate ligands in **P1**-COOH and formation of the targeted Na­(I), Zn­(II), or Al­(III) ionomers was achieved through the addition of NaOH, ZnEt_2_, or AlEt_3_. This both allowed for precise control of the metal content in each ionomer and resulted in gaseous or liquid byproducts that were simply removed by drying *in vacuo*. Na­(I), Zn­(II), and Al­(III) were selected to explore the influence of metal valency on material properties and because they are abundent, lightweight, generally nontoxic, and produce colorless materials.

Stoichiometries of the metals added were adjusted, depending on the valency, to ensure each carboxylate ligand was coordinated to a metal, i.e., [Na­(I)]/[COOH] = 1, [Zn­(II)]/[COOH] = 1/2, [Al]/[COOH] = 1/3 (0.9, 1.7, and 0.4 wt % respectively). The ionomers **P1**-Na, **P1**-Zn and **P1**-Al were prepared on a 1.5 g scale, solvent cast (5 wt % THF), dried (120 °C, 72 h), and compression molded (140 °C, 1 tonne, 5 min) to produce thin films suitable for thermomechanical testing.

## Material Characterization

Metal complexation and ionomer formation were confirmed by Fourier Transform Infrared (FTIR) spectroscopy with the appearance of symmetric and asymmetric metal-carboxylate resonances at 1500 and 1650 cm^–1^ (Figure 2a). All polymeric materials were fully amorphous, with two glass transition temperatures (*T*_g_s) observed, by differential scanning calorimetry (DSC, Table 1 and Figure S12). The lower (−49 °C) and upper (104 °C) *T*_g_ observed for **P1**-vinyl are attributed to the central polyester and outer polycarbonate blocks, respectively, and are consistent with the values expected for each block, indicative of a phase separated microstructure.^18^ The upper *T*_g_ remains unchanged with the ionomer functionalization of the polyester block as there is no change to chain mobility within the polycarbonate domains. On the other hand, the *T*_g_ of the polyester block increases slightly following the carboxylic acid functionalization (−42 °C) with interchain hydrogen bonding restricting, somewhat, the macromolecular motion. Intriguingly, while DSC shows little apparent change in the lower *T*_g_ upon metal complexation, DMA reveals a modest increase, consistent with slightly restricted chain mobility despite the rapid and dynamic exchange of the metal-carboxylate cross-links (Table S1).

**Figure 2:**
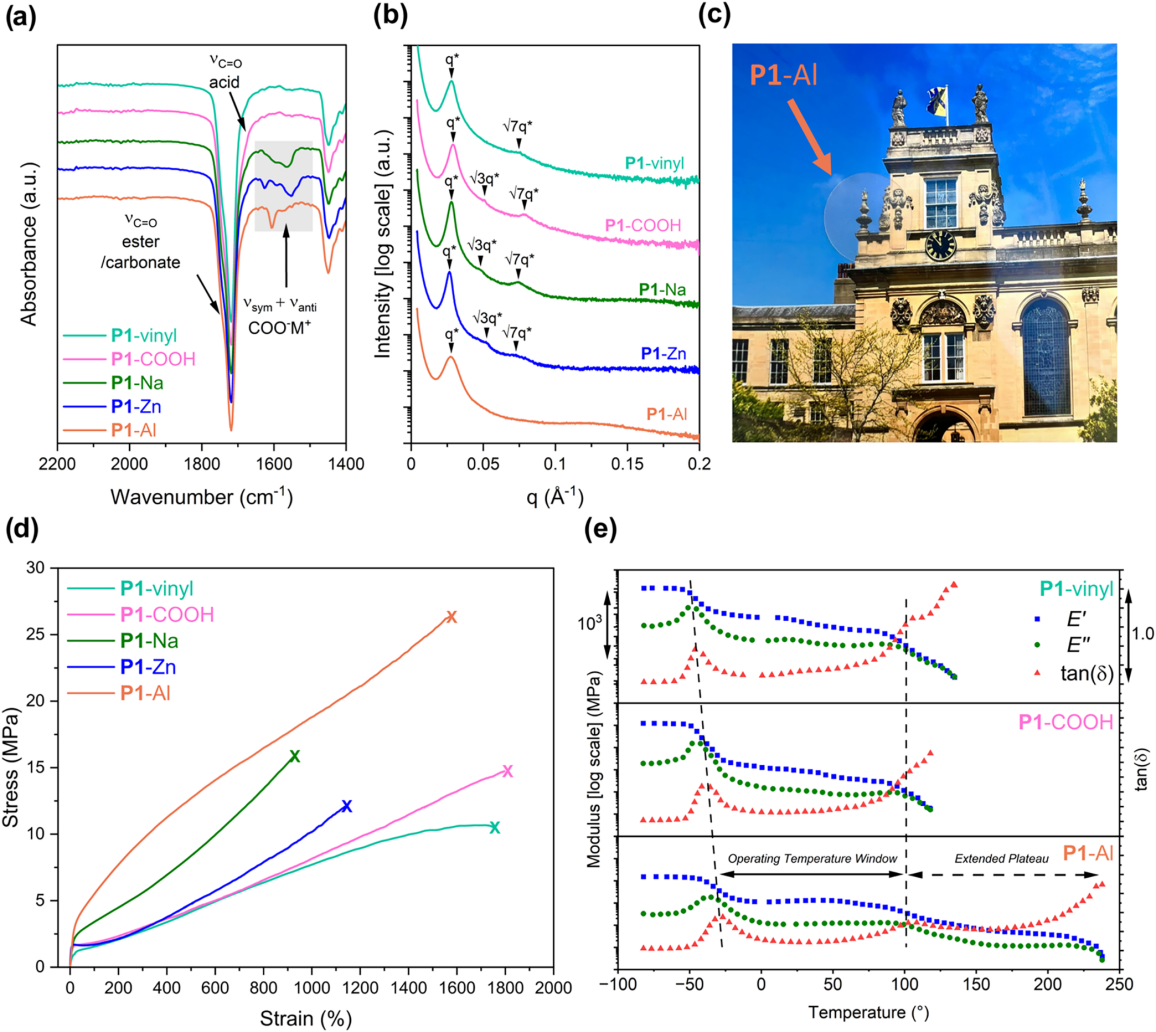
Characterization of ionomers. (a) FTIR spectra of polymer films. (b) Room-temperature small-angle X-ray scattering (SAXS) profiles showing principle scattering peaks (q*) and higher-order peaks (*q*/q*). (c) Digital photograph showing a transparent film of **P1**-Al superimposed upon a colored background. (d) Representative stress–strain curves (10 mm min^–1^ extension rate). (e) Dynamic mechanical analysis temperature sweeps (0.1% strain, 1 Hz, 3 °C min^–1^) for the unfunctionalized (**P1**-vinyl), carboxylate functionalized (**P1**-COOH), and aluminum ionomer (**P1**-Al).

**Table 1:** Summary of Thermomechanical Properties of CO_2_-Derived Block Polymer Elastomers

polymer	metal (wt % [mol %])	*T*_g1_, *T*_g2_ (°C)^a^	*E*_*y*_ (MPa)^b^	σ (MPa)^c^	ε_b_ (%)^d^	*U*_T_ (MJ m^–3^)^e^
P1-vinyl	0 [0]	–49, 104	33.3 ± 9.5	10.7 ± 0.8	1754 ± 81	134.4 ± 7.4
P1-COOH	0 [0]	–42, 102	30.1 ± 7.6	13.7 ± 0.7	1781 ± 88	126.8 ± 9.0
P1–Na	0.9 [6.1]	–42, 100	17.2 ± 2.7	15.7 ± 0.7	929 ± 54	74.7 ± 9.2
P1–Zn	1.7 [4.2]	–43, 103	68.7 ± 1.6	12.2 ± 0.3	1157 ± 33	59.6 ± 7.2
P1–Al	0.4 [2.1]	–42, 100	28.5 ± 5.4	26.2 ± 1.4	1554 ± 74	241.0 ± 18.4

a Lower and upper glass transition temperature from DSC.

b Young’s modulus.

c Ultimate tensile strength.

d Strain at break.

e Tensile toughness (area under the stress–strain curve). Mean values ± std. dev. from measurements conducted independently on 5 specimens.

Small-angle X-ray scattering (SAXS) was employed to study the microphase separated morphology of the block polymers (Figure 2b). Scattering patterns were collected without any pretreatment and at room temperature. After indexing the higher order peaks relative to the principal scattering peak (q*), **P1**-vinyl, **P1**-COOH, **P1**-Na, and **P1**-Zn were found to adopt a hexagonally packed cylindrical morphology (*q*/q* = √3, √7). No high order scattering peaks were observed for **P1**-Al but it displays a principal scattering peak, consistent with a phase separated microstructure. The domain sizes (*d* = 2π/q*) did not change significantly upon installation of the carboxylic acids or complexation of the metals, remaining between 22 and 24 nm (Table S2). These results suggests that any differences in thermomechanical properties between the materials is a result of differing ionomeric cross-linking and not morphology. Circular specimens of each elastomer (8 mm diameter, ∼0.25 mm thickness) were subjected to solvent swelling experiments to establish a qualitative understanding of the cross-linking density. After submersion in THF for 24 h, **P1**-vinyl, **P1**-COOH, **P1**-Na, and **P1**-Zn all completely dissolved while **P1**-Al swelled to 458% ± 26% of its original weight. After dying *in vacuo* a gel content of 94% ± 4% was determined. These results suggest that the sodium and zinc ionomers can be solubilized due to their lower effective cross-link densities, arising from monovalent Na­(I) and divalent Zn­(II) coordination. In contrast, the solvent resistance of **P1**-Al arises from the trivalent nature of Al­(III), where each metal center can form up to three metal-carboxylate cross-links; complete solubilization therefore requires dissociation of more metal-carboxylate interactions, leading macroscopically to network swelling rather than dissolution.The colorless and optically transparent films (Figure 2c) were fabricated and cut into dumbbell specimens (ISO 527) suitable for uniaxial tensile testing to assess the impact of dynamic ionomeric cross-linking on mechanical performance (Figure 2d and Table 1). All materials exhibited a diffuse yield point and behaved as tough elastomers. The unfunctionalized ABA block polymer **P1**-vinyl exhibited tensile mechanical properties matching those of styrenic elastomers, with an ultimate tensile strength (σ) of 10.7 MPa and a strain at break (ε) of 1754%.^59^ This is attributed to the polycarbonate entanglements present in the glassy domains. Installation of the carboxylate ligands and formation of **P1**-COOH did not result in a significant change in ε however the σ did increase to 13.7 MPa. The increased tensile strength is attributed to the introduction of interchain hydrogen bonding interactions.

Metal complexation by the carboxylate groups facilitated the further improvement of mechanical performance. **P1**-Na and **P1**-Zn both exhibited an increase in σ, a slight reduction in ε, and greater strain hardening relative to **P1**-vinyl. These changes in mechanical properties are ascribed to the dynamic but strong ionomeric cross-links which act as quasi-entanglements. Monovalent Na­(I) is known to form ionic clusters in carboxylate-based ionomers, which increases the effective cross-linking density and contributes to the higher tensile strength observed for **P1**-Na (15.7 MPa) relative to **P1**-Zn (12.2 MPa).^36^ Despite this, **P1**-Zn exhibits the greatest Young’s modulus (*E*_*y*_ = 68.7 MPa). The observed mechanical and strain-rate-dependent behavior (*vide infra*) is consistent with Zn­(II)-carboxylate cross-links exhibiting longer effective lifetimes and slower relaxation dynamics within the polymer network than those associated with Al­(III) or Na­(I). We believe that these transient but more kinetically stable cross-links act as elastically active constraints on the time scale of small-strain deformation, leading to increased stiffness even at lower effective cross-link densities. **P1**-Al displayed the greatest tensile strength (26.2 MPa) of the materials reported and also showed high elasticity. Consequently, the aluminum ionomer is 80% tougher than **P1**-vinyl. These impressive tensile mechanical properties are likely due to both the high cross-linking density, resulting from the use of a trivalent metal, and the dynamic nature of the cross-linking. The repeated breaking and reformation of the metal–ionomer cross-linking provides a mechanism for stress dissipation, resulting in significant toughening of the material.^60^ This result is in contrast to previously reported materials with identical ionomeric cross-linking in the glass polycarbonate domains.^32–34^ In that case, the limited chain mobility at room temperature restricted any dynamism, resulting in stronger but less ductile materials.

In order to study the thermomechanical profile of the ionomers, dynamic mechanical temperature sweeps were performed under tension (Figures 2e and S13–S15). The operating temperature window (OTW, the difference between the upper and lower *T*_g_ values) was greatest for **P1**-vinyl, from −47 °C to 104 °C.

It remains very broad with **P1**-COOH with the lower *T*_g_ increasing slightly to −42 °C and to −34 °C, −35 °C, and −32 °C upon coordination to Na­(I), Zn­(II), and Al­(III) respectively, agreeing well with the trends observed by DSC (Table 1). Despite this slight increase in the lower *T*_g_, the OTW remained wide ranging after functionalization. Owing to the high cross-linking density, **P1**-Al exhibited an extended plateau region above the upper *T*_g_, retaining some strength to 238 °C. The same phenomenon could not be observed for **P1**-Na or **P1**-Zn. As **P1**-Al is above both block polymer *T*_g_ values in the extended plateau, the mechanical response results from the aluminum-carboxylate cross-links. This high temperature region can be used to estimate the length of the macromolecules between cross-links within the ionomer (*M*_COOAl_). This cross-linking density was found to be 13.6 kg mol^–1^. To determine and subsequently compare this value with the *M*_e_ of PJL-*co*-PDL, a 100.9 kg mol^–1^ sample containing 12 mol % PJL was synthesized, rheological frequency sweeps were measured between 15 and 60 °C at 5 °C intervals, and a time–temperature superposition (TTS) master curve constructed. The *M*_e_ of PJL-*co*-PDL was found to be between 4.5 and 6.2 kg mol^–1^ (Figures S16–S18). Therefore, the number of effective entanglements in the material increases 33–46% with the incorporation of even 0.4 wt % aluminum, contributing to the significant improvement in mechanical properties observed.

To assess the elastic performance, cyclic tensile testing (200% strain, 10 cycles, 10 mm min^–1^) was performed on specimens of each material (Figures 3a and S19–S43). The elastic recovery (the ability of a material to recover its original dimensions after deformation), resilience (the ability of a material to recover energy after deformation), and residual strain (permeant deformation after the removal of stress) were all assessed over the course of the 10 cycles. While resilience decreased and residual strain increased moderately upon functionalization, all materials maintained impressive elastic recovery (>85%). To begin to probe the dynamic ionomeric cross-linking, the cycling tensile testing was repeated at 2, 5, 50, and 100 mm min^–1^ in order to explore any strain-rate dependent behavior (Figure 3b). **P1**-vinyl, **P1**-COOH, and **P1**-Al display elastomeric properties independent of strain-rate. On the other hand, the elastic recovery, residual strain, and resilience worsen with increasing strain-rate for **P1**-Na and **P1**-Zn.

**Figure 3:**
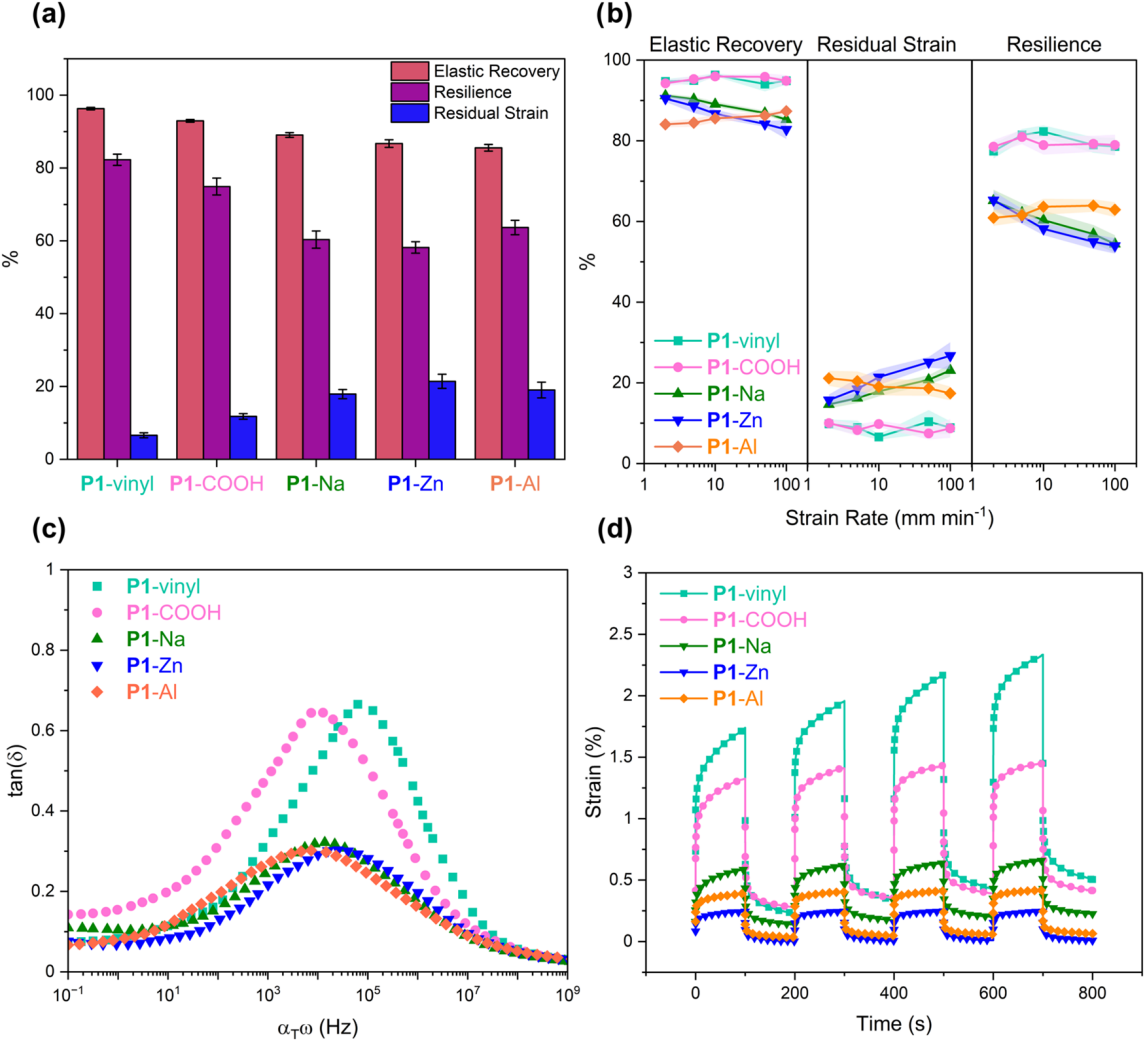
Viscoelastic properties. (a) Average elastic recovery, resilience, and residual strain from cyclic tensile testing (200%, 10 cycles). (b) Strain-rate dependence of elastic properties. (c) Time–temperature superposition (TTS) master curve tan­(δ) profiles plotted against shifted frequency (α_T_ω, *T*_ref_ = −10 °C). (d) Creep-recovery experiments at 30 °C, 5 kPa.

In order to better understand the strain-rate dependence observed and to probe the effect of the dynamic ionomer cross-linking, TTS master curves were constructed from a series of low-temperature frequency sweeps performed on each material under tension (Figures 3c and S44–S48). Specifically, these low-temperature oscillatory DMA experiments help inform upon the behavior of the PDL-*co*-PJL block in the viscoelastic transition region. The tan­(δ) profile, a measure of the relative degree of energy dissipation, allows us to determine both the amount of damping in the materials (tan­(δ)_max_) and the frequency (α_T_ω_peak_) of the glass transition at the reference temperature (*T*_ref_ = −10 °C) (Table S3).

**P1**-COOH exhibits a significant decrease in α_T_ω_peak_ relative to the unfunctionalized **P1**-vinyl­(1.0 × 10^4^ and 8.0 × 10^4^ Hz, respectively). Following the trend in lower *T*_g_ values, this does not significantly decrease further with the incorporation of the ionomeric cross-links, providing more evidence of dynamic metal–ligand exchange. The value of tan­(δ)_max_ does drop from 0.65 for **P1**-vinyl and **P1**-COOH, to 0.30 for **P1**-Na, **P1**-Zn, and **P1**-Al. This reflects the incorporation of stronger ionomeric cross-linking, resulting in reduced damping. The strain-rate invariant behavior of **P1**-vinyl and **P1**-COOH is attributed to this greater ability to dissipate energy. Conversely, the poor damping in **P1**-Na and **P1**-Zn is attributed to the worsening elastic performance at high strain-rates. While **P1**-Al also displays less effective energy dissipation, its strain-rate independent behavior is attributed to the high cross-linking density when trivalent Al­(III) is utilized in the material.^61^ As seen in both the swelling and DMA experiments, **P1**-Al displays a greater cross-linking density. Both this cross-linking density and the dynamic and reversible nature of the metal-carboxylate interactions give rise to the improved toughness.

Critical to the application of any engineering elastomer is the ability to retain its size and shape under an applied load, dimensional stability. Creep-recovery experiments (5 kPa, 100 s, 4 cycles) were conducted for all materials to examine the effect of functionalization on creep-resistance. **P1**-vinyl exhibited 1.7% strain after the first loading cycle which increased to 2.3% by the final cycle (Figure 3d). **P1**-COOH experienced a reduction to 1.3% which did not rise with repeated loading and unloading. The dimensional stability continued to improve upon formation of the ionomers. **P1**-Na and **P1**-Al showed very low, 0.66% and 0.42%, deformation by the final loading cycle. **P1**-Zn displayed the greatest creep resistance, reaching only 0.24% strain on the final cycle. This enhanced creep resistance in **P1**-Zn reflects the longer lifetime and slower exchange of Zn­(II)-carboxylate coordination bonds. These results highlight the improved dimensional stability gained through the introduction of ionomeric cross-links to the central rubbery block of TPEs.

It is also essential that engineering materials are resistant to a range of environmental conditions. To access how mechanical properties may vary in humid environments, 50 min oscillatory time sweeps (30 °C, 0.1% strain, 1 Hz) were performed at 10, 30, 50, 70, and 90% relative humidity (Figures S49–S53). There was no change in the storage or loss moduli for any of the materials over the course of the experiments, highlighting the that at low levels of functionalization (0.4–1.7 wt % metal content), the material’s hydrophobicity is not significantly altered.

Thermal stability is also critical to material performance and processing. Thermal gravimetric analysis (TGA) was carried out on samples of each material (Figures S50–S54). Iso-thermal experiments demonstrate that all polymers exhibit excellent stability at 160 °C, with negligible mass loss over 50 min, and onset of depolymerization does not occur until temperatures approach ∼180 °C (Figure S57). Although dynamic TGA temperature ramps yield *T*_d,5%_ values above 250 °C for all samples (Table S3), this metric reflects the cumulative effects of both metal catalyzed depolymerization and thermal degradation under continuous heating for the ionomers and should not be interpreted as a true degradation onset. Importantly, the polymers remain stable under the processing conditions used for the ionomers (140 °C, 1 tonne, 5 min), providing a wide and practical (re)­processing temperature window.

## Mechanical and Chemical Recycling

It is essential that the next generation of sustainable materials possess multiple end-of-life options. Mechanical recycling is critical to reduce the emissions associated with the constant production of virgin grade materials, to extend the lifetime of a material, and to minimize the environmental impacts of polymer waste. **P1**-Al was selected to study the laboratory-scale reprocessability of these ionomers under mild conditions, as it is the only material which cannot be repeatedly solvent cast and, therefore must be reprocessed by compression molding (Figure S60). After three rounds of mechanical recycling there was no significant decrease in σ, *E*_Y_, or tensile toughness. There was, however, a slight decrease in the strain at break between the virgin material and third reprocessing cycle but the value remained above 1200%, far beyond the requirements for most elastomers (Table S5 and Figure S61).

During industrial scale mechanical recycling, it is increasingly difficult to stop the incorporation of impurities into the material and indefinite reprocessing is not viable. Therefore, it is essential materials can also undergo chemical recycling back to repolymerizable monomers, allowing for the repeated production of virgin grade materials. Recent studies have employed both specialized catalysts and simple metal salts to chemically recycle polycarbonates to CO_2_ and epoxides or cyclic carbonates, as well as polyesters to lactones.^50,52^ In each case, a catalytic species is added when the chemical recycling is desired. Here, we wanted to exploit the ionomer metals, used to improve mechanical performance, to serve a second crucial role: to act as an embedded depolymerization catalyst.

To assess their for potential as self-depolymerizing materials, all elastomers were subjected to 50 minute isothermal TGA experiments, at 160, 180, 200, and 220 °C (Figure S62). All three ionomers exhibited a significantly greater mass loss relative to **P1**-vinyl and **P1**-COOH, indicative of catalyzed depolymerization occurring. Of all the materials, **P1**-Zn exhibited the greatest mass loss after the series of isothermal experiments (62%) as well as the greatest rate of depolymerization at 200 °C (TOF = 1.5 h^–1^) (Figure S63). While **P1**-Na and **P1**-Al exhibited self-catalyzed depolymerization, the greater rates of depolymerization for **P1**-Zn allow for a better exploration of the integrated circularity. Twelve-hour isothermal TGA experiments at 200 °C, resulted in a mass loss of 83% for **P1**-Zn, compared with only a 14% mass loss for **P1**-vinyl (Figure S64).

To better probe the depolymerization and to determine the products formed, TGA-FTIR spectroscopy was conducted (Figure 4B). The TGA thermograms changed significantly between **P1**-vinyl and **P1**-Zn: the onset of mass loss dropped from 273 to 244 °C and the change in thermogram profile indicated the mass loss was occurring by different mechanisms. The FTIR spectrum for **P1**-Zn at 260 °C revealed the loss of both CO_2_ and a cyclic carbonate species (Figures 4C and S65).

**Figure 4:**
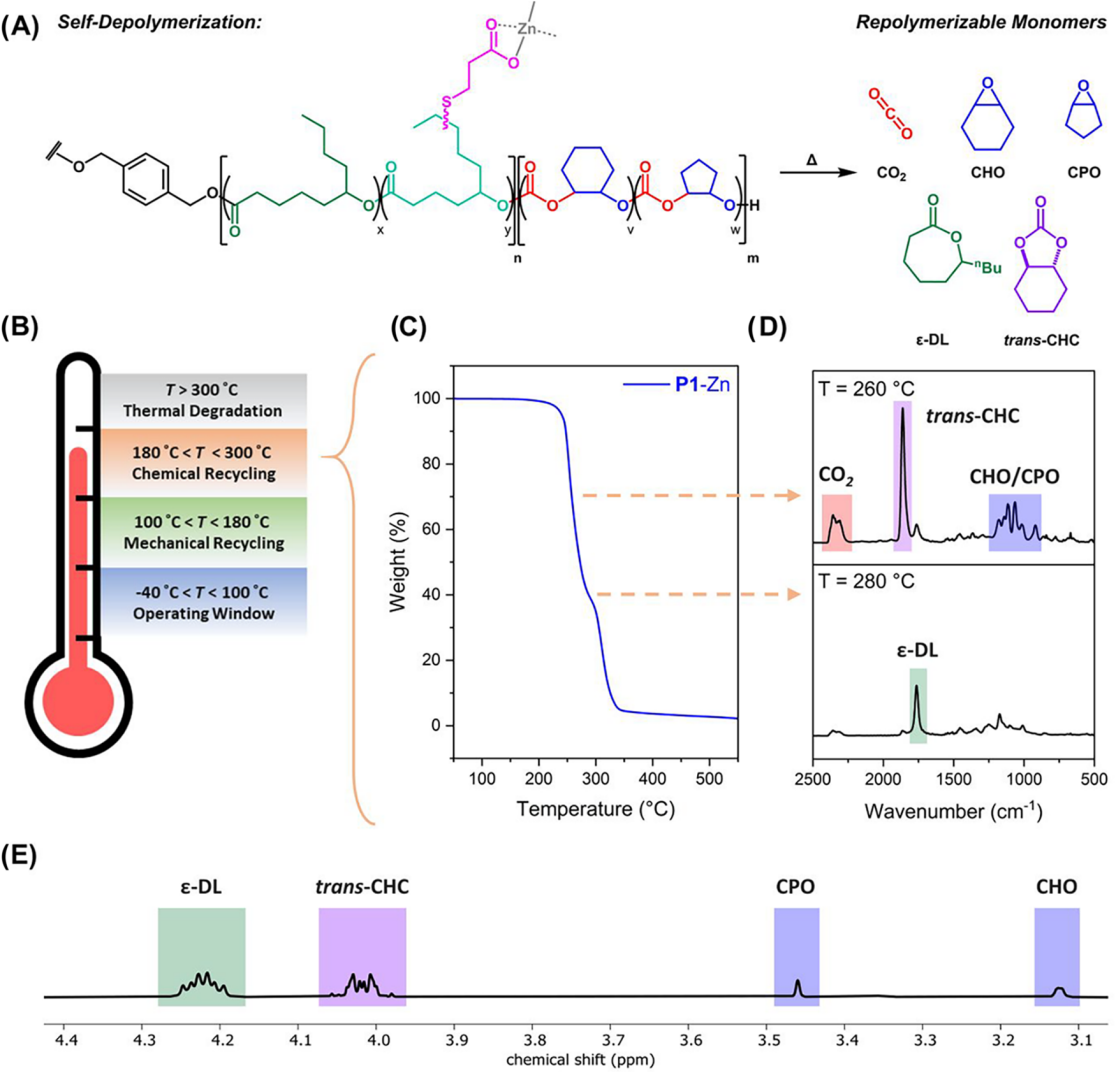
Chemical recycling by ionomer self-depolymerization. (A) Schematic of **P1**-Zn chemical recycling to monomers. (B) Illustration of operating and recycling temperature windows for ionomers (C) TGA thermograms (5 °C min^–1^) for and **P1**-Zn. (D) TGA-FTIR spectra for **P1**-Zn at 260 and 280 °C. (E) ^1^H NMR spectrum of mixture of the monomers after **P1**-Zn recycling.

Therefore, the depolymerization is expected to occur through a chain-end mechanism, with conversion of the outer polycarbonate blocks occurring first to form a mixture of cyclic carbonate, epoxides, and CO_2_. Next, the depolymerization of the central polyester block to ε-DL occurs at 280 °C, where only the lactone is observed. The ring-opened hydroxy acid (derived from δ-JL and/or ε-DL) is only observed at temperatures exceeding 300 °C, which correspond to conditions where polyesters undergo thermal degradation rather than controlled depolymerization (Figure S66). The residual 1.9 wt % at the end of the recycling process matches the expected 1.7 wt % zinc content in the ionomer. The chemical recycling of **P1**-Zn was repeated on a 0.76 g scale (200 °C, 10 mbar) with the products removed (by distillation) as the reaction progressed (Figure S67). After 24 h, the reaction was cooled and ^1^H NMR spectroscopy was employed to study the products (Figures 4D and S64). This revealed the formation of a mixture of ε-DL, *trans*-cyclohexene carbonate (*trans*-CHC), cyclopentene oxide, and cyclohexene oxide, collected in an overall 77% yield. The selectivity for *trans*-CHC (vs. CHO), was 83%. There was no evidence of the formation of *trans*- or *cis*-cyclopentene carbonate, indicating >99% selectivity for CPO.^48^ The epoxides and ε-DL were separated by fractional distillation and collected in a 77% yield (Figure S68 and Table S6). The chemically recycled monomers were combined with some additional monomers to reproduce **P1**-vinyl (re**P1**-vinyl). Overall, 3% of the epoxide and 14% of the ε-DL utilized in the repolymerization were collected from the self-depolymerization. These yields are unoptimized and currently limited by both the quantity of **P1**-Zn available for chemical recycling and the minimum fill volume of the high-pressure CO_2_ reactor. With greater quantities of **P1**-Zn and/or smaller reactors, the fraction of recyclate could be further increased. re**P1**-vinyl was successfully synthesized with a 34 wt % polycarbonate content, PJL/PDL = 12:88, and an overall molar mass of 30.8 kg mol^–1^, (*Đ*_M_ = 1.68, Figure S69).

It is essential that the self-depolymerization of the ionomers is possible in the presence of common impurities found in polymer waste recycling streams. Therefore, polyethylene (PE), polypropylene (PP), polystyrene (PS), and polyethylene terephthalate (PET) samples were collected from recycling waste in our office and laboratory, shredded to a fine powder, and physically blended (50:50 wt %) with **P1**-Zn (Figure S70). TGA-FTIR experiments confirmed that the recycling to the monomers was feasible even in the presence of the other polymer impurities (Figures S71–S75). Furthermore, the chemical recycling catalysis proceeded in a quinary blend of **P1**-Zn, PE, PP, PS, and PET, with the thermal degradation of the polymer impurities unchanged by the presence of the ionomer (Figures S76–S79). These results underline the versatility of the dual functional ionomer approach to toughen and recycle sustainable polymers.

All of the monomers collected can be repolymerized to form virgin grade materials, helping to facilitate a circular plastic economy without the need to introduce any additional depolymerization catalysts. The zinc introduced to strengthen and stiffen the materials also serves a second crucial role within the polymer: to catalyze self-depolymerization to the constituent monomers. We anticipate that this concept should be generalizable to other epoxide, heteroallene, lactone, and diolide monomers and metal combinations, to yield a suite of recyclable materials with wide-ranging material properties. The dual-function material design both advances the development of sustainable, high-performance elastomers and establishes the potential for future polymer innovations that integrate toughness, reprocessability, and chemical recyclability using earth abundant ionomers.

## Supplementary Material



## Data Availability

Experimental data: The data that supports the findings of this study are open-access at: https://ora.ox.ac.uk/objects/uuid:3f12f976-0318-4dc4-9ea7-3ec4c1c7dcc0

## Notes

Detailed synthetic and characterization (NMR, SEC, DSC, TGA, DMA, uniaxial and cyclic tensile) procedures, instrument details, and ISO standards used for materials testing (PDF)
