# Supplementary material for: Function Meets Circularity: Metal–Ionomer Cross-Links Toughen and Recycle CO2‑Derived Polymers
Source: Macromolecules. 2026 Mar 12;59(6):3649–59. doi: 10.1021/acs.macromol.6c00188 (PMC13019675; doi:10.1021/acs.macromol.6c00188)
Supplement: Supplementary file 1 [file ma6c00188_si_001.pdf]

Supporting Information

Function Meets Circularity: Metal-Ionomer Crosslinks Toughen and Recycle CO_2-Derived Polymers

Kam C. Poon, Thomas M. McGuire, Chang Gao, Gregory S. Sulley, and Charlotte K. Williams*

K. C. Poon, T. M. McGuire, C. Gao, G. S. Sulley, & C. K. Williams

Chemistry Research Laboratory, Department of Chemistry, University of Oxford, Oxford, OX1 3TA, U.K.

E-mail: charlotte.williams@chem.ox.ac.uk

Experimental Dataset:

https://ora.ox.ac.uk/objects/uuid:3f12f976-0318-4dc4-9ea7-3ec4c1c7dcc0

Contents

Experimental Details 6

Reagents and Methods 8

Figure S1: Quantitative $^{13}C\{^1H\}$ NMR (150 MHz, $CDCl_3$) spectra of ε-DL/δ-JL copolymerization aliquots (Diethyl carbonate used as an internal standard). 10

Meyer-Lowry Model(*5*): Compositional drift in f_{DL} fitted to toal conversion χ_n 10

Figure S2: Meyer-Lowry fit to ε-DL/δ-JL copolymerization data. 11

Figure S3: ^1H NMR (400 MHz, $CDCl_3$) spectrum of **P1**-vinyl. 11

Figure S4: SEC (THF, 1 mL min^{-1}) traces for **P1**-vinyl. The instrument is calibrated with poly(styrene) standards. 12

Figure S5: $^{31}P\{^1H\}$ NMR end-group analysis of **P1**-vinyl (teal) and **P1**-COOH (pink). 12

Figure S6: ^1H DOSY NMR (400 MHz, $CDCl_3$) spectrum of **P1**-vinyl. 13

Figure S7: ^1H NMR (400 MHz, $CDCl_3$) spectrum of **P1**-COOH. 13

Figure S8: ^1H NMR (400 MHz, $CDCl_3$) spectra of **P1**-vinyl and **P1**-COOH highlighting loss of vinyl signals after post-polymerization functionalization. 14

Figure S9: $^{13}C\{^1H\}$ NMR (150 MHz, $CDCl_3$) spectra of **P1**-vinyl and **P1**-COOH highlighting loss of vinyl signals after post-polymerization functionalization and broadening of carbonyl signal. 14

Figure S10: SEC (THF, 1 mL min^{-1}) traces for **P1**-COOH: a post reaction aliquot (solid) and following precipitation and drying (dashed). The instrument is calibrated with poly(styrene) standards. 15

Figure S11: ^1H DOSY NMR (400 MHz, $CDCl_3$) spectrum of **P1**-vinyl. 16

Figure S12: Differential Scanning Calorimetry (DSC) traces of **P1**-vinyl, **P1**-COOH., **P1**-Na, **P1**-Zn, and **P1**-Al. 16

Table S1: Summary of lower $T_{g,DMA}$ values 17

Table S2: Summary of SAXS Data. 17

Figure S13: Dynamic mechanical analysis temperature sweeps (0.1% strain, 1 Hz, 3˚C min^{-1}) for **P1**-Na. 18

Figure S14: Dynamic mechanical analysis temperature sweeps (0.1% strain, 1 Hz, 3˚C min^{-1}) for **P1**-Zn. 18

Figure S15: Dynamic mechanical analysis temperature sweeps (0.1% strain, 1 Hz, 3˚C min^{-1}) for virgin **P1**-Al and aged 352 days after mechanical recycling experiments. 19

Figure S16: ^1H NMR (400 MHz, $CDCl_3$) spectrum of PJL-*co*-PDL. 20

Figure S17: SEC (THF, 1 mL min^{-1}) traces for PJL-*co*-PDL. The instrument is calibrated with poly(styrene) standards. 20

Figure S18: Rheological time-temperature superposition master curve for PJL-*co*-PDL. Melt density assumed to be between 800 and 1100 kg m^{-3}. The effective molecular weight between constraints was estimated by assuming additive contributions from entanglements and dynamic crosslinks $1/M\mathrm{eff} = 1/Me + 1/MCOOAl$. 21

Figure S19: Uniaxial cyclic tensile testing (200% strain, 10 cycles) at 2 mm min^{-1} for **P1**-vinyl. 22

Figure S20: Uniaxial cyclic tensile testing (200% strain, 10 cycles) at 5 mm min^{-1} for **P1**-vinyl. 22

Figure S21: Uniaxial cyclic tensile testing (200% strain, 10 cycles) at 10 mm min^{-1} for **P1**-vinyl. 23
Figure S22: Uniaxial cyclic tensile testing (200% strain, 10 cycles) at 50 mm min^{-1} for **P1**-vinyl. 23
Figure S23: Uniaxial cyclic tensile testing (200% strain, 10 cycles) at 100 mm min^{-1} for **P1**-vinyl. 24
Figure S24: Uniaxial cyclic tensile testing (200% strain, 10 cycles) at 2 mm min^{-1} for **P1**-COOH. 24
Figure S25: Uniaxial cyclic tensile testing (200% strain, 10 cycles) at 5 mm min^{-1} for **P1**-COOH. 25
Figure S26: Uniaxial cyclic tensile testing (200% strain, 10 cycles) at 10 mm min^{-1} for **P1**-COOH. 25
Figure S27: Uniaxial cyclic tensile testing (200% strain, 10 cycles) at 50 mm min^{-1} for **P1**-COOH. 26
Figure S28: Uniaxial cyclic tensile testing (200% strain, 10 cycles) at 100 mm min^{-1} for **P1**-COOH. 26
Figure S29: Uniaxial cyclic tensile testing (200% strain, 10 cycles) at 2 mm min^{-1} for **P1**-Na. 27
Figure S30: Uniaxial cyclic tensile testing (200% strain, 10 cycles) at 5 mm min^{-1} for **P1**-Na. 27
Figure S31: Uniaxial cyclic tensile testing (200% strain, 10 cycles) at 10 mm min^{-1} for **P1**-Na. 28
Figure S32: Uniaxial cyclic tensile testing (200% strain, 10 cycles) at 50 mm min^{-1} for **P1**-Na. 28
Figure S33: Uniaxial cyclic tensile testing (200% strain, 10 cycles) at 100 mm min^{-1} for **P1**-Na. 29
Figure S34: Uniaxial cyclic tensile testing (200% strain, 10 cycles) at 2 mm min^{-1} for **P1**-Zn. 29
Figure S35: Uniaxial cyclic tensile testing (200% strain, 10 cycles) at 5 mm min^{-1} for **P1**-Zn. 30
Figure S36: Uniaxial cyclic tensile testing (200% strain, 10 cycles) at 10 mm min^{-1} for **P1**-Zn. 30
Figure S37: Uniaxial cyclic tensile testing (200% strain, 10 cycles) at 50 mm min^{-1} for **P1**-Zn. 31
Figure S38: Uniaxial cyclic tensile testing (200% strain, 10 cycles) at 100 mm min^{-1} for **P1**-Zn. 31
Figure S39: Uniaxial cyclic tensile testing (200% strain, 10 cycles) at 2 mm min^{-1} for **P1**-Al. 32
Figure S40: Uniaxial cyclic tensile testing (200% strain, 10 cycles) at 5 mm min^{-1} for **P1**-Al. 32
Figure S41: Uniaxial cyclic tensile testing (200% strain, 10 cycles) at 10 mm min^{-1} for **P1**-Al. 33
Figure S42: Uniaxial cyclic tensile testing (200% strain, 10 cycles) at 50 mm min^{-1} for **P1**-Al. 33
Figure S43: Uniaxial cyclic tensile testing (200% strain, 10 cycles) at 100 mm min^{-1} for **P1**-Al. 34
Figure S44: Time-temperature superposition master curve for **P1**-vinyl. 34
Figure S45: Time-temperature superposition master curve for **P1**-COOH. 35
Figure S46: Time-temperature superposition master curve for **P1**-Na. 35
Figure S47: Time-temperature superposition master curve for **P1**-Zn. 36
Figure S48: Time-temperature superposition master curve for **P1**-Al. 36
Table S3: Summary of TTS tan(δ) peaks. 37
Figure S49: DMA relative humidity (RH) time sweeps for **P1**-vinyl. 37
Figure S50: DMA relative humidity (RH) time sweeps for **P1**-COOH. 38
Figure S51: DMA relative humidity (RH) time sweeps for **P1**-Na. 38
Figure S52: DMA relative humidity (RH) time sweeps for **P1**-Zn. 39
Figure S53: DMA relative humidity (RH) time sweeps for **P1**-Al. 39
Figure S54: TGA profile for **P1**-vinyl. 40
Figure S55: TGA profile for **P1**-COOH. 40

Figure S56: TGA profile for **P1**-Na. 41

Figure S57: TGA profile for **P1**-Zn. 41

Figure S58: TGA profile for **P1**-Al. 42

Table S4: Temperature at 5% mass loss during a 10 ˚C/min heating ramp. 42

Figure S59: Self-depolymerization screening isothermal TGA experiments. 43

Figure S60: Representative stress-strain curves (10 mm min^{-1} extension rate) for virgin **P1**-Al and after each of three thermal reprocessing (compression moulding) cycles. 43

Table S5: Summary of tensile mechanical properties after mechanical recycling. 44

Figure S61: Summary of tensile mechanical properties after mechanical recycling. 44

Figure S62: Conversion during self-depolymerization screening. 45

Figure S63: Rate of depolymerization during self-depolymerization screening. 45

Figure S64: Isothermal TGA experiment (200 ˚C, 12 h). 46

Figure S65: FTIR-TGA standards for expected monomers. 46

Figure S66: a) FTIR-TGA thermogram with associated Gram-Schmidt profile for **P1**-Zn. b) FTIR spectrum of exhaust gas at 260, 280, and 325 ˚C. 47

Figure S67: Digital photograph of 0.76 g scale self-depolymerization of **P1**-Zn. 48

Figure S68: 1H NMR (400 MHz, CDCl3) spectrum of mixture of the monomers after P1-Zn recycling, CHO/CPO and ε-DL separated by fractional distillation (110 ˚C and N_2,120 ˚C and 1.9 x 10-1 mbar atmosphere, respectively), and **P1**-Zn resynthesized from the chemically recycled monomers. 49

Table S6: Summary of chemical recycling results 50

Figure S69: SEC (THF, 1 mL min-1) traces for re**P1**-vinyl. The instrument is calibrated with poly(styrene) standards. 50

Figure S70: Digital photograph of waste polymers collected from laboratory and office recycling bins (polyethylene (PE), polypropylene (PP), polystyrene (PS), and polyethylene terephthalate (PET). Polymers were all shredded to form physical blends. 51

Figure S71: A) TGA thermograms (5 ˚C min^{-1}) for **P1**-Zn and polyethylene (PE) blend (50:50 wt%). B) TGA-FTIR spectra for **P1**-Zn at 260 and 280 ˚C. 52

Figure S72: A) TGA thermograms (5 ˚C min^{-1}) for **P1**-Zn and polypropylene (PP) blend (50:50 wt%). B) TGA-FTIR spectra for **P1**-Zn at 260 and 280 ˚C, showing lactone (green) and *trans*-CHC (purple). 53

Figure S73: A) TGA thermograms (5 ˚C min^{-1}) for **P1**-Zn and polystyrene (PS) blend (50:50 wt%). B) TGA-FTIR spectra for **P1**-Zn at 260 and 280 ˚C, showing lactone (green) and *trans*-CHC (purple). 54

Figure S74: A) TGA thermograms (5 ˚C min^{-1}) for **P1**-Zn and polyethylene terephthalate (PET) blend (50:50 wt%). B) TGA-FTIR spectra for **P1**-Zn at 260 and 280 ˚C, showing lactone (green) and *trans*-CHC (purple). 55

Figure S75: A) TGA thermograms (5 ˚C min^{-1}) for **P1**-Zn and polyethylene, polypropylene (PP), polystyrene (PS), and polyethylene terephthalate (PET) blend (50:12.5:12.5:12.5:12.5 wt%). B) TGA-FTIR spectra for **P1**-Zn at 260 and 280 ˚C, showing lactone (green) and *trans*-CHC (purple). 56

Figure S76: TGA profile for PE 57

Figure S77: TGA profile for PP. 57

Figure S78: TGA profile for PS. 58
Figure S79: TGA profile for PET. 58

Experimental Details

NMR Spectroscopy. ^{1}H and $^{31}P\{^{1}H\}$ NMR spectra were obtained using a Bruker AVIII HD 400 NMR spectrometer. ^{13}C $\{^{1}H\}$ NMR spectra were obtained using a Bruker Avance III AVD500 NMR spectrometer. ^{1}H DOSY spectra were obtained using a Bruker NEO600 NMR spectrometer. δ = gradient pulse length = 0.004 s. Δ = diffusion delay = 0.1941 s. DOSY spectra were processed using Mnova's Bayesian DOSY algorithm (version 16.0.0), which infers diffusion coefficients without imposing a single- or multi-component exponential model.

Size Exclusion Chromatography (SEC). Polymers (2-5 mg) were dissolved in THF. Samples were passed through 0.2 µm PTFE filters prior to analysis. Analysis was carried out on a Shimadzu LC-20AD instrument, equipped with a Refractive Index (RI) detector and two PSS SDV 5 µm linear M columns. HPLC grade THF was used as the eluent, at 1.0 mL/min, at 30 °C. Monodisperse polystyrene standards were used for calibration.

Phosphorus End-Group Tests. Polymer samples (40 mg) were dissolved in $CDCl_3$ (0.5 mL) and a solution (40 µL) containing $Cr(acac)_3$ (5.5 mg) and internal standard, bisphenol A (400 mg) in pyridine (10 mL), followed by 40 µL of 2-chloro-4,4,5,5-tetramethyl dioxaphospholane.(*1*)

Differential Scanning Calorimetry (DSC). Recorded for purified polymer samples of were measured using a DSC25 (TA Instruments). A sealed, empty crucible was used as a reference, and the DSC was calibrated using sapphire and indium. Samples were heated from -80 °C to 150 °C, at a rate of 10 °C min^{-1}, under N_2 flow (80 mL min^{-1}), followed by a 5-minute isotherm at 200 °C, to erase thermal history. Samples were subsequently cooled to -80 °C, at a rate of 10 °C min^{-1}, and kept at -80 °C for a further 5 minutes, followed by a heating-cooling procedure from -80 °C to 200 °C, at a rate of 10 °C min^{-1}. Each sample was analysed over two heating-cooling cycles. Glass transition temperatures (T_g) are reported as the midpoint of the transition taken from the second heating cycle.

Thermogravimetric Analysis (TGA). Measured using a TGA5500 system (TA Instruments). Samples were heated from 40 °C to 600 °C, at a rate of 5 °C min^{-1}, under N_2 flow (100 cm^3 min^{-1}).

Film Preparation. Transparent films were prepared by solvent casting into Teflon moulds from THF. Films were dried in a vacuum oven for at least 48 hours prior to use. Samples were then compression moulded using a Carver mini CH CE Press (5420CE.4040C00) with heated plates and hydraulic compression press. Polymers were placed between two metal sheets lined with Teflon, heated to 140 °C, 5 min, under 1 ton m^{-2}.

Fourier-Transform Infrared Spectroscopy (FTIR). Spectra were obtained on a Shimadzu IRSpirit spectrometer, fitted with a KBr window and DLATGS detector with temperature control. FT-IR spectra were recorded inside a glove box using a single reflection ATR accessory and measured in transmission scanning mode. Solid polymer films were scanned from 4700-340 cm^{-1} (100 scans, 4 cm^{-1} resolution).

Tensile Testing. Tests were carried out using an Instron 8600 series universal testing system, using a 50 N load cell and 250 N pneumatic grips. Dumbbell-shaped specimens were cut using a Zwick ZCP020 cutting press, equipped with a cutting device for ISO 527-2 type 5B. Uniaxial extension experiments (10 mm min^{-1} cross-head speed) were run according to ISO 527. Pre-stretching cycle was conducted for all materials prior to cyclic tensile testing.

Dynamic Mechanical Analysis (DMA). Thermal analysis was carried out using a DMA850 (TA Instruments), using an ACS III cooling system. Specimens of uniform width (5.3 mm) were cut using two parallel blades. For oscillatory temperature ramps, samples were heated from -80 °C to 250 °C (or until the material deformed beyond the limits of the geometry employed), at a rate of 3 °C min^{-1}, with a frequency of 1 Hz, and 0.1 % strain amplitude. For time-temperature superposition master curves, samples were cooled from 0 to -80 °C, with frequency sweeps from 0.1 to 100 Hz, collected at 10 °C intervals (0.1 % strain amplitude). Humidity testing was conducted using DMA-RH accessory (2% RH min^{-1} humidity ramp rate).

Rheology. Creep-recovery experiments were conducted on an ARES-G2 (TA Instruments) using polymer samples between 8 mm stainless steel plates. Stress control conditioning experiments were conducted at 30 °C in the linear viscoelastic region as determined by amplitude sweeps. 5 kPa of stress was applied to the samples for 100 s followed by a period of 0 kPa for 100 s, and the stain (creep) exhibited monitored, this was repeated a further 3 times.

Small-angle X-ray Scattering (SAXS). Polymer films (prepared as for mechanical testing as described above) were submitted to Harwell Diamond Light Source in a solid sample grid for SAXS analysis (DL-SAXS, P38 instrument). Scans (3 × 5 min) were conducted at camera lengths of 4.5 and 1 m, beam energy = 9.2 keV (using the Ga MetalJet). SAXS data reported are an average of the 3 scans measured from data collected at 1 m. Samples were not annealed prior to testing to reflect the experimental conditions of tensile testing. 2D scattering patterns were reduced to 1D using Dawn software developed at the Diamond Light Source. (*2, 3*)

Solid-State PCHC Depolymerization. The chemical recycling experiments were conducted in a TGA5500 system and samples were loaded under air (TA Instruments).

Laboratory-Scale Chemical Recycling. Under an N_2 atmosphere **P1**-Zn (0.76 g), a round bottom flask. A distillation glassware apparatus (short-path) was attached to it, with the collection flask being cooled. The apparatus was placed under vacuum (~20 mbar) and the Schlenk tube heated to 20 °C for 24 h, after which atmospheric pressure was re-established in the system using N_2. A mixture mixture of ε-DL, *trans*-CHC, CPO and CHO (77 % yield) was collected.

Reagents and Methods

The macrocyclic ligand, H_2L, was synthesized following a previously reported procedure.[2] Magnesium bis(1,1,1,3,3,3-hexamethyldisilazan-2-ide) (97%) and bis(pentafluorophenyl)zinc (97%) were purchased from Sigma-Aldrich. Bis(pentafluorophenyl)zinc was used as received and magnesium bis(1,1,1,3,3,3-hexamethyldisilazan-2-ide) was recrystallized from hexane. Solvents used for synthesis and polymerization were collected from a solvent purification system (SPS), degassed with three freeze-pump-thaw cycles, and stored over 4 Å molecular sieves, under an inert atmosphere. Cyclohexene 1,2-epoxide (98 %) (CHO) was purchased from Alfa Aesar, dried by stirring over CaH_2, followed by fractional distillation at 60 °C. Cyclopentene 1,2-epoxide (98 %) (CPO) was purchased from Sigma-Aldrich, dried by stirring over CaH_2, followed by fractional distillation at 60 °C. ε-Decalactone (99%) (ε-DL) was dried over CaH_2, followed by fractional distillation at 115 °C under reduced pressure, and kept under a nitrogen atmosphere. δ-Jasmolactone (95%) (δ-JL) was was dried over CaH_2, followed by fractional distillation at 120 °C under reduced pressure, and kept under a nitrogen atmosphere. 1,4-Benzenedimethanol (BDM) was recrystallized from toluene three times and stored under inert atmosphere. Research-grade carbon dioxide was dried through a Drierite column and two additional drying columns (Micro Torr, Model number: MC1-804FV) in series before use. 3-Mercaptopropionic acid was purchased from Sigma-Aldrich and degassed by bubbling through nitrogen. Diethly zinc and triethyl aluminium were purchased from Sigma-Aldrich and used as received.

Synthesis of [LZnMg(C_6F_5)$_2$] Catalyst(*4*)

The catalyst was synthesized following a previously reported procedure.**(*4*)** Under inert conditions, the macrocyclic ligand (H_2L) (0.50 g, 0.90 mmol) and recrystallised $Mg\{N[Si(CH_3)_3]_2\}_2$ (0.31 g, 0.90 mmol) were dissolved in THF (10 mL), at 25 °C, for 1 hour. $Zn(C_6F_5)_2$ (0.36 g, 0.90 mmol) was dissolved in THF (5 mL) and added dropwise to the reaction mixture. The dark orange solution was stirred overnight, at 25 °C. The pale orange reaction mixture was then cooled to -29 °C and the supernatant solvent was removed. The solid product was washed (hexane) and the product (precipitate) dried under reduced pressure. Yield: 0.54 g (61 %). ^1H NMR (400 MHz, $CDCl_3$) δ 6.81 (s, 2H), 6.76 (s, 2H), 4.42 – 4.25 (m, 4H), 3.40 (d, J = 13.7 Hz, 2H), 3.26 (d, J = 13.3 Hz, 2H), 3.14 – 2.94 (m, 4H), 2.68 (s, 6H), 2.11 – 2.00 (m, 2H), 1.27 (s, 3H), 1.20 (s, 18H), 1.17 (s, 3H), 1.06 – 1.01 (m, 6H).

General Procedure for P1-vinyl Synthesis

[LZnMg(C_6F_5)$_2$] (80 mg, 0.08 mmol), 1,4-benzenedimethanol (22.8 mg, 0.16 mmol), cyclohexene oxide (2.7 mL, 27 mmol), cyclopentene oxide (2.4 mL, 27 mmol), δ-jasmolactone (1.1 mL, 6.4 mmol) and ε-decalactone (10.6 mL, 59 mmol) were dissolved in toluene (11 mL). The solution was stirred at room temperature for 3 h. The solution then diluted with a additional toluene (28 mL) and added into a high-pressure Steel reactor which was placed under 20 bar of CO_2 (using a triple manifold line), heated to 80 °C and stirred for 96 h. The reaction mixture was quenched by exposed to air and the polymer was precipitated in methanol (3x) and dried under reduced pressure. Yield: 12.0 g (71%).

General Procedure for Carboxylic Acid Functionalization

P1-vinyl (9 g, 3.6 mmol PJL), 3-mercaptopropionic acid (1.2 mL, 14 mmol) and 2,2-dimethoxy-2-phenylacetophenone (364 mg, 1.4 mmol) was dissolved in THF (90 mL). The reaction mixture was stirred at room temperature and irradiated with UV light (365 nm) for 60 min. The reaction mixture was quenched by exposure to air, precipitated in methanol twice and dried under reduced pressure. Yield: 8.6 g (92%).

General Procedure for Ionomer Formation

P1-COOH (1.5 g, 0.60 mmol of PJL_{COOH}) was dissolved in dry THF (30 mL) and a $ZnEt_2$ THF solution (37 mg in 5 mL, 0.30 mmol) was slowly added dropwise to the polymer solution. The reaction mixture was stirred at room temperature for 1 h and solvent cast.

General Procedure for reP1-vinyl Synthesis

[LZnMg(C_6F_5)$_2$] (20 mg, 0.02 mmol), 1,4-benzenedimethanol (5.7 mg, 0.04 mmol), cyclohexene oxide (0.65 mL, 6.55 mmol virgin), cyclopentene oxide (0.58 mL, 6.55 mmol virgin), the chemically recycled mixture of CHO and CPO (0.03 mL, 0.41 mmol), δ-jasmolactone (0.29 mL, 1.6 mmol) and ε-decalactone (0.37 mL, 2.66 mmol of chemically recycled and 2.17 mL, 12.10 mmol virgin) were dissolved in toluene (2.75 mL). The solution was stirred at room temperature for 3 h. The solution then diluted with additional toluene (28 mL) and added into a high-pressure Steel reactor which was placed under 20 bar of CO_2 (using a triple manifold line), heated to 80 °C and stirred for 96 h. The reaction mixture was quenched by exposed to air and the polymer was precipitated in methanol (3x) and dried under reduced pressure. Yield: 2.65 g (63%).

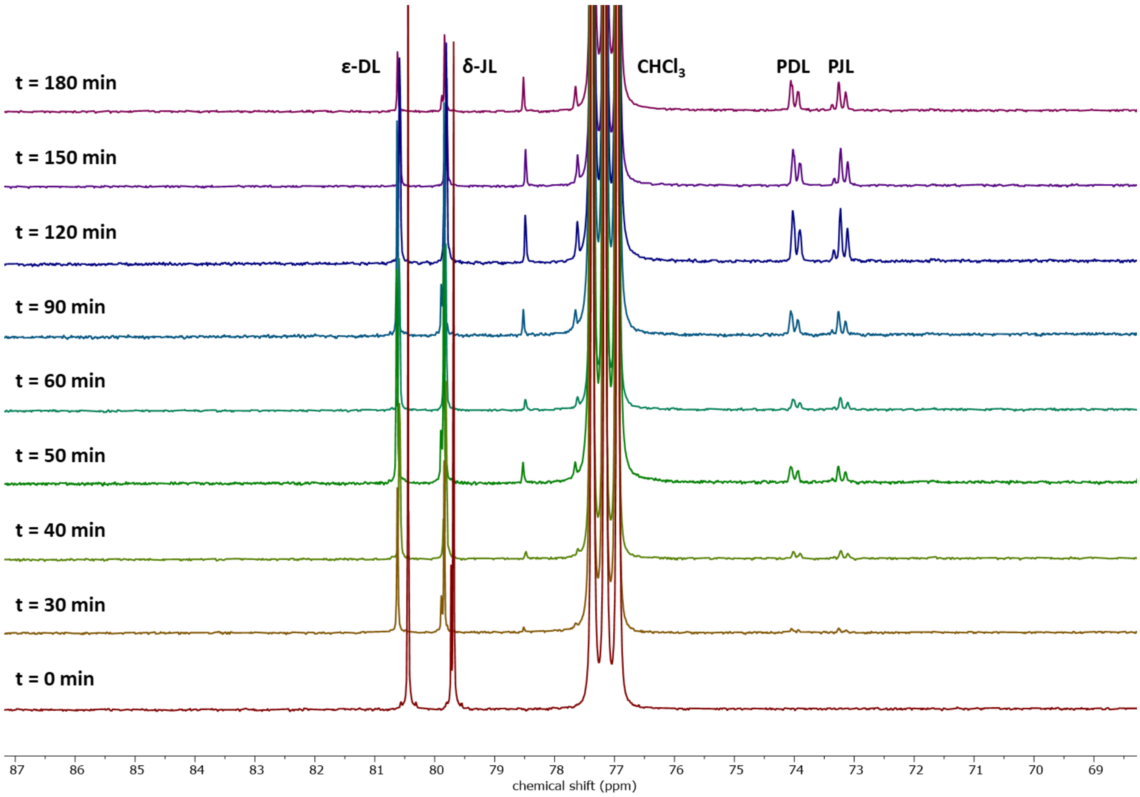

Figure S1: Quantitative $^{13}C\{^{1}H\}$ NMR (150 MHz, $CDCl_3$) spectra of ε-DL/δ-JL copolymerization aliquots (Diethyl carbonate used as an internal standard).

Meyer-Lowry Model(*5*): Compositional drift in f_{DL} fitted to toal conversion χ_n

$$\chi_n = 1 - \left(\frac{f_1}{f_{10}}\right)^{\alpha} \left(\frac{f_2}{f_{20}}\right)^{\beta} \left(\frac{f_1}{f_{10}}\right)^{\alpha} \left(\frac{f_{10} - \delta}{f_1 - \delta}\right)^{\gamma}$$

$$\alpha = \frac{r_2}{(1 - r_2)} \; ; \; \beta = \frac{r_1}{(1 - r_1)} \; ; \; \gamma = \frac{1 - r_1 r_2}{(1 - r_1)} \; ; \; \delta = \frac{1 - r_2}{(2 - r_1 - r_2)}$$

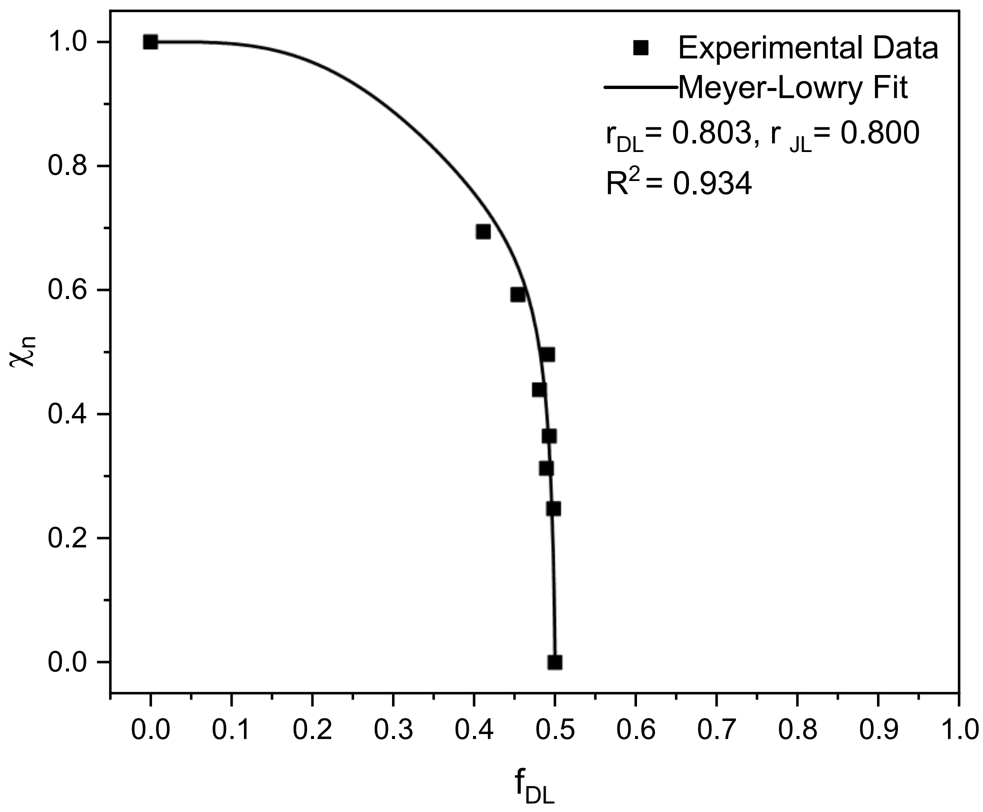

Figure S2: Meyer-Lowry fit to ε-DL/δ-JL copolymerization data.

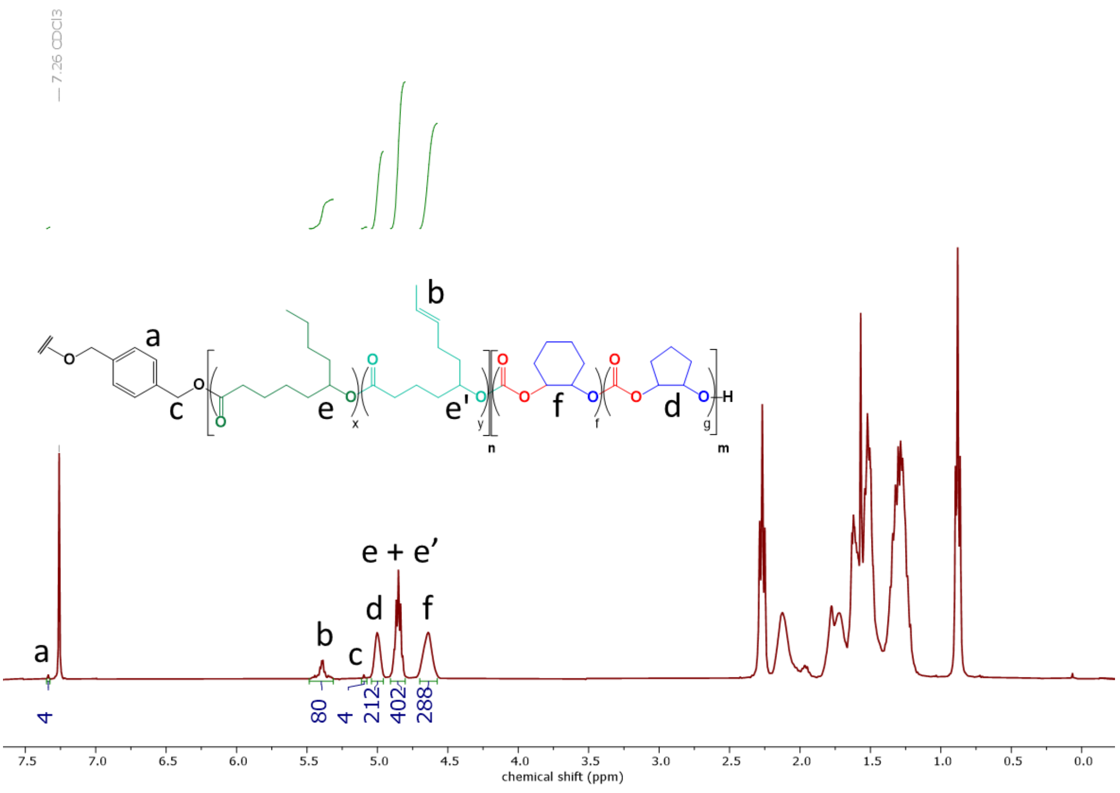

Figure S3: ^{1}H NMR (400 MHz, $CDCl_3$) spectrum of **P1**-vinyl.

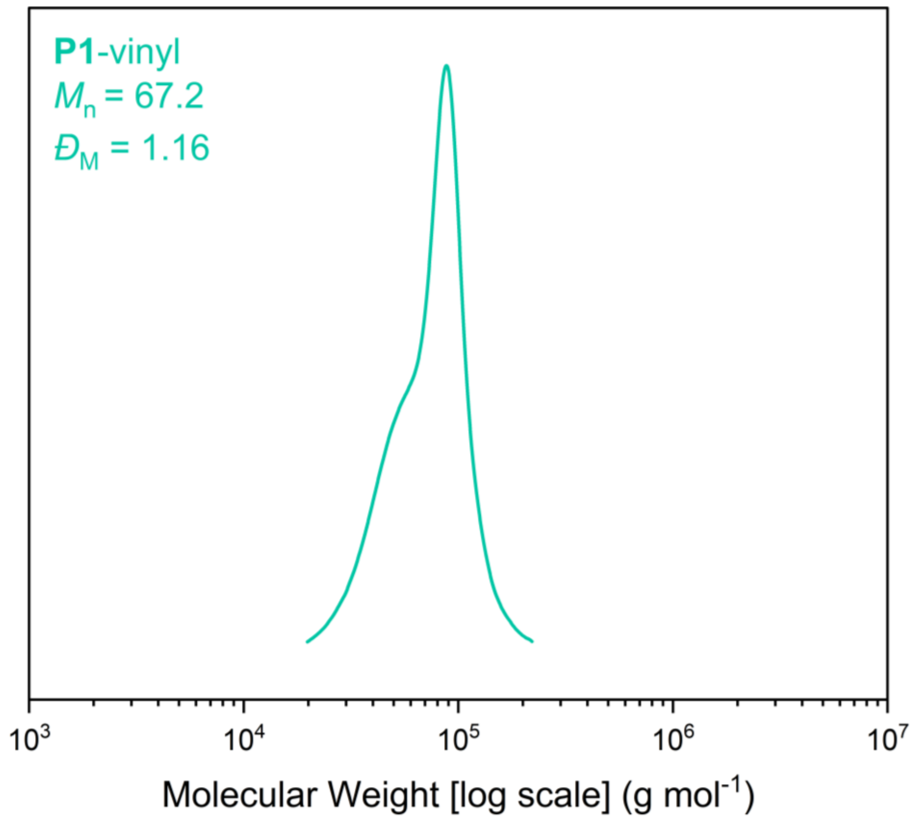

Figure S4: SEC (THF, 1 mL min^{-1}) traces for **P1**-vinyl. The instrument is calibrated with poly(styrene) standards.

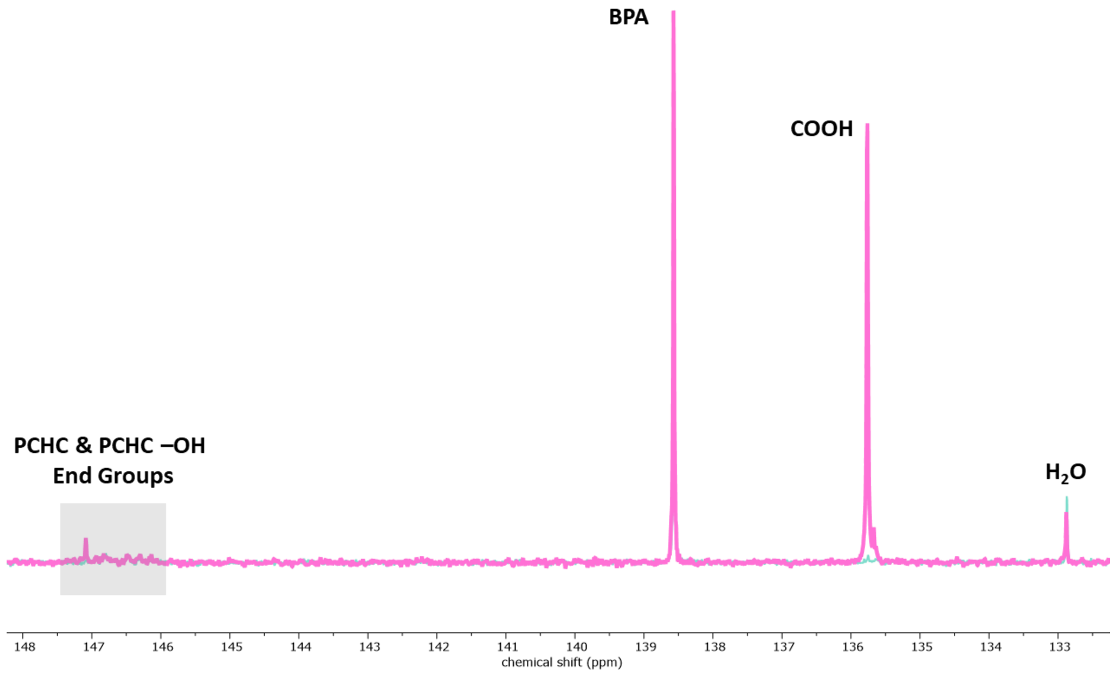

Figure S5: $^{31}P\{^1H\}$ NMR end-group analysis of **P1**-vinyl (teal) and **P1**-COOH (pink).

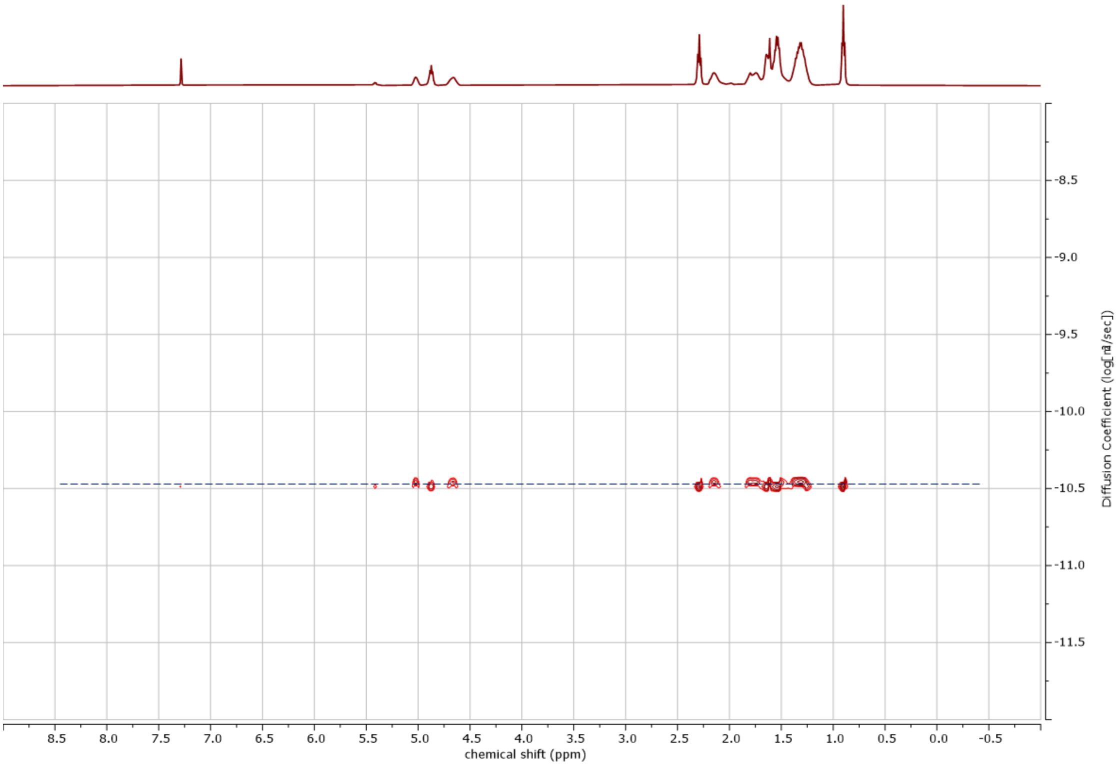

Figure S6: ^{1}H DOSY NMR (400 MHz, $CDCl_3$) spectrum of **P1**-vinyl.

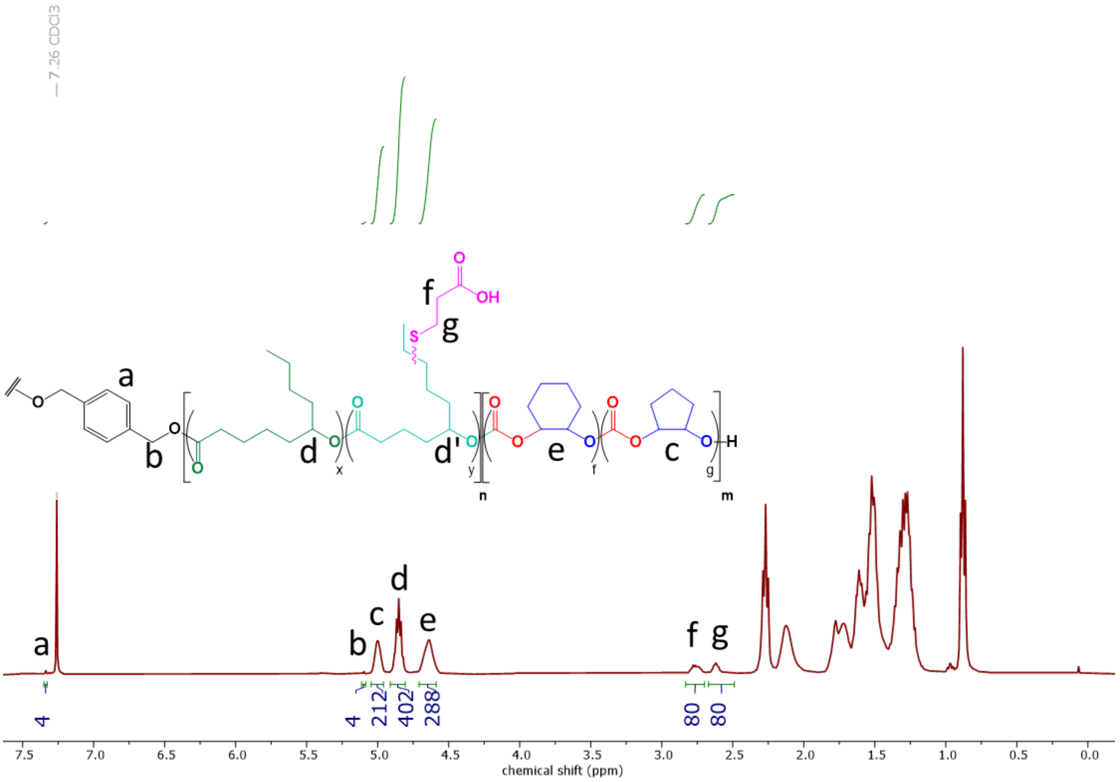

Figure S7: ^{1}H NMR (400 MHz, $CDCl_3$) spectrum of **P1**-COOH.

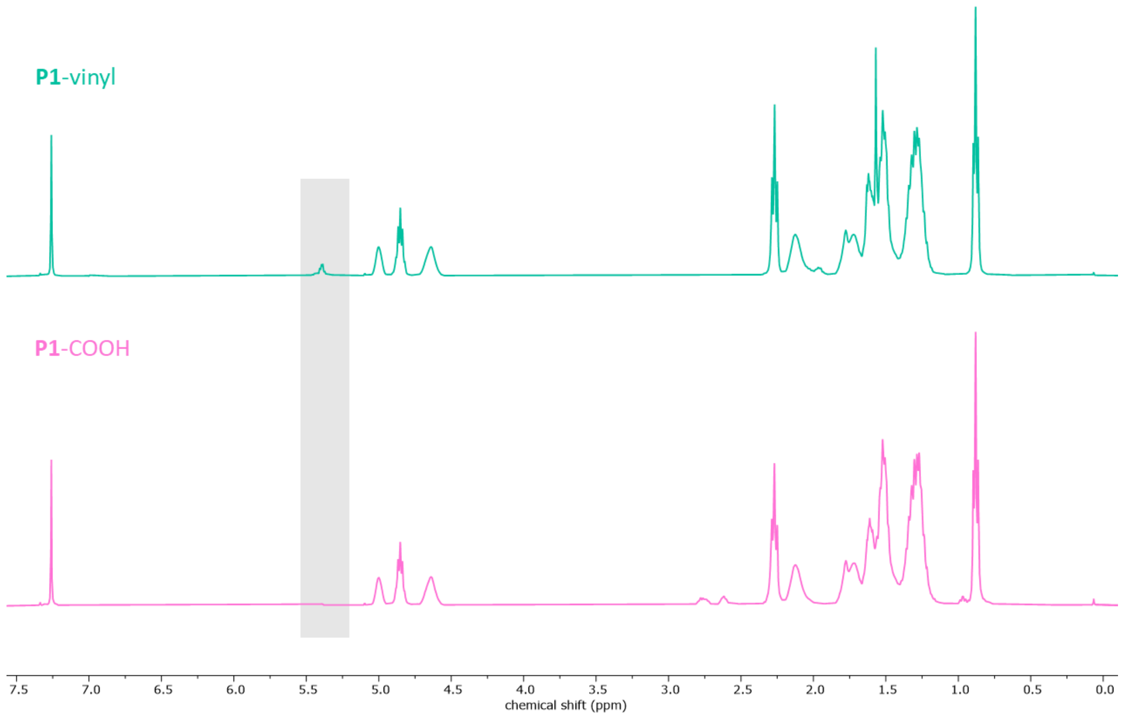

Figure S8: ^{1}H NMR (400 MHz, $CDCl_3$) spectra of **P1**-vinyl and **P1**-COOH highlighting loss of vinyl signals after post-polymerization functionalization.

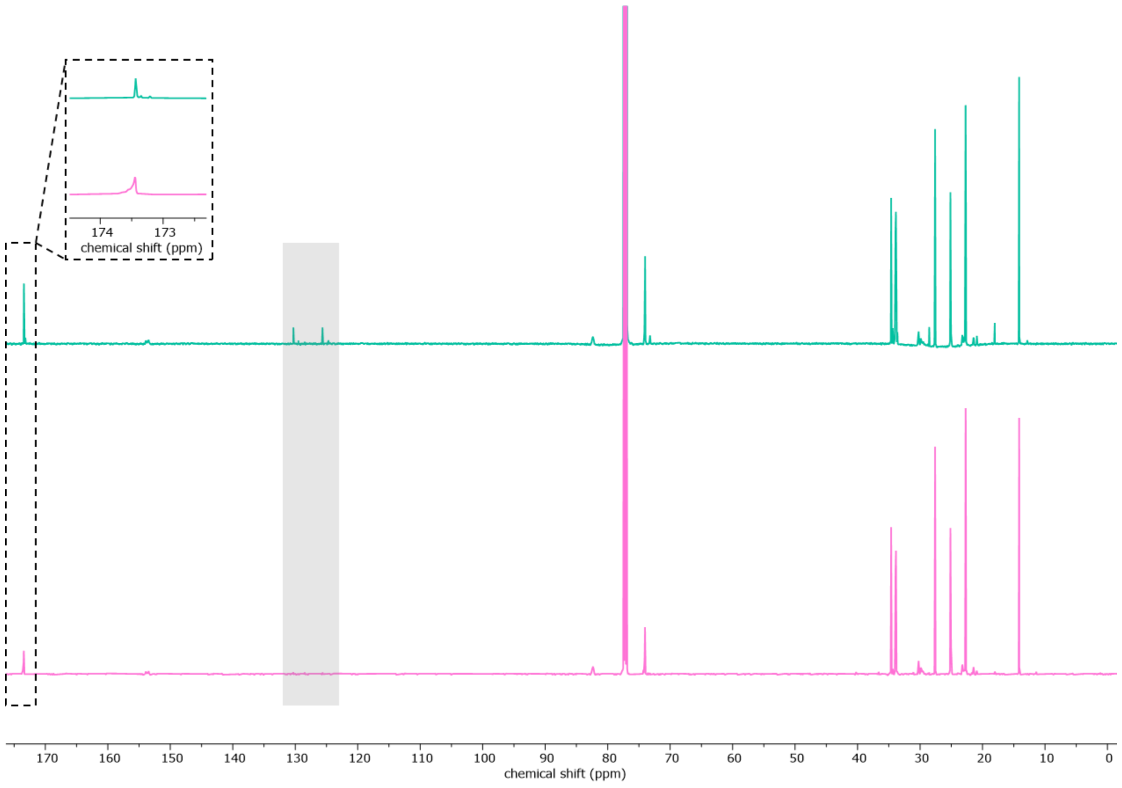

Figure S9: $^{13}C\{^{1}H\}$ NMR (150 MHz, $CDCl_3$) spectra of **P1**-vinyl and **P1**-COOH highlighting loss of vinyl signals after post-polymerization functionalization and broadening of carbonyl signal.

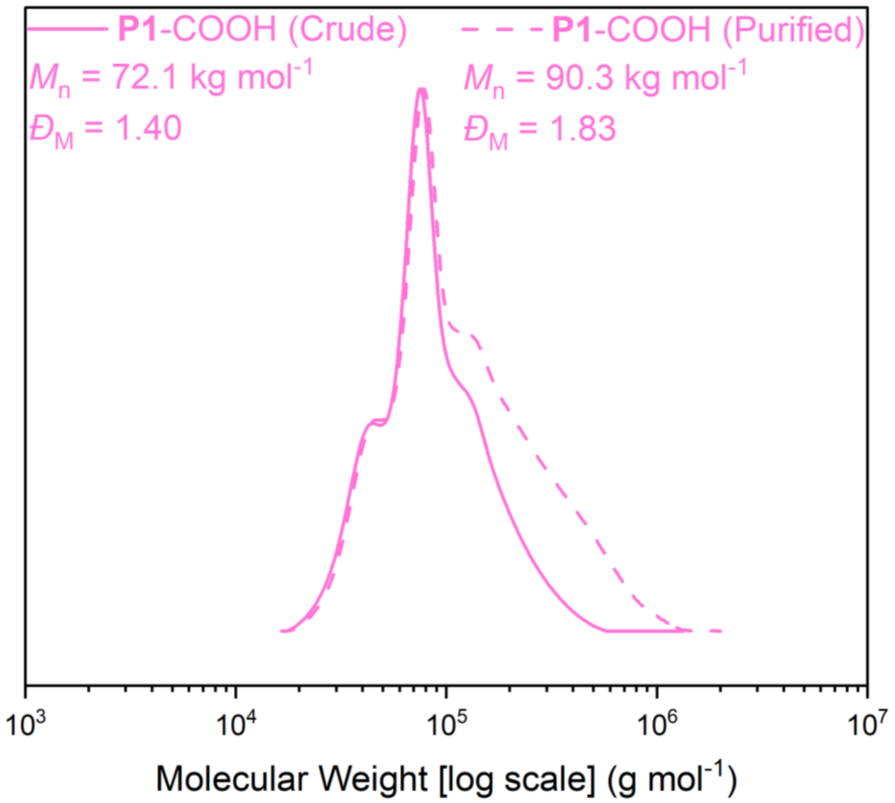

Figure S10: SEC (THF, 1 mL min^{-1}) traces for **P1**-COOH: a post reaction aliquot (solid) and following precipitation and drying (dashed). The instrument is calibrated with poly(styrene) standards.

The SEC traces of crude and purified P1-COOH highlight two contributions to the broadened distribution. The low-molecular-weight shoulder arises from chains initiated by trace ring-opened JL hydroxy acid present in the δ-JL feedstock, producing slightly lower molar mass triblocks for **P1**-vinyl and **P1**-COOH. The emergence and growth of the high-molecular-weight shoulder upon purification (M_n increasing from 72.1 to 90.3 kg mol^{-1} and $Đ_M$ broadening from 1.40 to 1.83) are attributed to enhanced aggregation of COOH-functionalized chains in the SEC eluent once residual small molecules are removed. These features are consistent with reversible hydrogen-bond–driven association in solution rather than structural defects, as confirmed by full thiol-ene functionalization in ^{1}H NMR and the absence of crosslinking or changes in solubility.

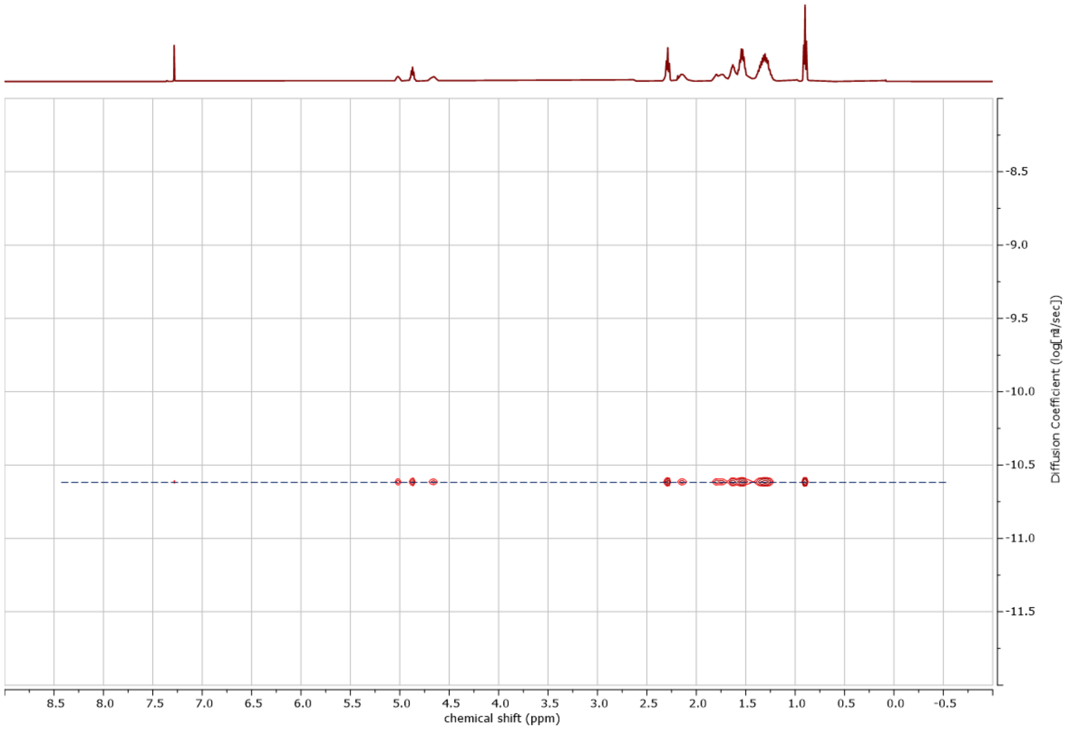

Figure S11: ^{1}H DOSY NMR (400 MHz, $CDCl_3$) spectrum of **P1**-vinyl.

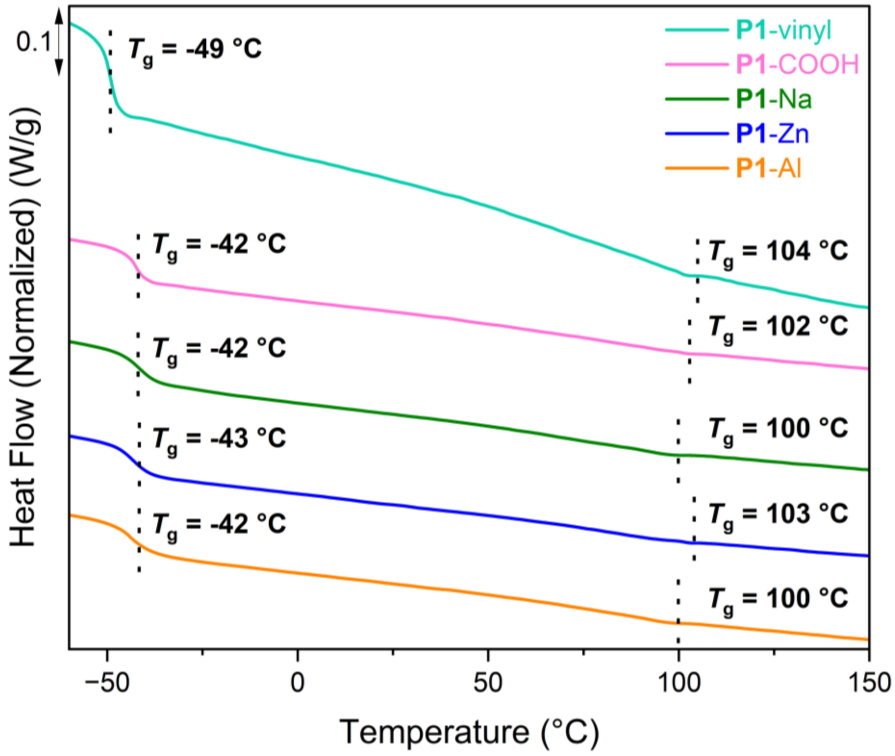

Figure S12: Differential Scanning Calorimetry (DSC) traces of **P1**-vinyl, **P1**-COOH., **P1**-Na, **P1**-Zn, and **P1**-Al.

Table S1: Summary of lower $T_{g,DMA}$ values

Material	$T_{g,DMA}$ (°C)
**P1**-vinyl	-50
**P1**-COOH	-45
**P1**-Na	-41
**P1**-Zn	-44
**P1**-Al	-36

$T_{g,DMA}$ values taken from low temperature peak in loss modulus (G”).

Table S2: Summary of SAXS Data.

Material	q*/Å^{-1}	d/nm	Higher Order Peaks	Assigned Morphology
**P1**-vinyl	0.0278	22.6	√7	HEX
**P1**-COOH	0.0290	21.7	√3, √7	HEX
**P1**-Na	0.0278	22.6	√3, √7	HEX
**P1**-Zn	0.0264	23.8	√3, √7	HEX
**P1**-Al	0.0272	23.1	-	-

Where q* = principle scattering peak (d=2π/q*).

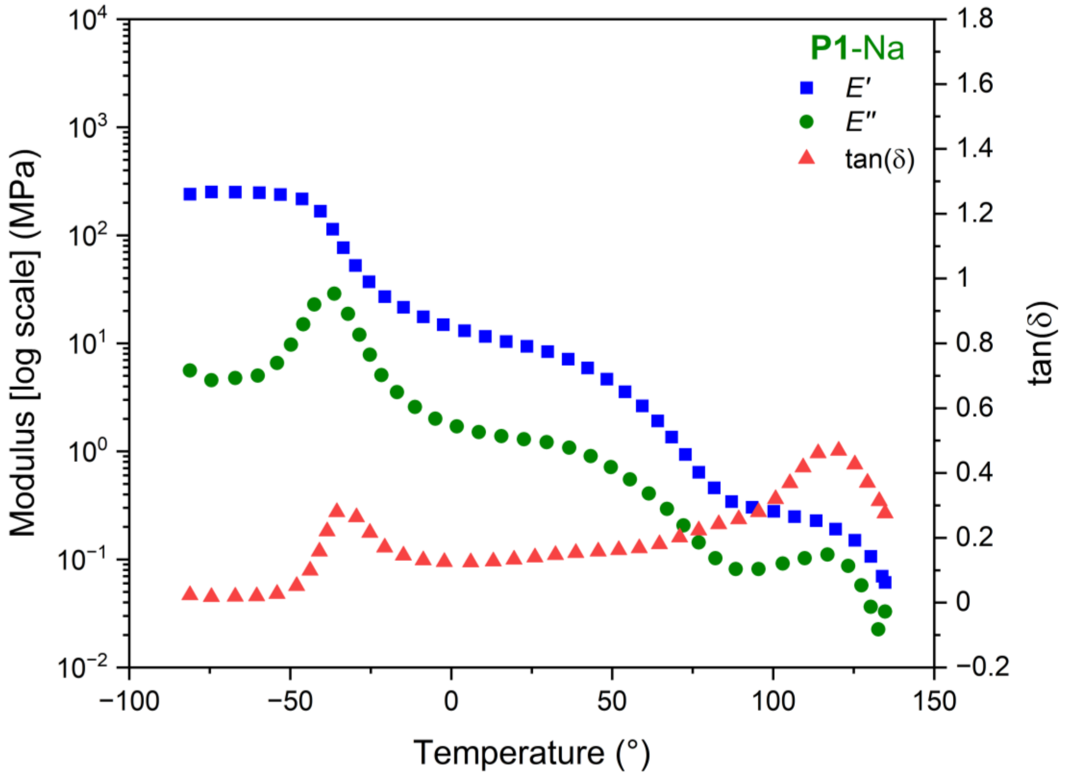

Figure S13: Dynamic mechanical analysis temperature sweeps (0.1% strain, 1 Hz, 3˚C min^{-1}) for **P1**-Na.

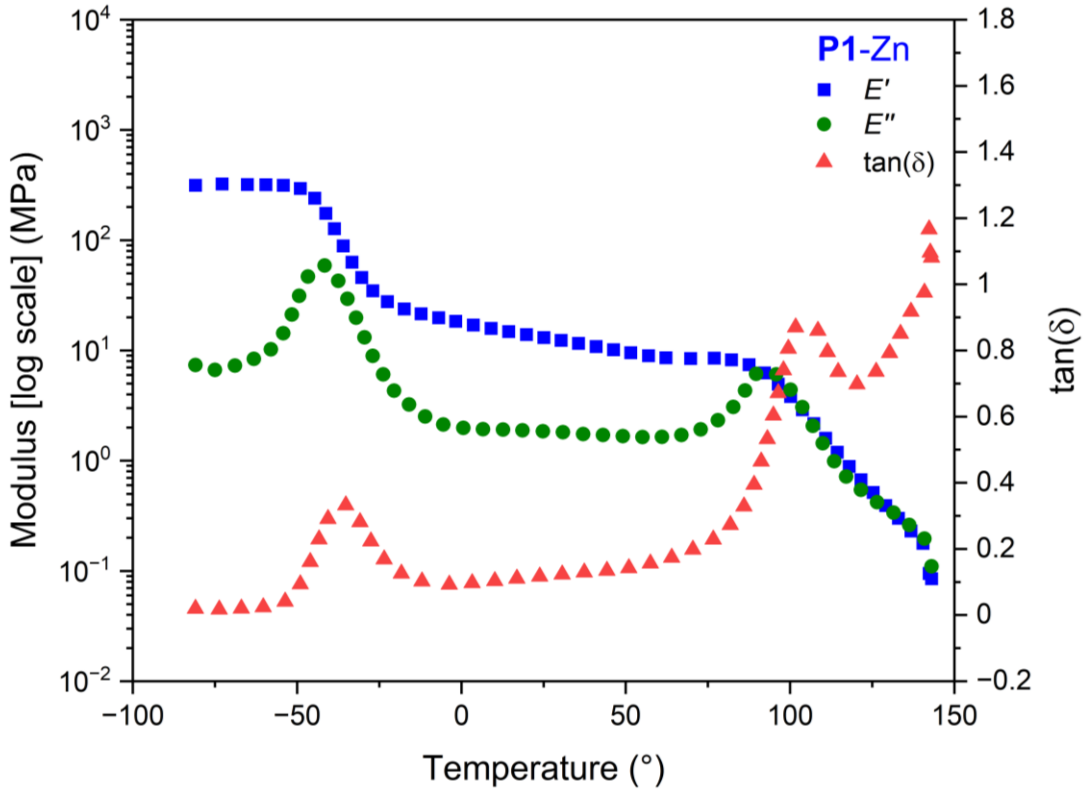

Figure S14: Dynamic mechanical analysis temperature sweeps (0.1% strain, 1 Hz, 3˚C min^{-1}) for **P1**-Zn.

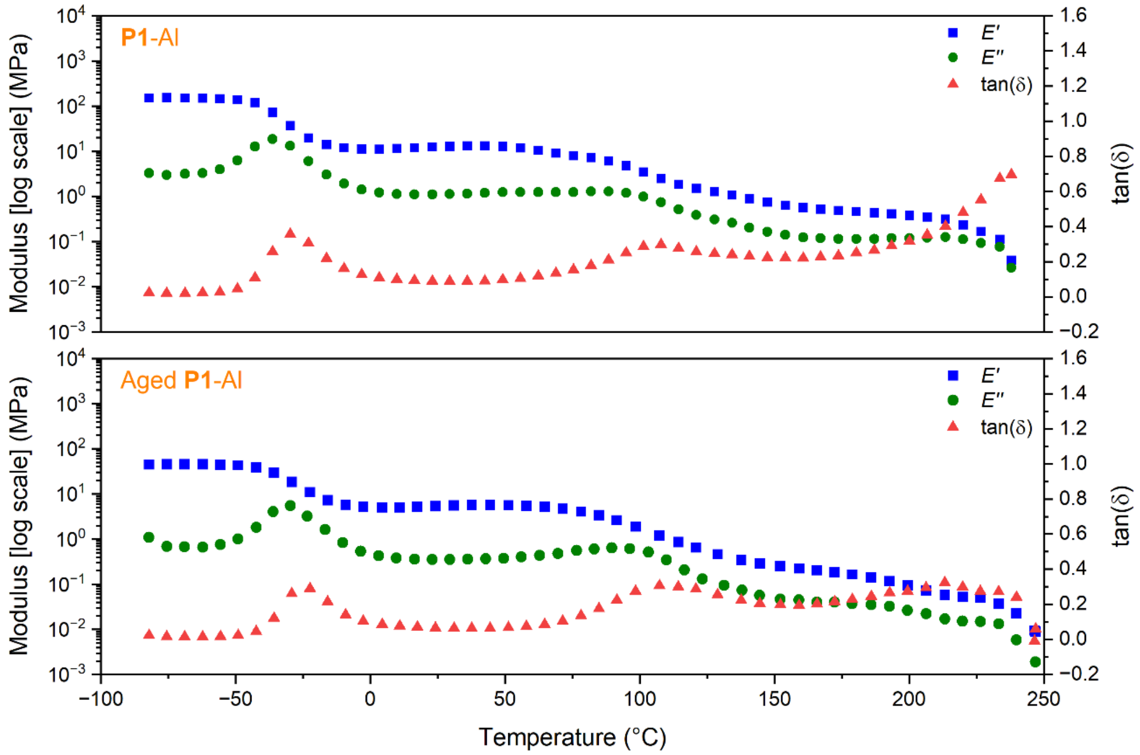

Figure S15: Dynamic mechanical analysis temperature sweeps (0.1% strain, 1 Hz, 3˚C min^{-1}) for virgin **P1**-Al and aged 352 days after mechanical recycling experiments.

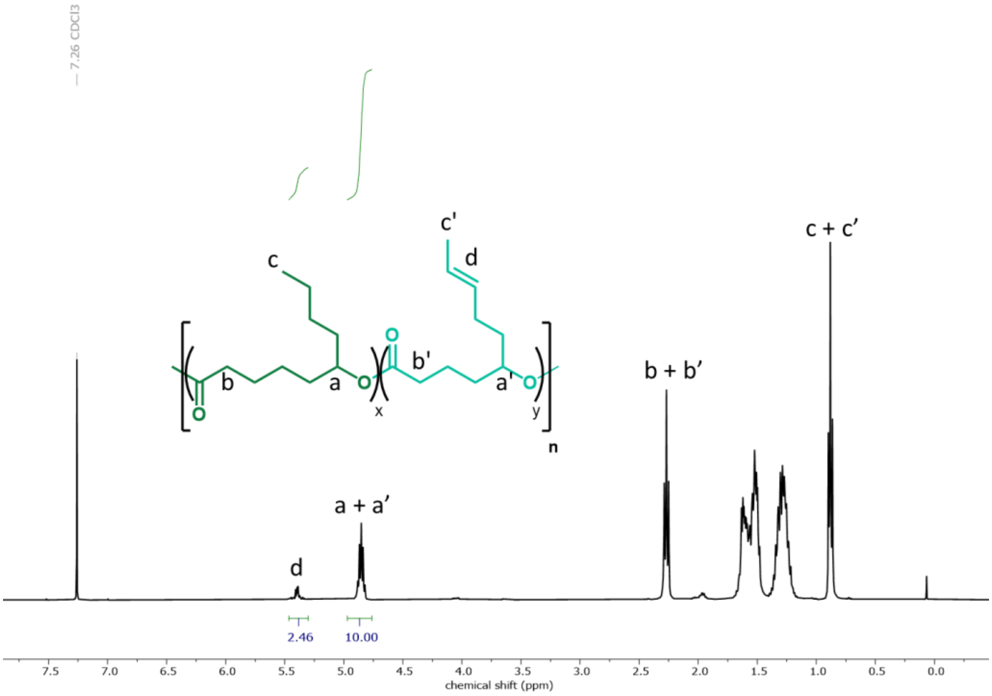

Figure S16: ^{1}H NMR (400 MHz, $CDCl_3$) spectrum of PJL-*co*-PDL.

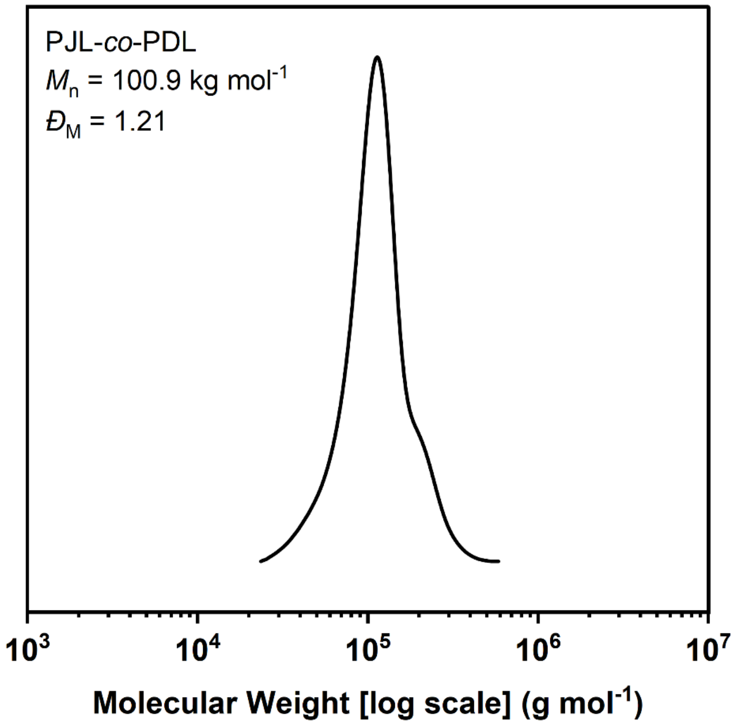

Figure S17: SEC (THF, 1 mL min^{-1}) traces for PJL-*co*-PDL. The instrument is calibrated with poly(styrene) standards.

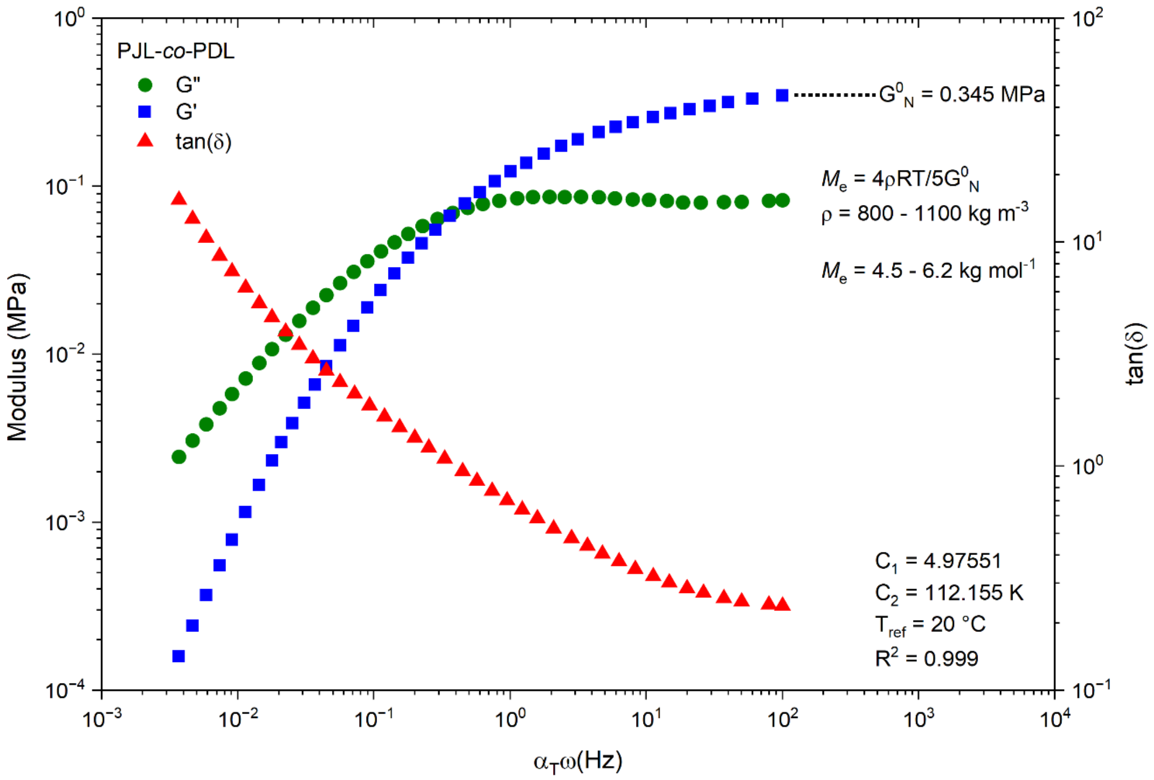

Figure S18: Rheological time-temperature superposition master curve for PJL-*co*-PDL. Melt density assumed to be between 800 and 1100 kg m^{-3}. The effective molecular weight between constraints was estimated by assuming additive contributions from entanglements and dynamic crosslinks $(1/M_{\text{eff}} = 1/M_e + 1/M_{COOAl})$.

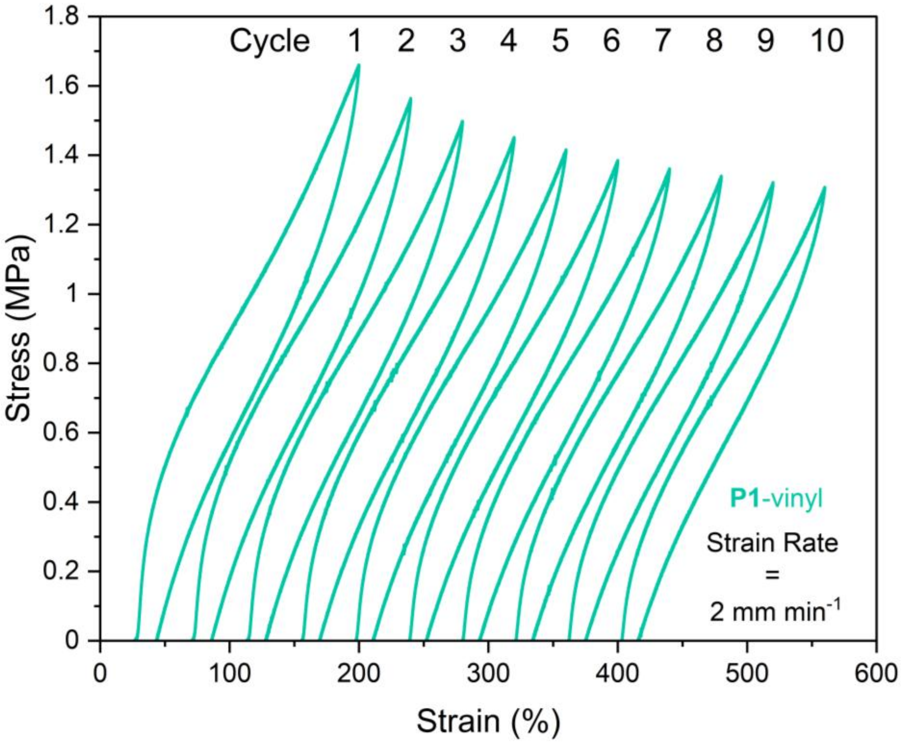

Figure S19: Uniaxial cyclic tensile testing (200% strain, 10 cycles) at 2 mm min^{-1} for **P1**-vinyl.

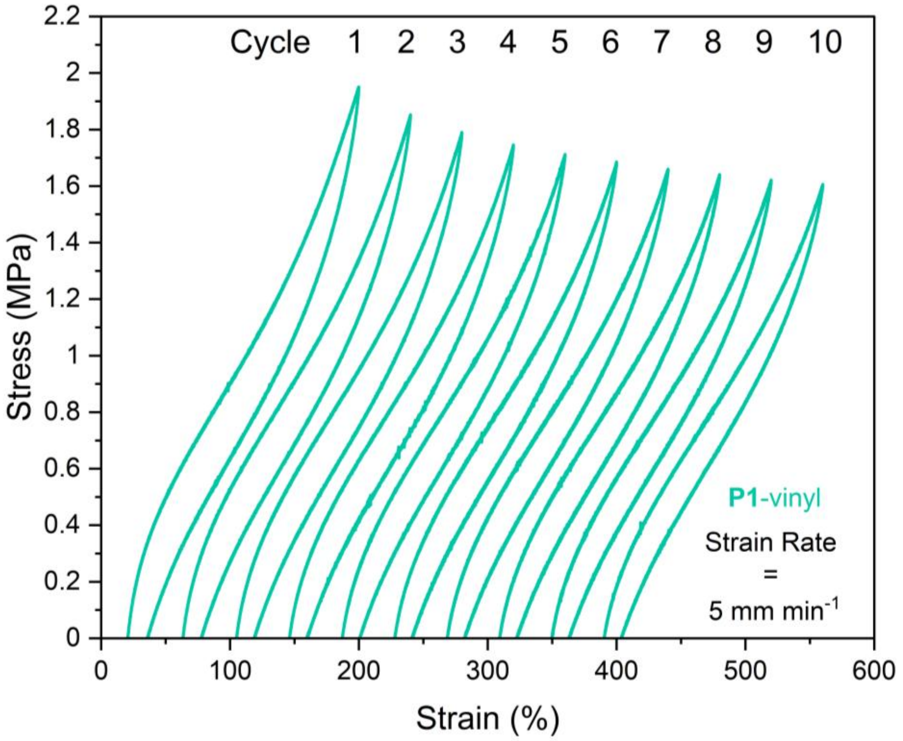

Figure S20: Uniaxial cyclic tensile testing (200% strain, 10 cycles) at 5 mm min^{-1} for **P1**-vinyl.

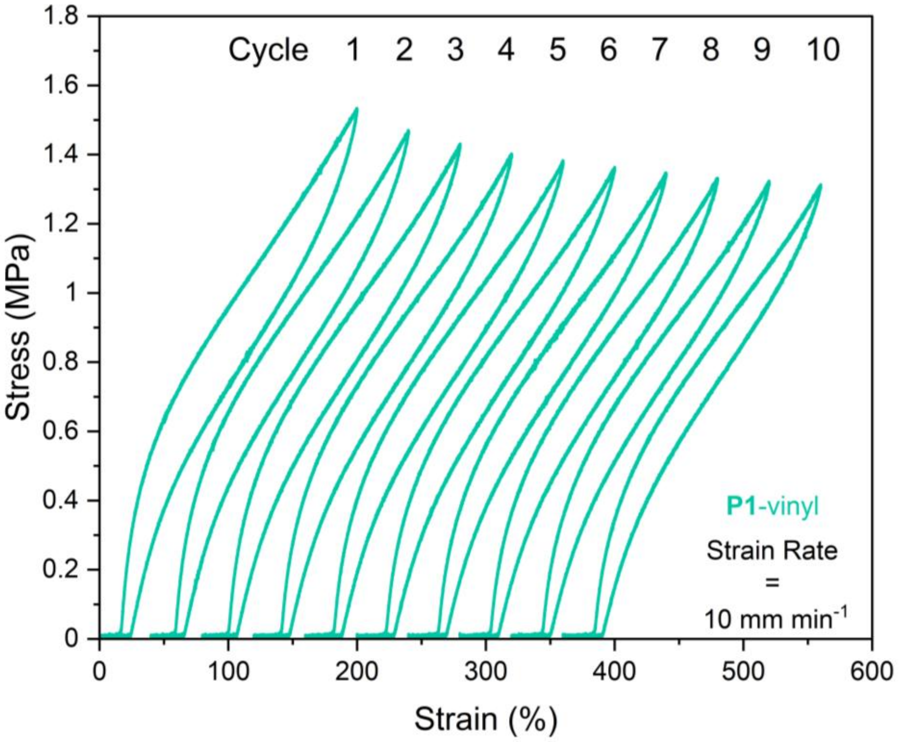

Figure S21: Uniaxial cyclic tensile testing (200% strain, 10 cycles) at 10 mm min^{-1} for **P1**-vinyl.

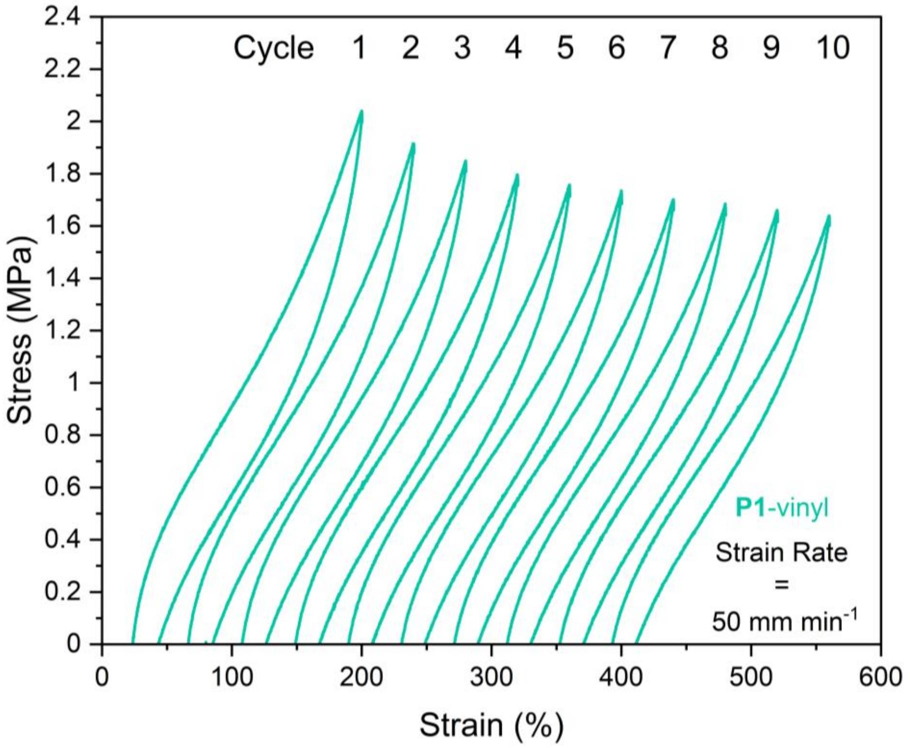

Figure S22: Uniaxial cyclic tensile testing (200% strain, 10 cycles) at 50 mm min^{-1} for **P1**-vinyl.

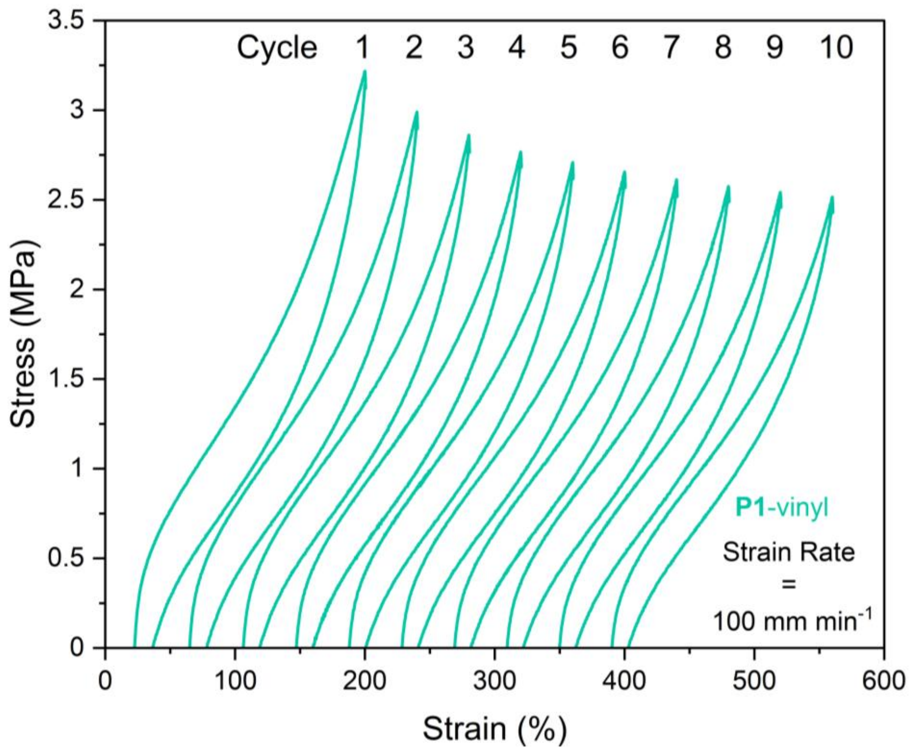

Figure S23: Uniaxial cyclic tensile testing (200% strain, 10 cycles) at 100 mm min^{-1} for **P1**-vinyl.

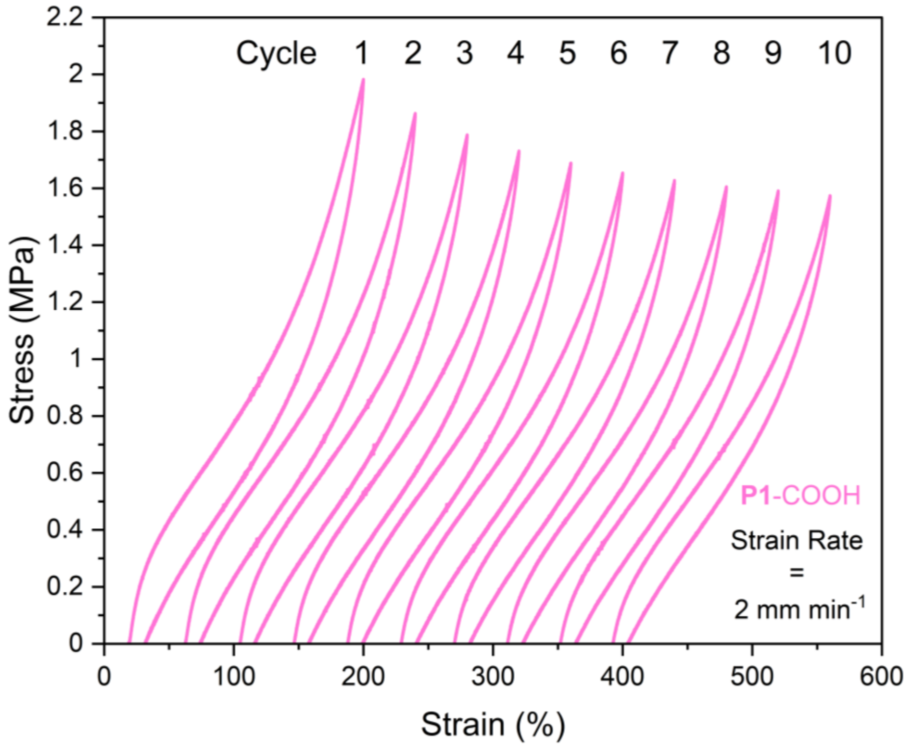

Figure S24: Uniaxial cyclic tensile testing (200% strain, 10 cycles) at 2 mm min^{-1} for **P1**-COOH.

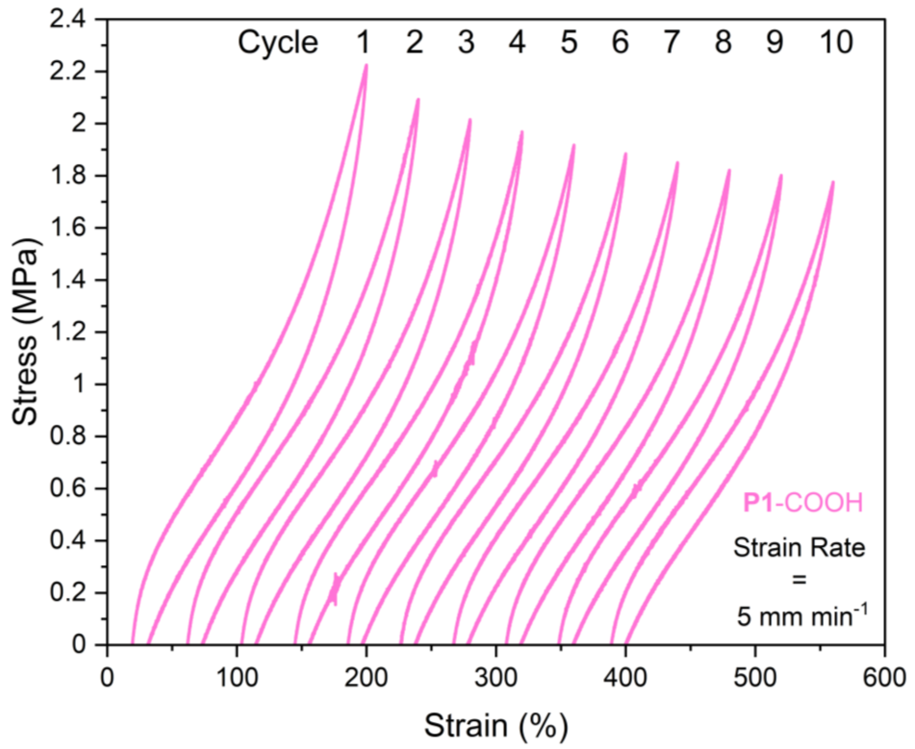

Figure S25: Uniaxial cyclic tensile testing (200% strain, 10 cycles) at 5 mm min^{-1} for **P1**-COOH.

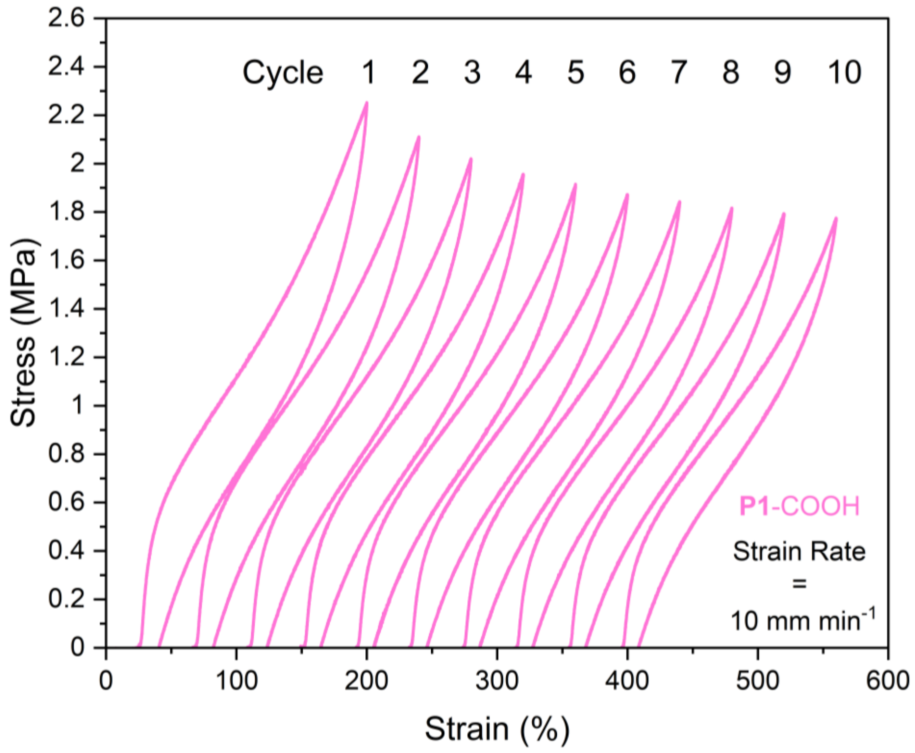

Figure S26: Uniaxial cyclic tensile testing (200% strain, 10 cycles) at 10 mm min^{-1} for **P1**-COOH.

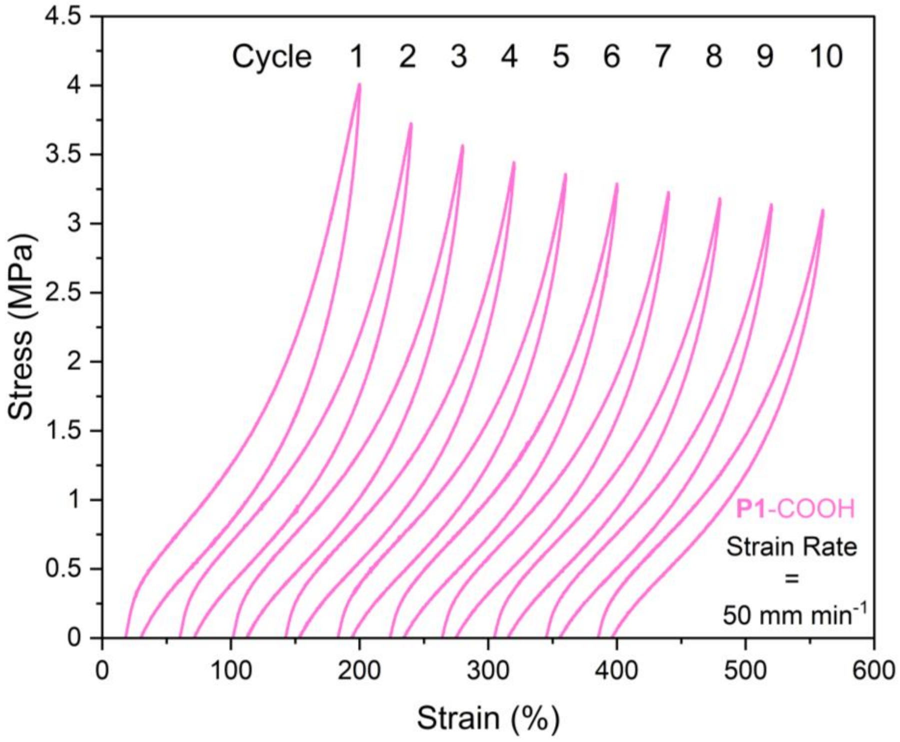

Figure S27: Uniaxial cyclic tensile testing (200% strain, 10 cycles) at 50 mm min^{-1} for **P1**-COOH.

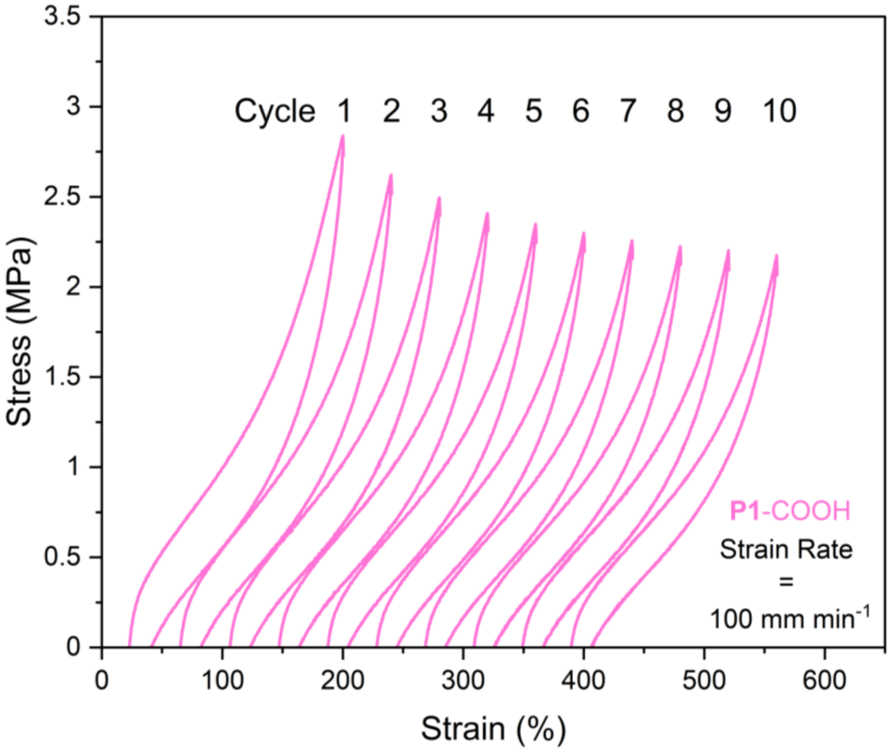

Figure S28: Uniaxial cyclic tensile testing (200% strain, 10 cycles) at 100 mm min^{-1} for **P1**-COOH.

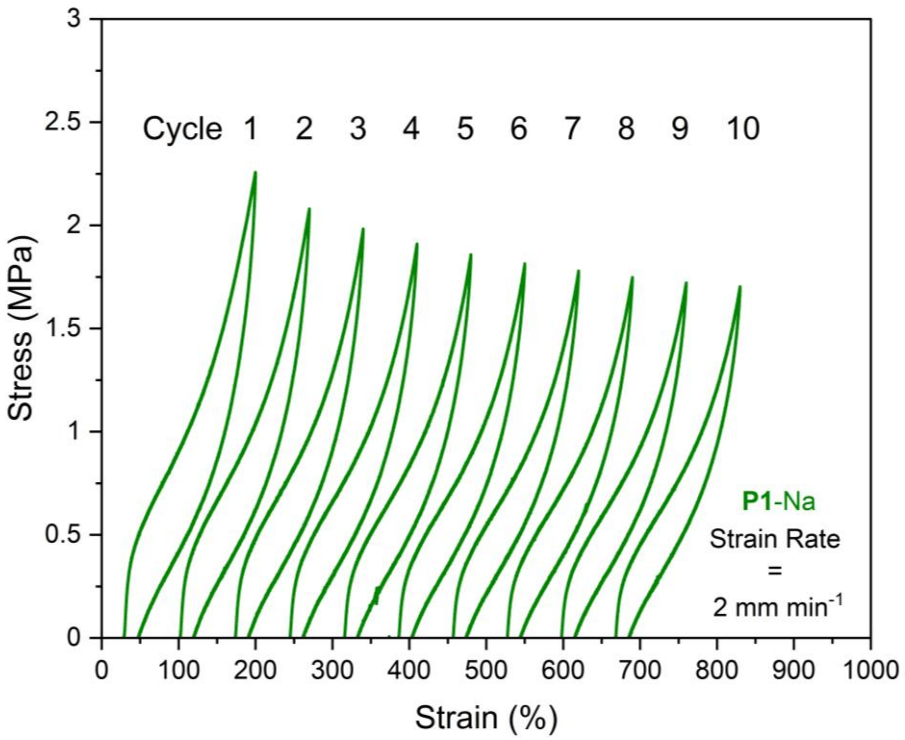

Figure S29: Uniaxial cyclic tensile testing (200% strain, 10 cycles) at 2 mm min^{-1} for **P1**-Na.

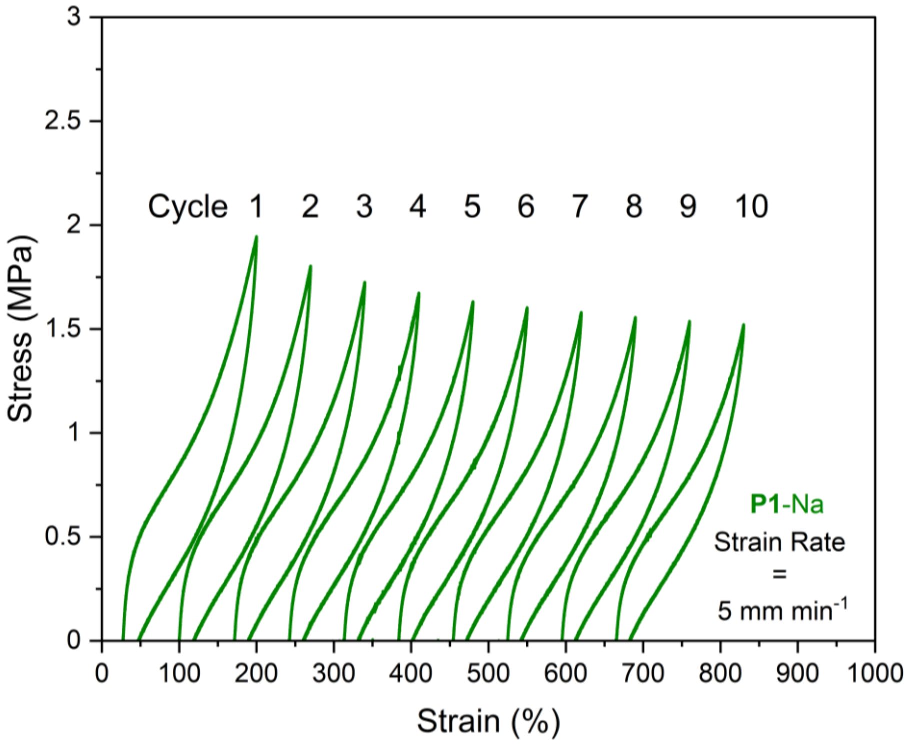

Figure S30: Uniaxial cyclic tensile testing (200% strain, 10 cycles) at 5 mm min^{-1} for **P1**-Na.

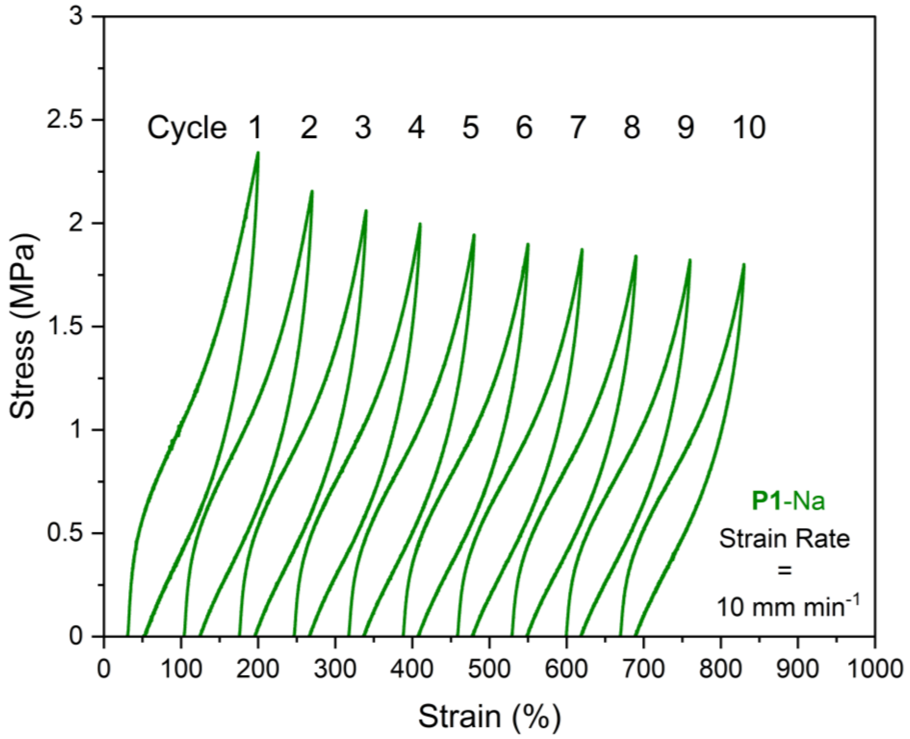

Figure S31: Uniaxial cyclic tensile testing (200% strain, 10 cycles) at 10 mm min^{-1} for **P1**-Na.

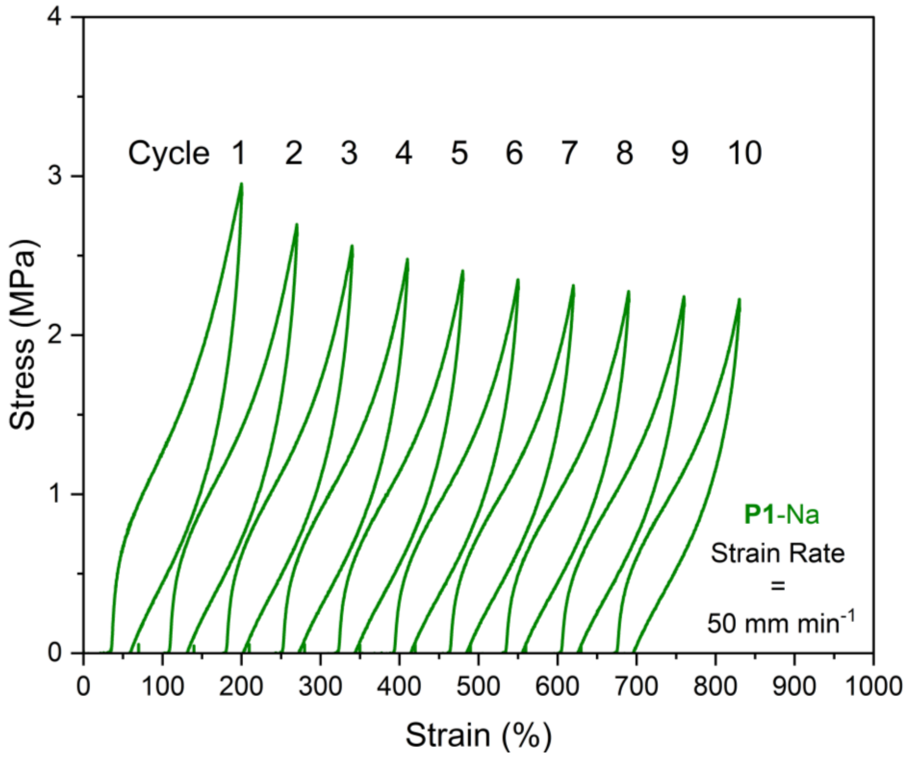

Figure S32: Uniaxial cyclic tensile testing (200% strain, 10 cycles) at 50 mm min^{-1} for **P1**-Na.

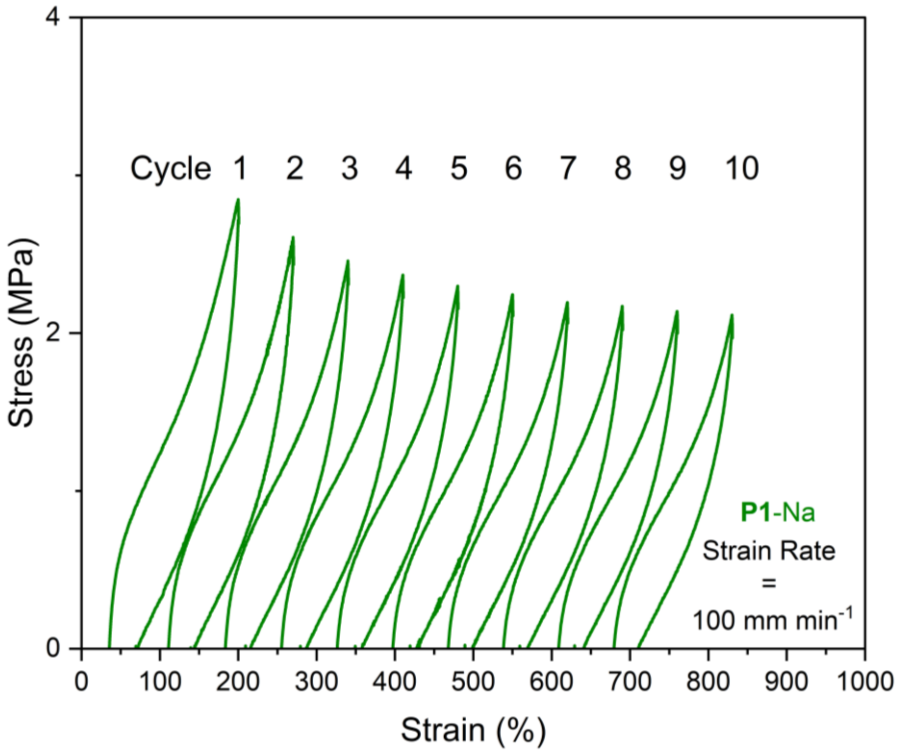

Figure S33: Uniaxial cyclic tensile testing (200% strain, 10 cycles) at 100 mm min^{-1} for **P1**-Na.

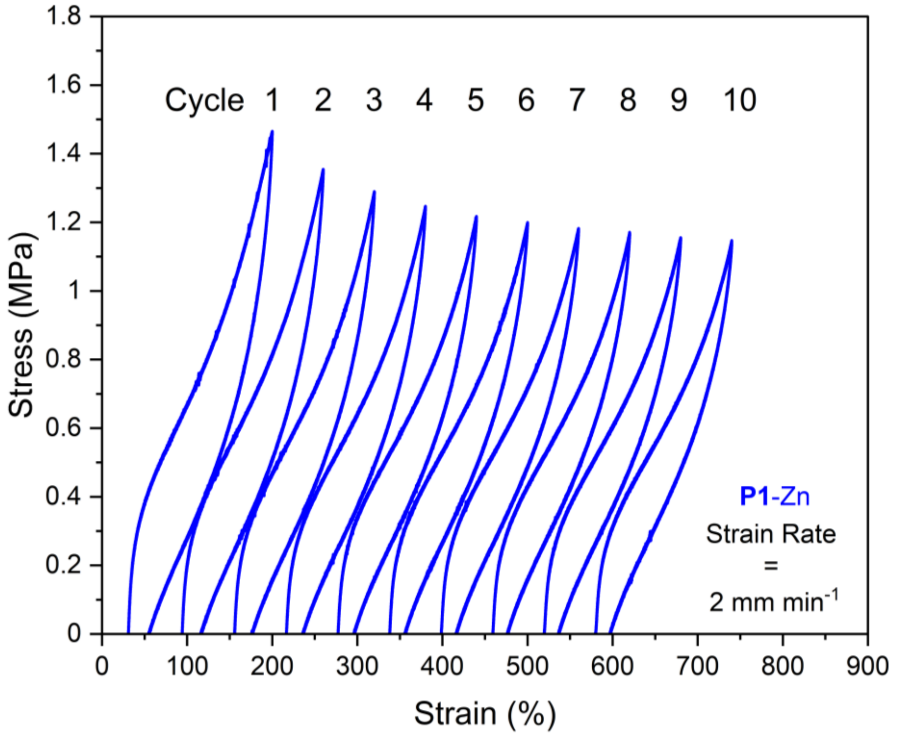

Figure S34: Uniaxial cyclic tensile testing (200% strain, 10 cycles) at 2 mm min^{-1} for **P1**-Zn.

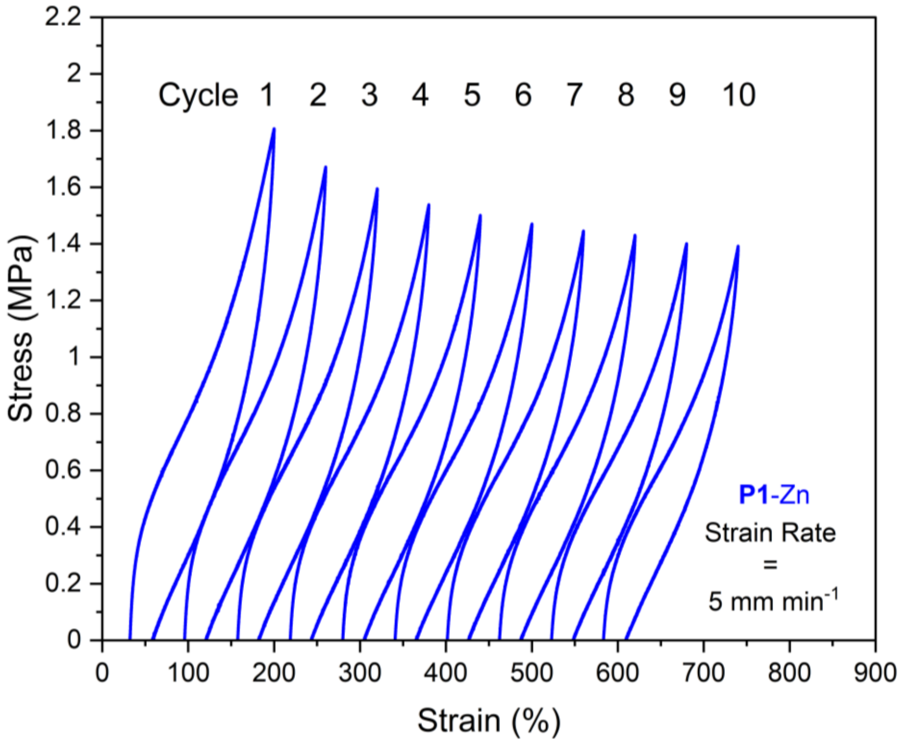

Figure S35: Uniaxial cyclic tensile testing (200% strain, 10 cycles) at 5 mm min^{-1} for **P1**-Zn.

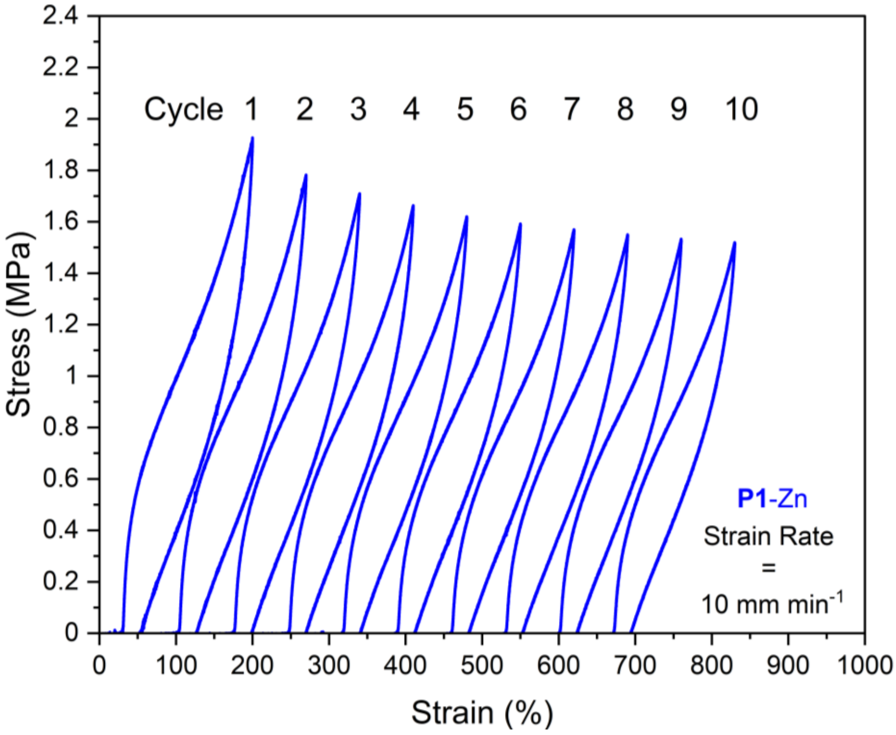

Figure S36: Uniaxial cyclic tensile testing (200% strain, 10 cycles) at 10 mm min^{-1} for **P1**-Zn.

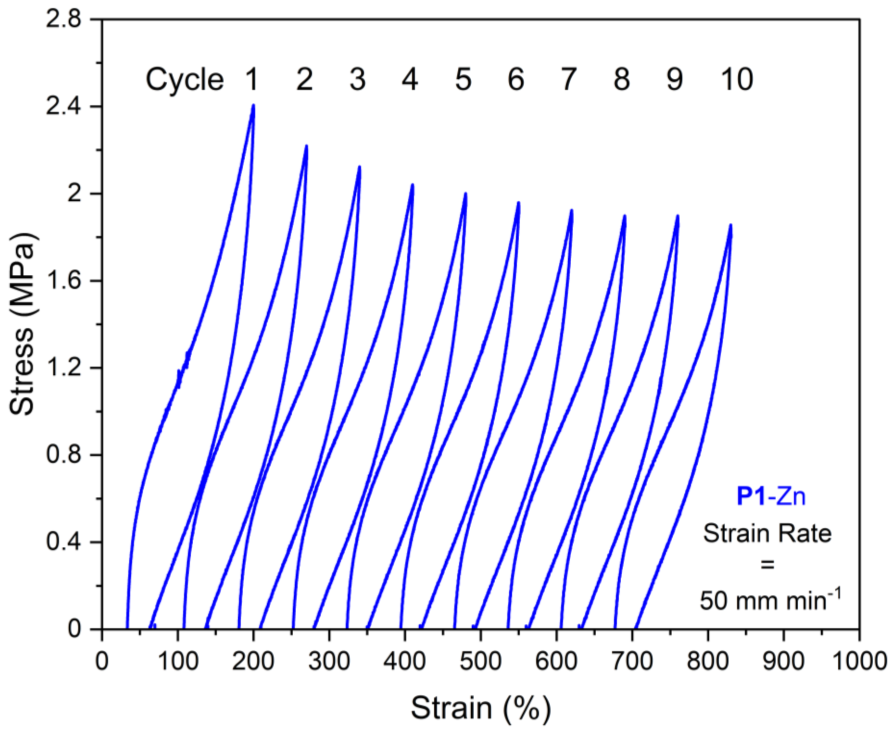

Figure S37: Uniaxial cyclic tensile testing (200% strain, 10 cycles) at 50 mm min^{-1} for **P1**-Zn.

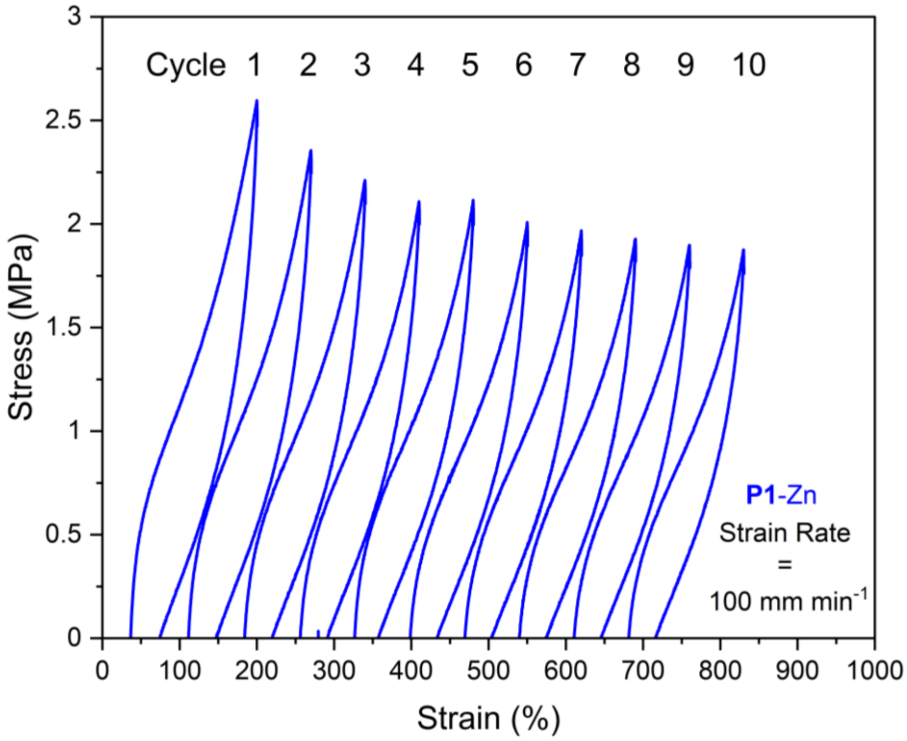

Figure S38: Uniaxial cyclic tensile testing (200% strain, 10 cycles) at 100 mm min^{-1} for **P1**-Zn.

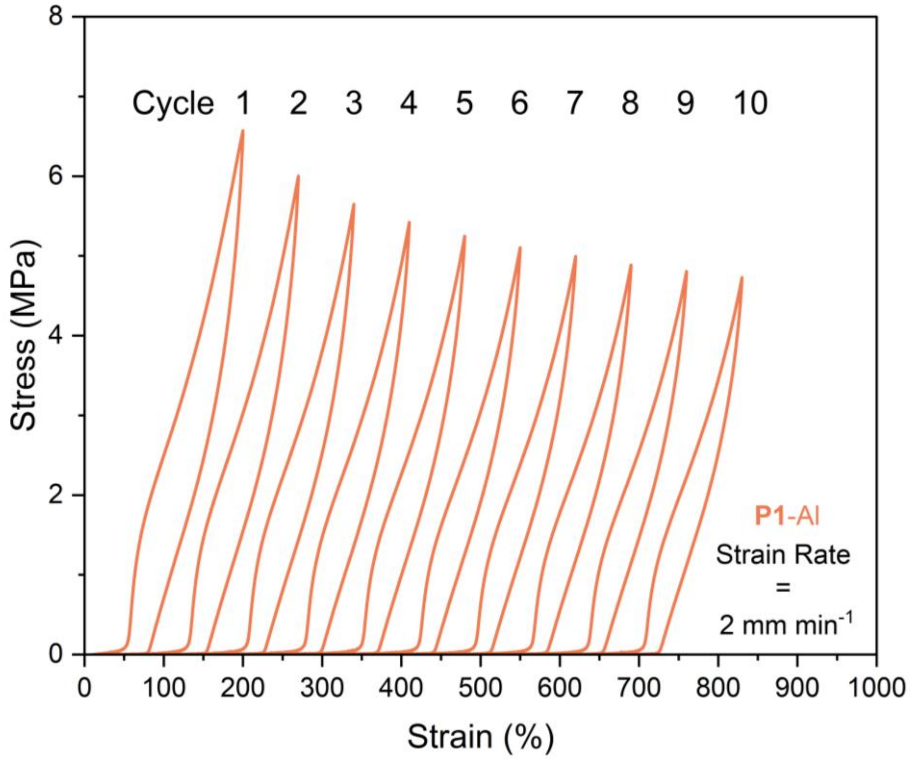

Figure S39: Uniaxial cyclic tensile testing (200% strain, 10 cycles) at 2 mm min^{-1} for **P1**-Al.

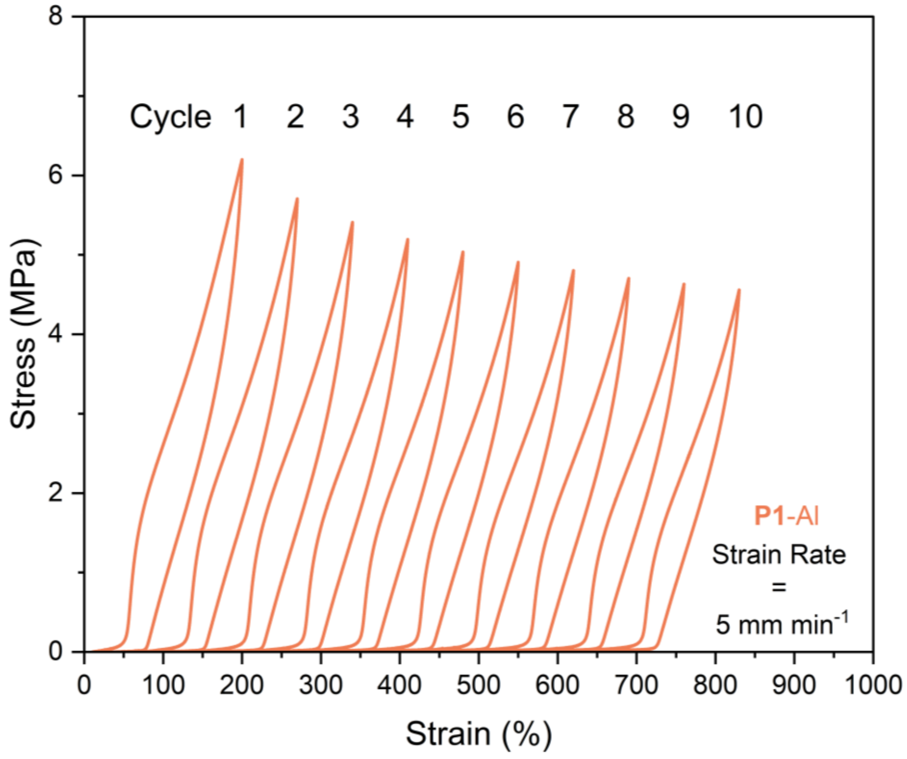

Figure S40: Uniaxial cyclic tensile testing (200% strain, 10 cycles) at 5 mm min^{-1} for **P1**-Al.

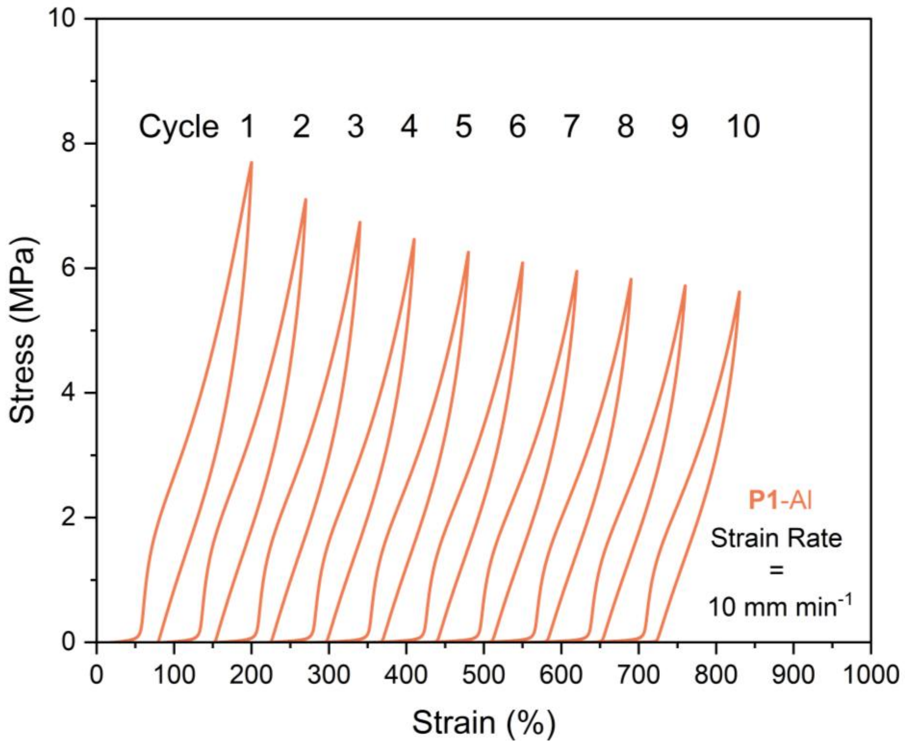

Figure S41: Uniaxial cyclic tensile testing (200% strain, 10 cycles) at 10 mm min^{-1} for **P1**-Al.

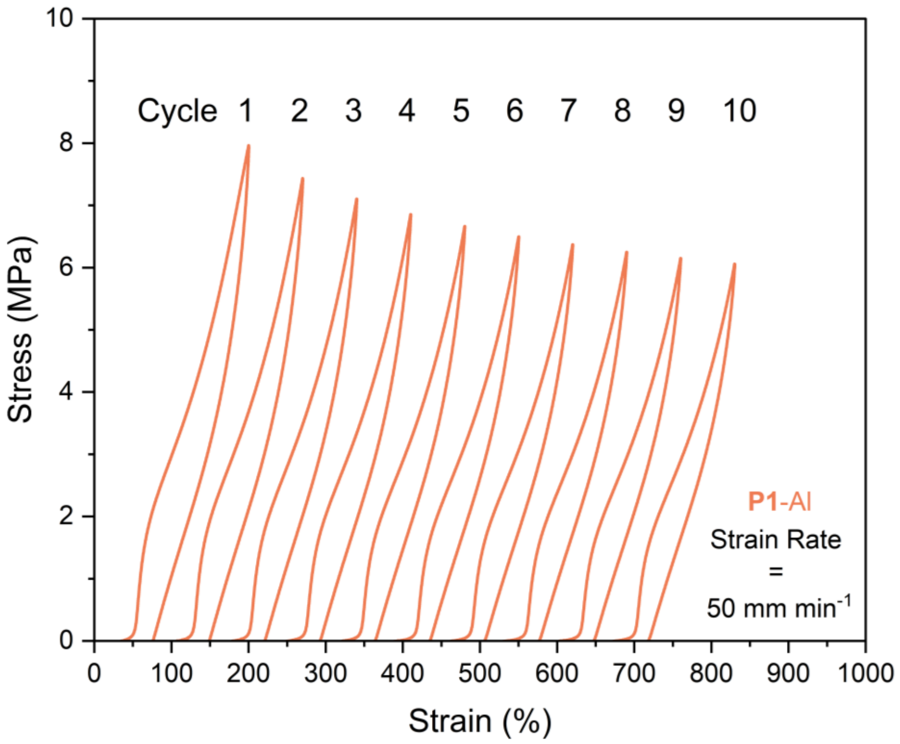

Figure S42: Uniaxial cyclic tensile testing (200% strain, 10 cycles) at 50 mm min^{-1} for **P1**-Al.

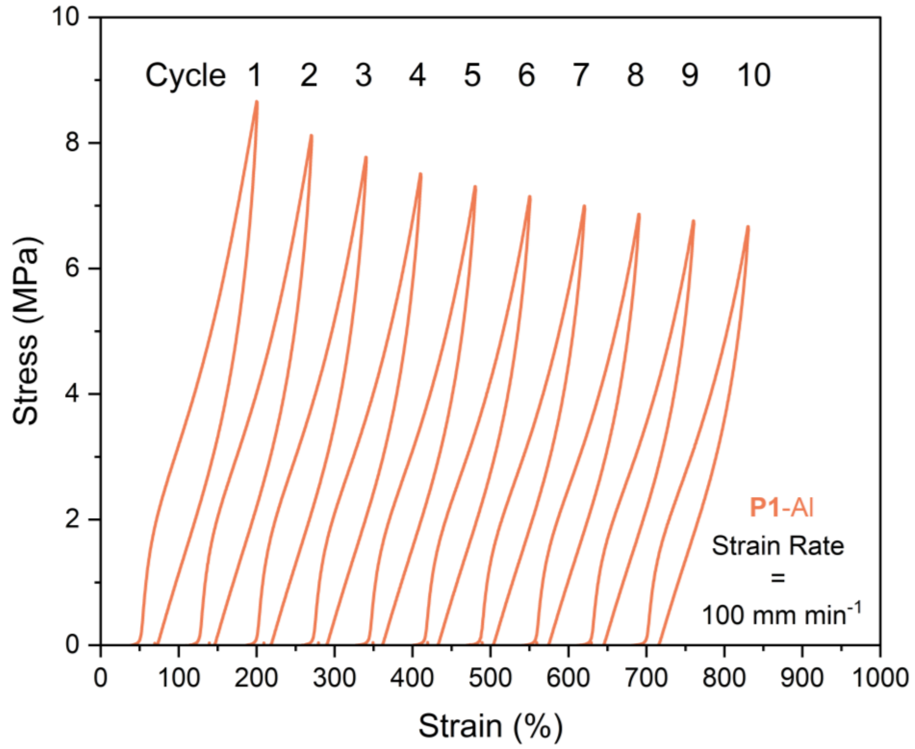

Figure S43: Uniaxial cyclic tensile testing (200% strain, 10 cycles) at 100 mm min^{-1} for **P1**-Al.

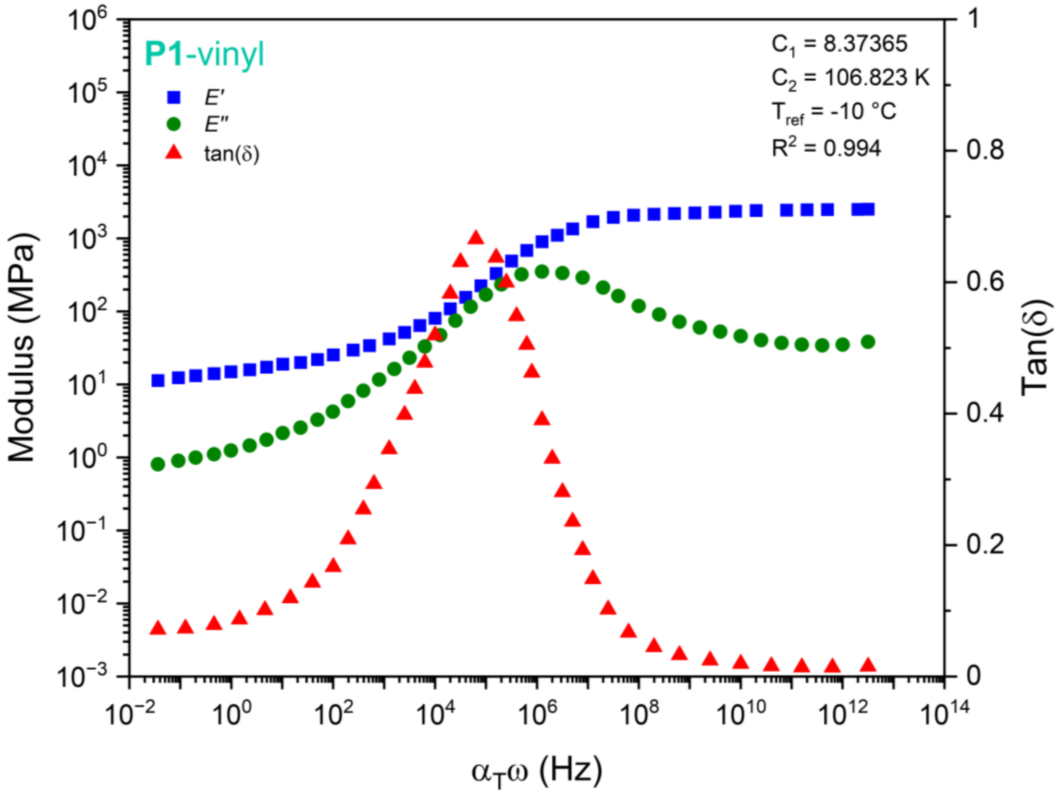

Figure S44: Time-temperature superposition master curve for **P1**-vinyl.

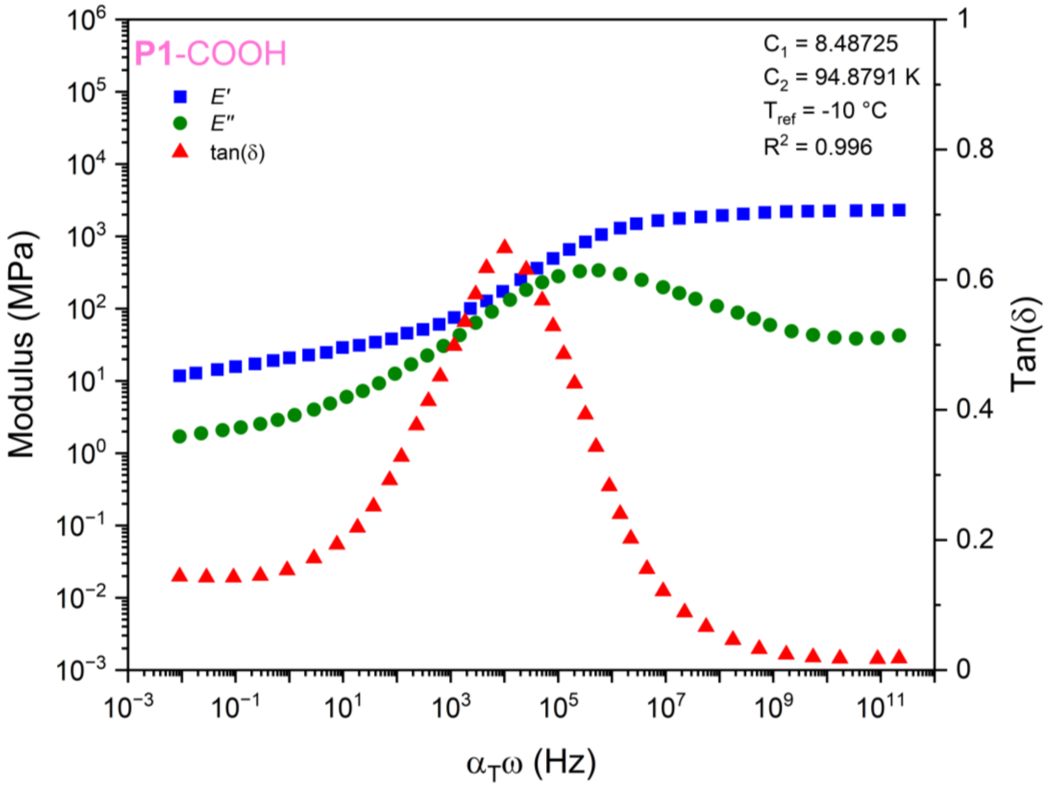

Figure S45: Time-temperature superposition master curve for **P1**-COOH.

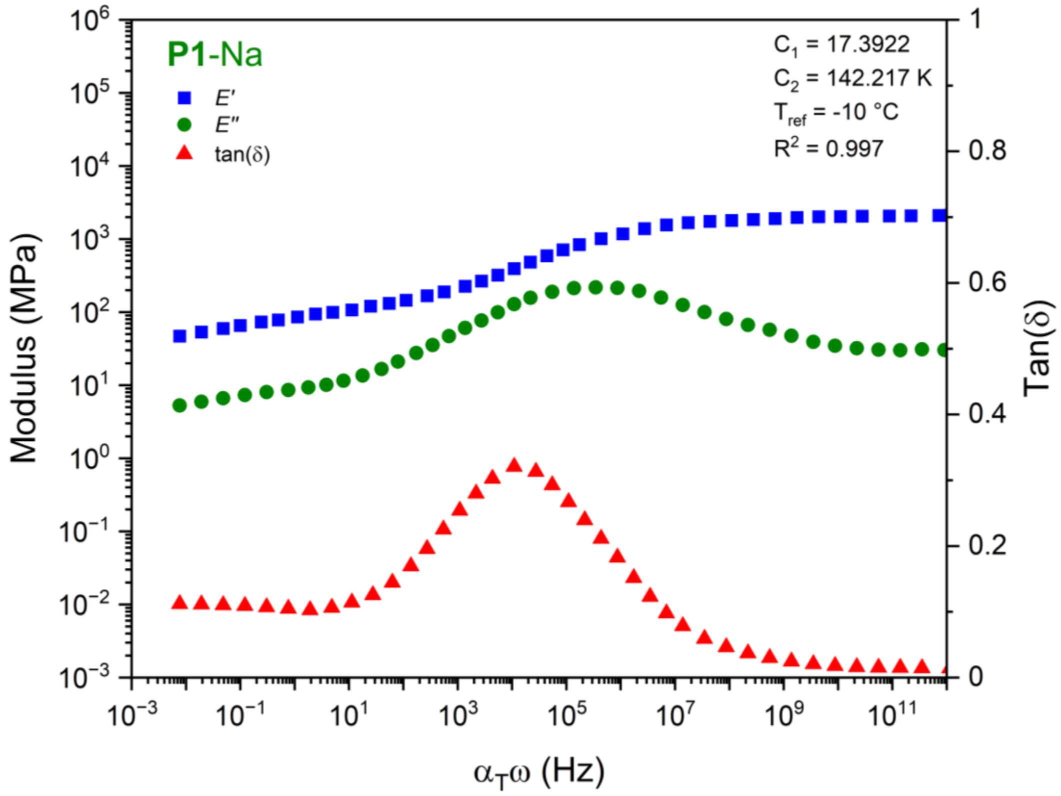

Figure S46: Time-temperature superposition master curve for **P1**-Na.

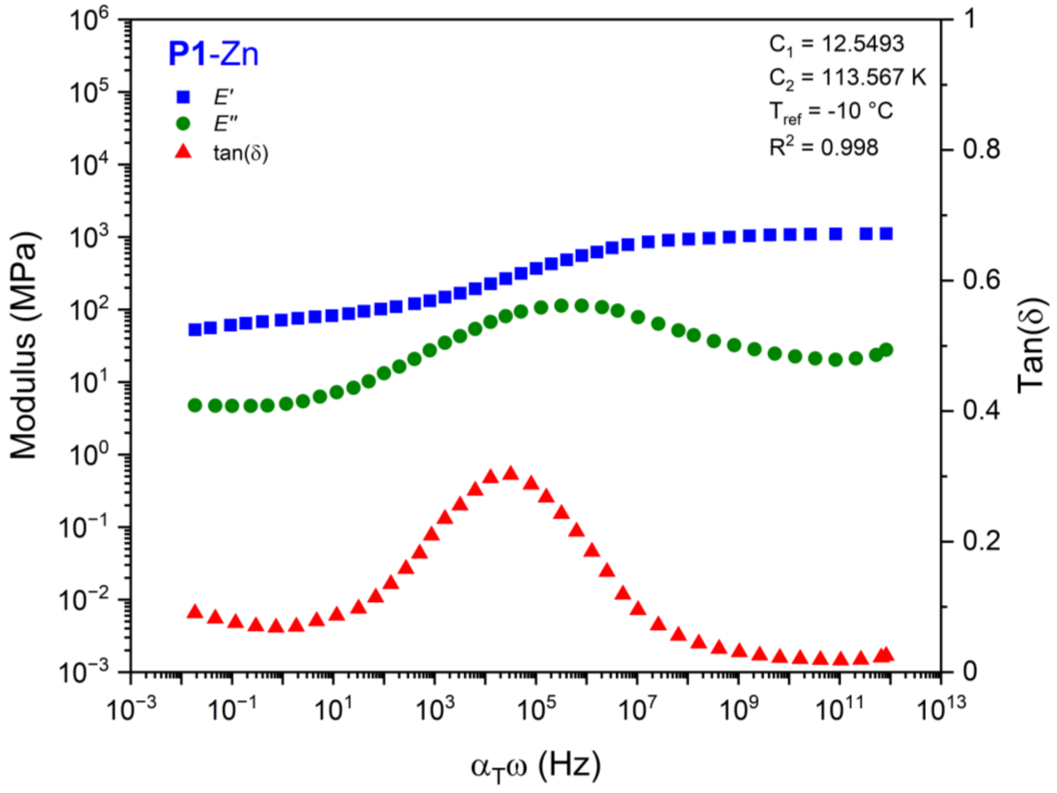

Figure S47: Time-temperature superposition master curve for **P1**-Zn.

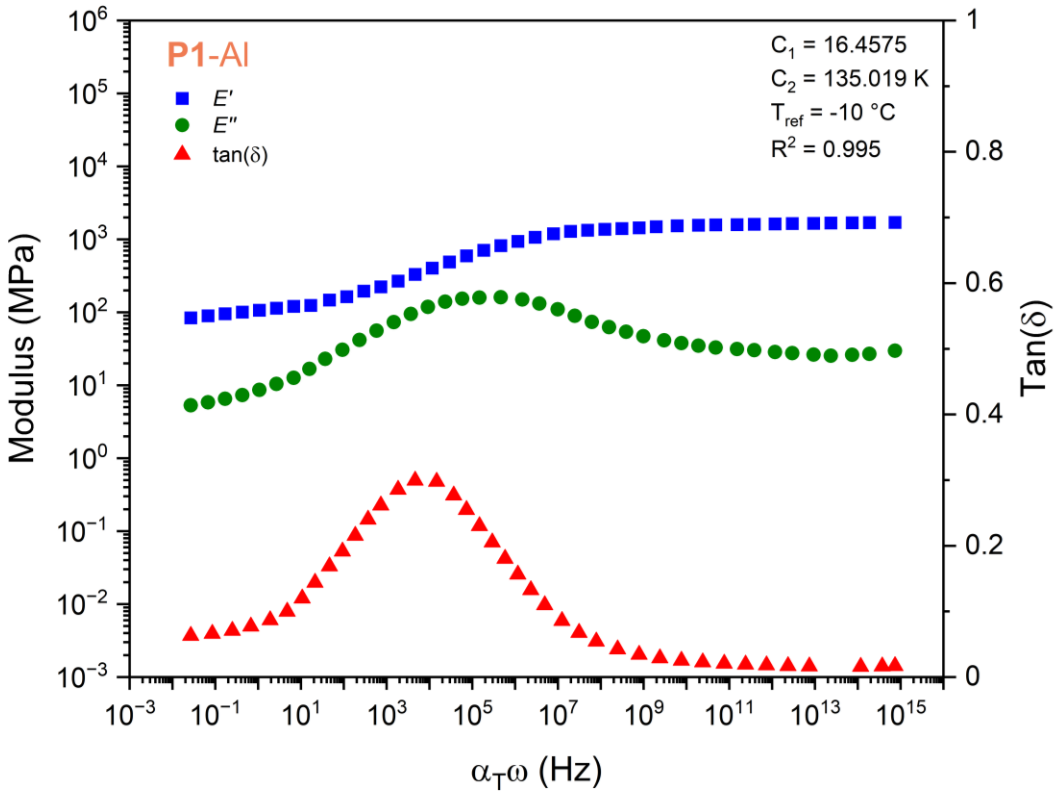

Figure S48: Time-temperature superposition master curve for **P1**-Al.

Table S3: Summary of TTS tan(δ) peaks.

Material	$\tan(\delta)_{Max}$	$\alpha_T\omega_{Peak}$ (Hz)
**P1**-vinyl	0.66	7.96×10^4
**P1**-COOH	0.65	1.01×10^4
**P1**-Na	0.32	1.38×10^4
**P1**-Zn	0.30	2.54×10^4
**P1**-Al	0.30	9.27×10^3

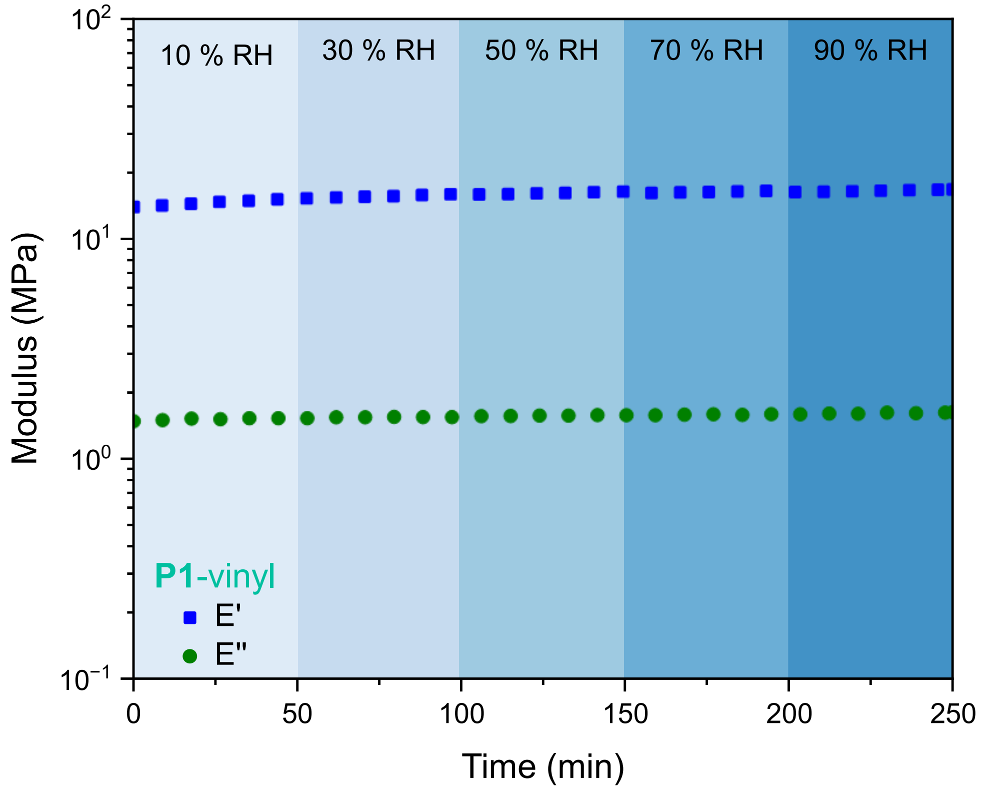

Figure S49: DMA relative humidity (RH) time sweeps for **P1**-vinyl.

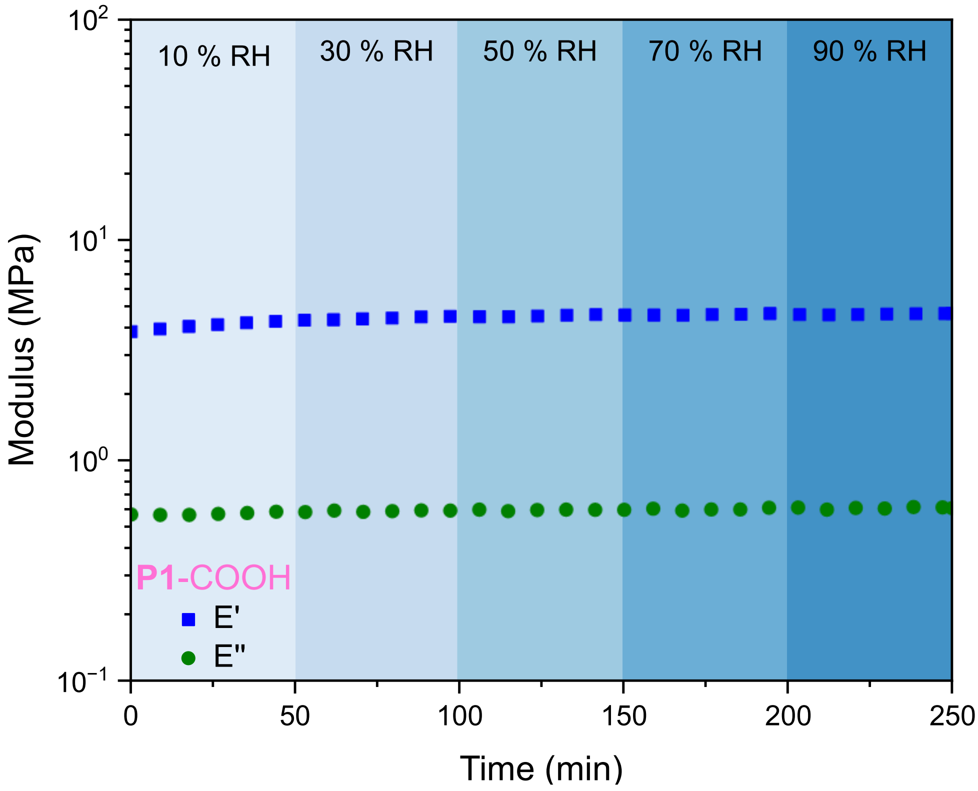

Figure S50: DMA relative humidity (RH) time sweeps for **P1**-COOH.

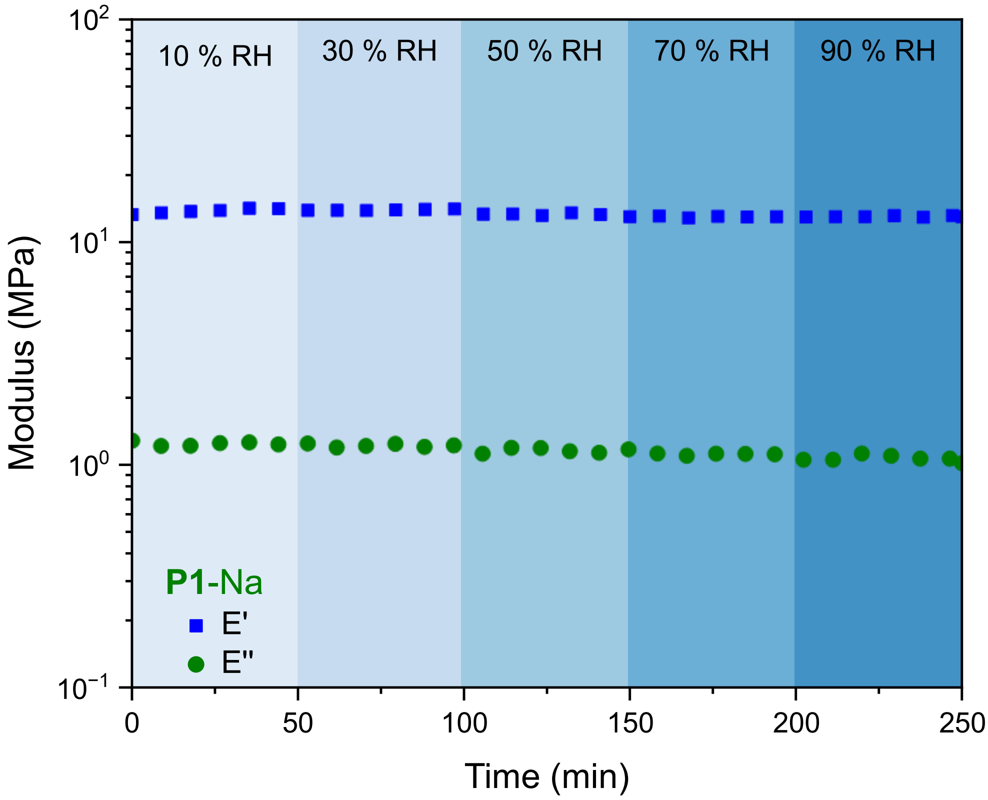

Figure S51: DMA relative humidity (RH) time sweeps for **P1**-Na.

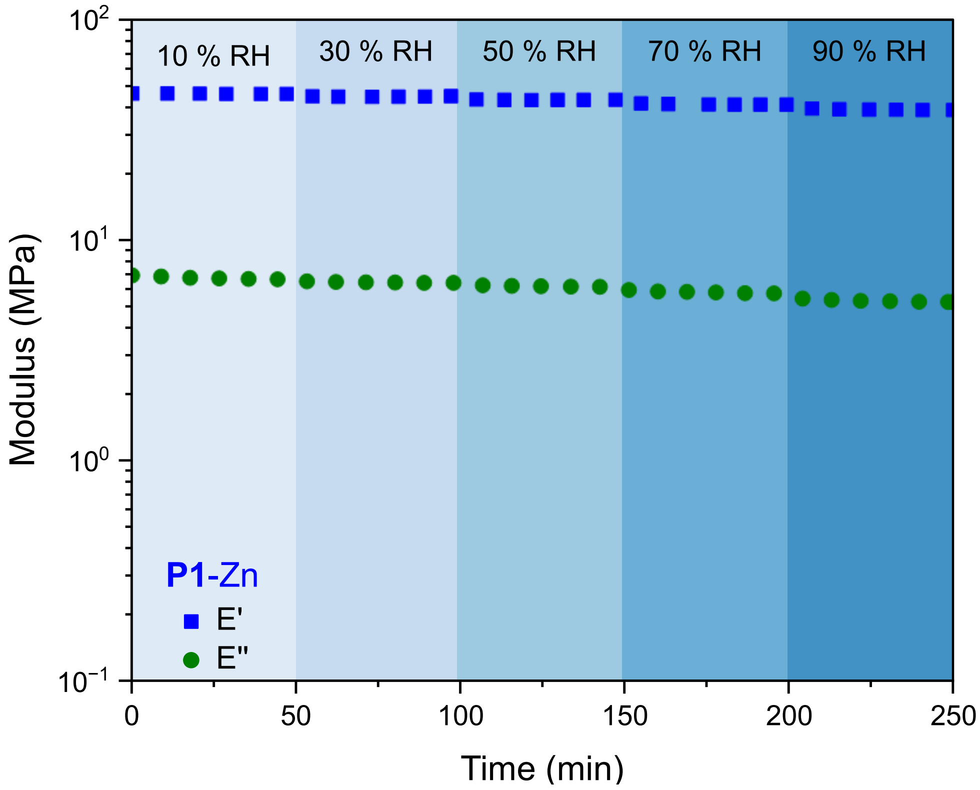

Figure S52: DMA relative humidity (RH) time sweeps for **P1**-Zn.

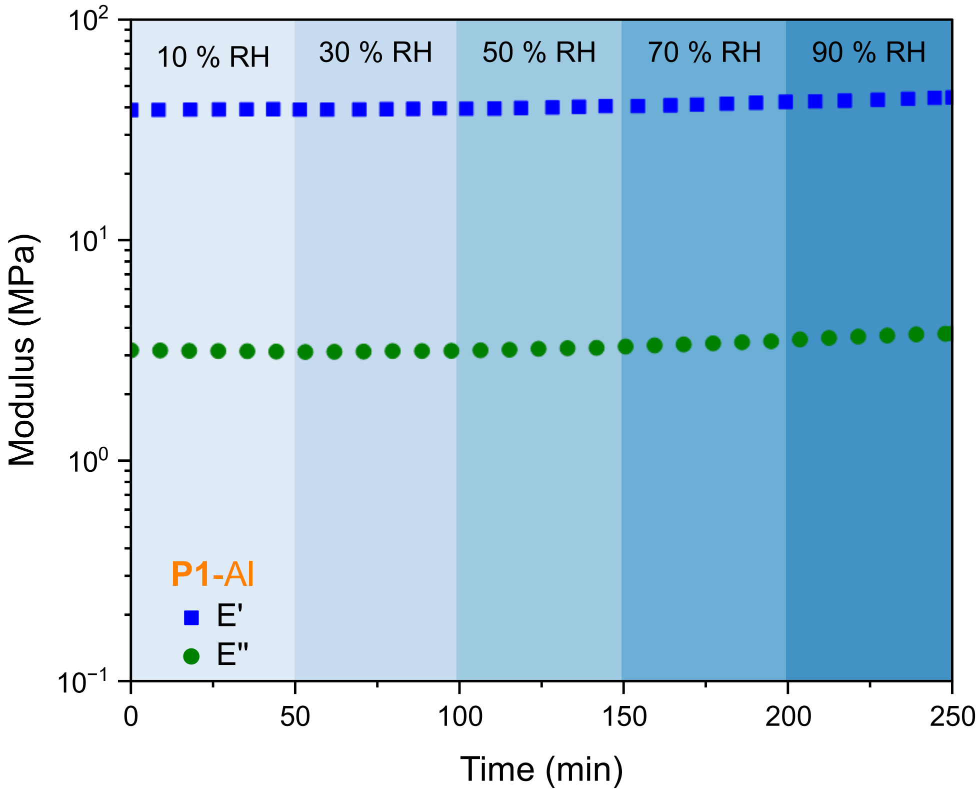

Figure S53: DMA relative humidity (RH) time sweeps for **P1**-Al.

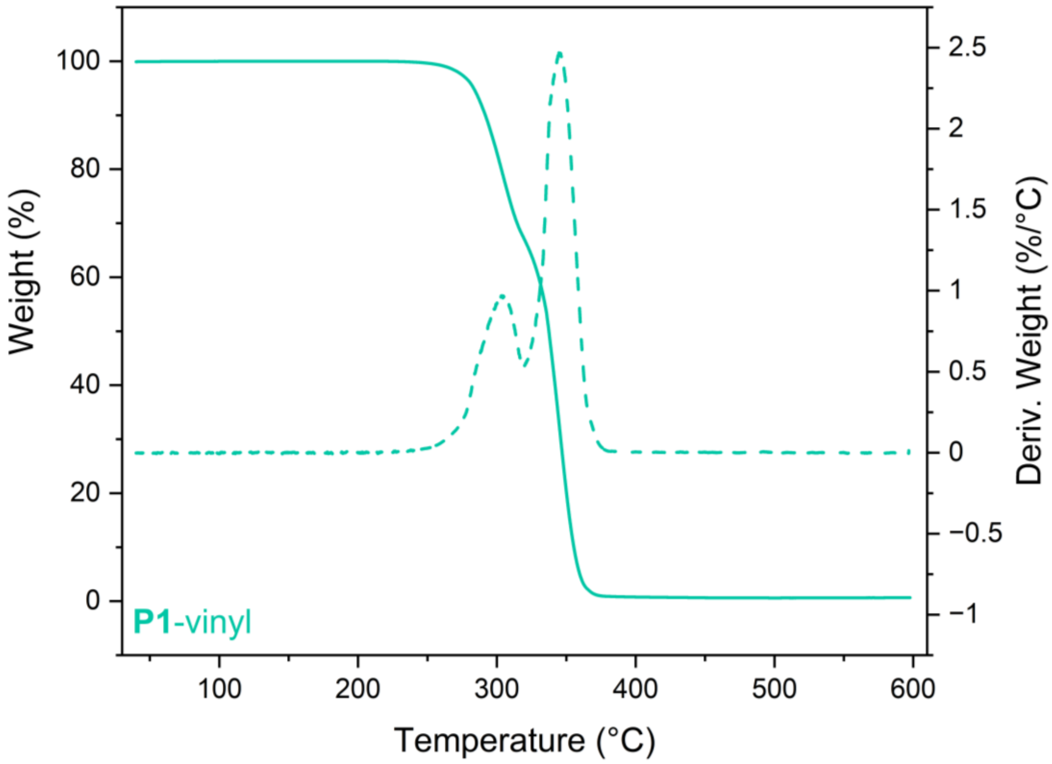

Figure S54: TGA profile for **P1**-vinyl.

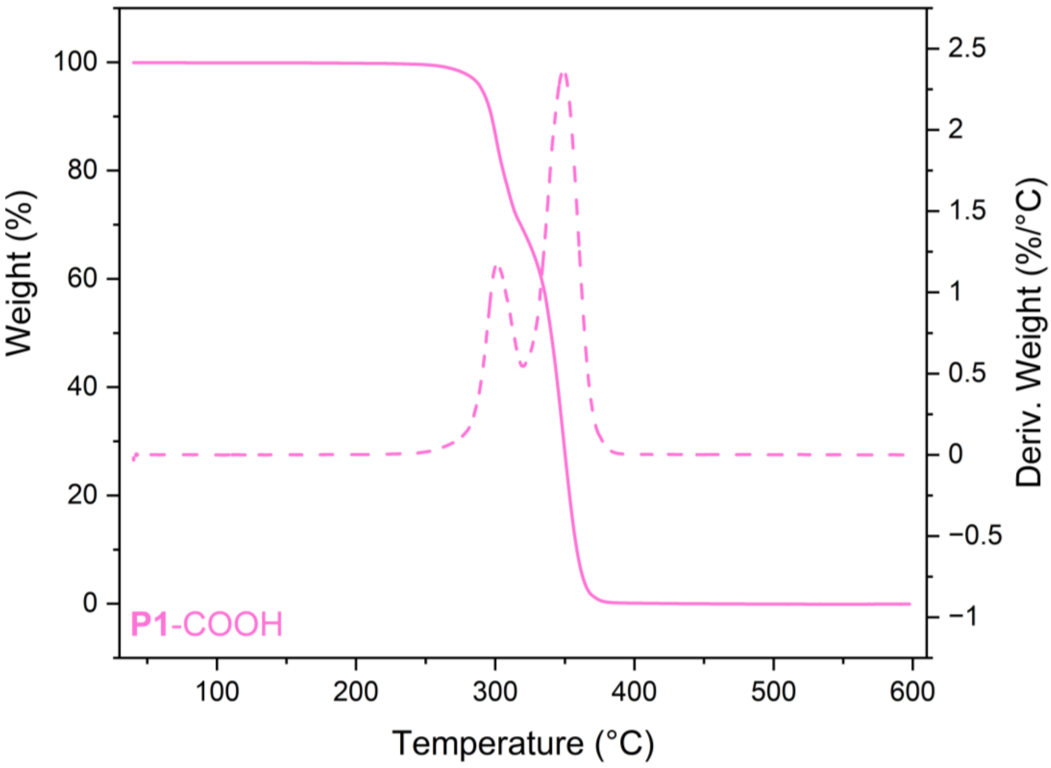

Figure S55: TGA profile for **P1**-COOH.

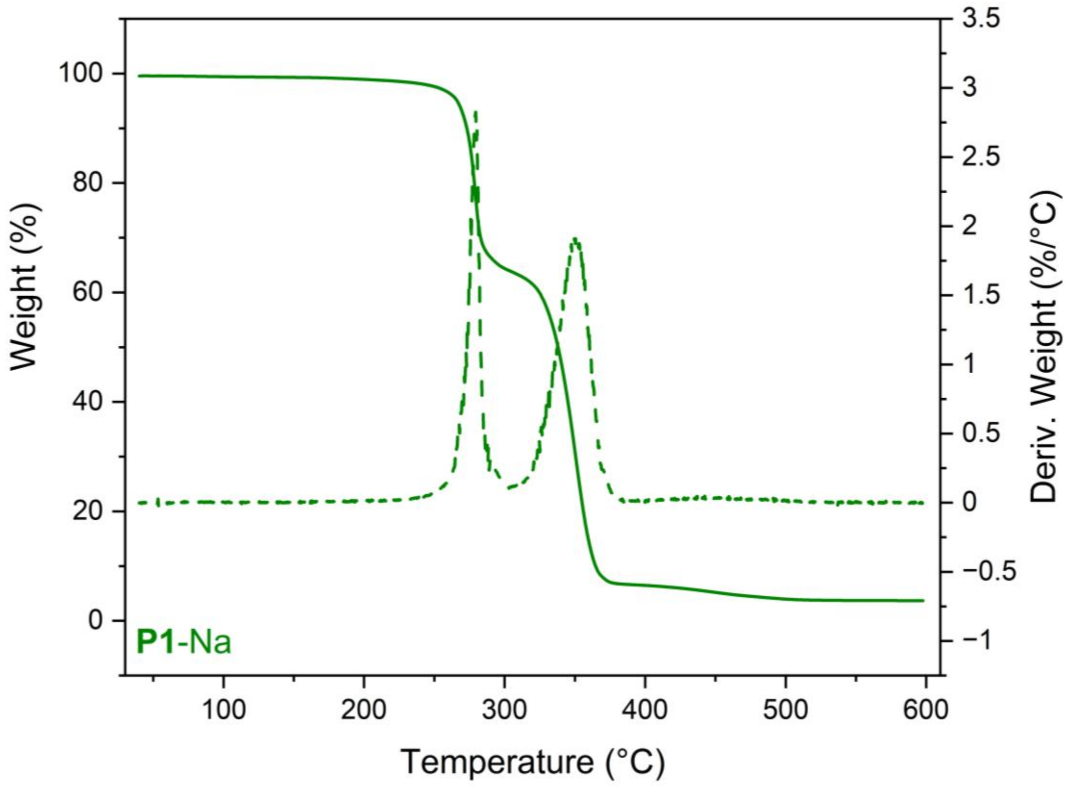

Figure S56: TGA profile for **P1**-Na.

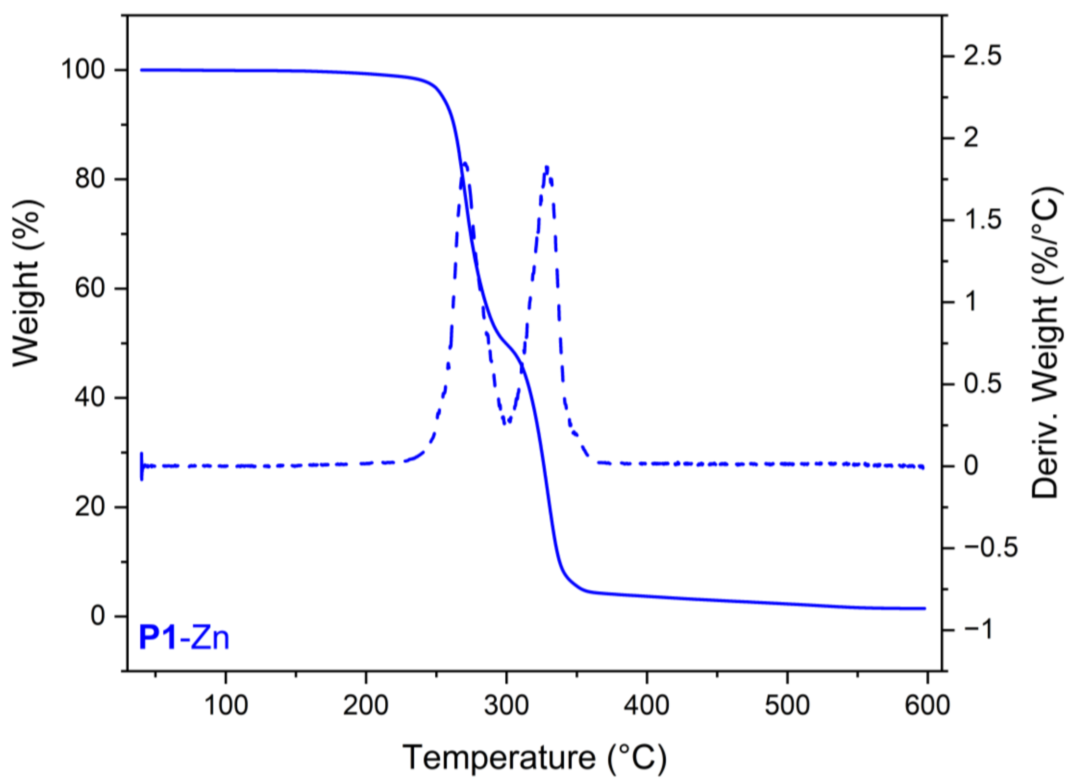

Figure S57: TGA profile for **P1**-Zn.

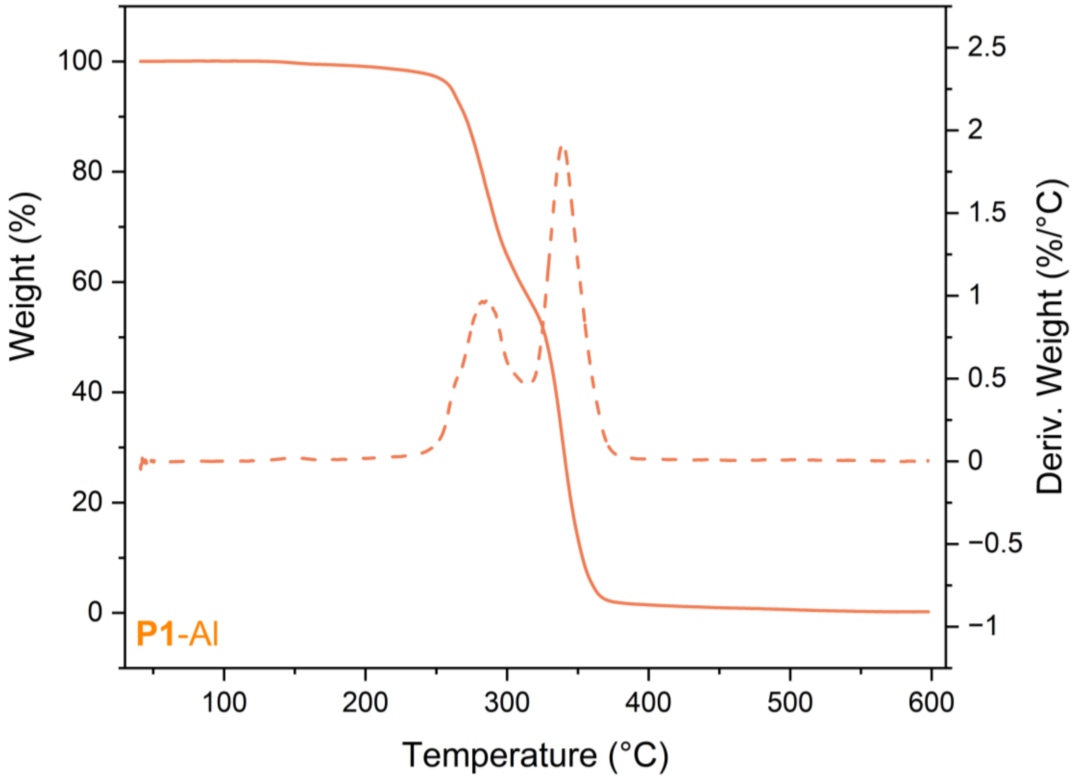

Figure S58: TGA profile for **P1**-Al.

Table S4: Temperature at 5% mass loss during a 10 ˚C/min heating ramp.

Polymer	$T_{d,5\%}$ (˚C)
P1-vinyl	283
P1-COOH	290
P1-Na	266
P1-Zn	255
P1-Al	261

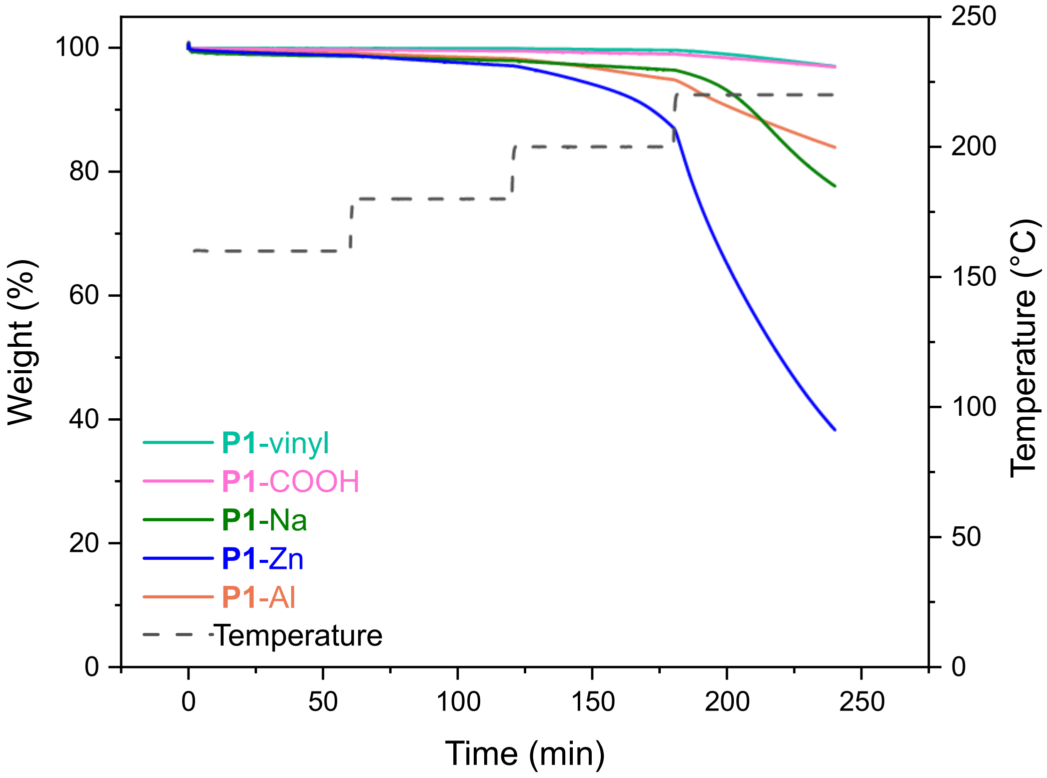

Figure S59: Self-depolymerization screening isothermal TGA experiments.

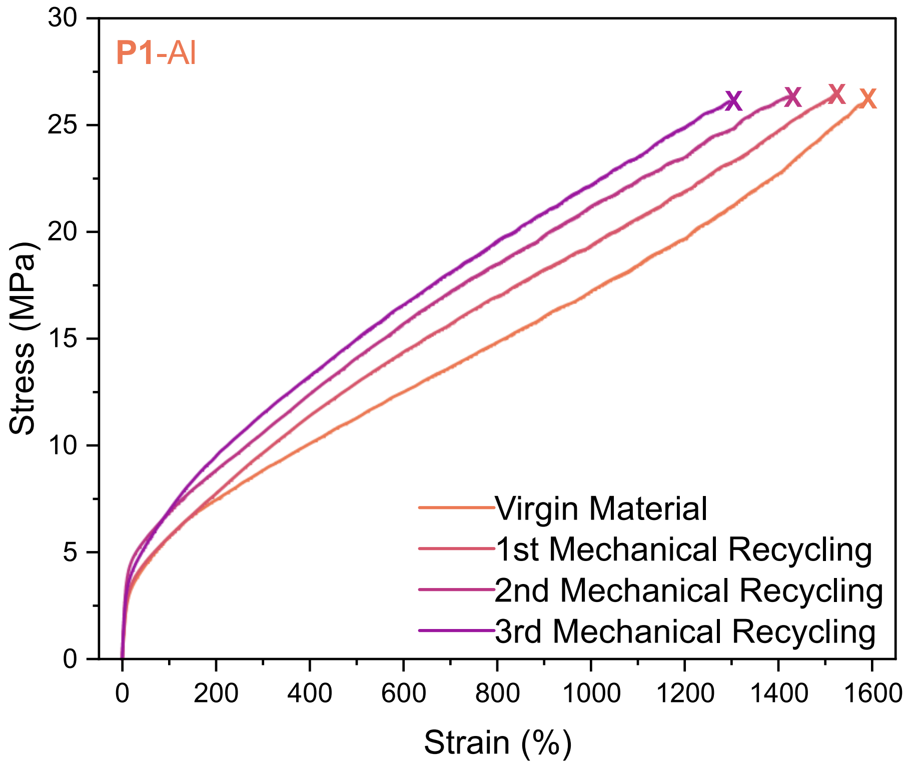

Figure S60: Representative stress-strain curves (10 mm min^{-1} extension rate) for virgin **P1-**Al and after each of three thermal reprocessing (compression moulding) cycles.

Table S5: Summary of tensile mechanical properties after mechanical recycling.

	$^{a}E_{y}$ (MPa)	$^{b}\varepsilon_{b}$ (%)	$^{c}\sigma$ (MPa)	$^{d}U_{T}$ (MJ m^{-3})
Virgin Material	26.2 ± 1.4	1555 ± 74	28.5 ± 5.4	241.0 ± 18.4
1st Mechanical Recycling	26.4 ± 0.8	1466 ± 82	37.3 ± 4.0	247.0 ± 10.8
2nd Mechanical Recycling	25.6 ± 0.8	1371 ± 73	42.2 ± 4.5	226.7 ± 10.8
3rd Mechanical Recycling	25.8 ± 0.9	1275 ± 74	38.3 ± 5.1	211.1 ± 15.9

Specimens suitable for uniaxial tensile testing were compression moulded (140 ˚C, 1.0 ton m^{-2}, 5 min). Measurements were conducted independently on 5 specimens and values are reported as the mean and standard deviation from those experiments. ^{a}Young's modulus. ^{b}Strain at break. ^{c}Tensile strength. ^{d}Tensile toughness (area under the stress-strain curve).

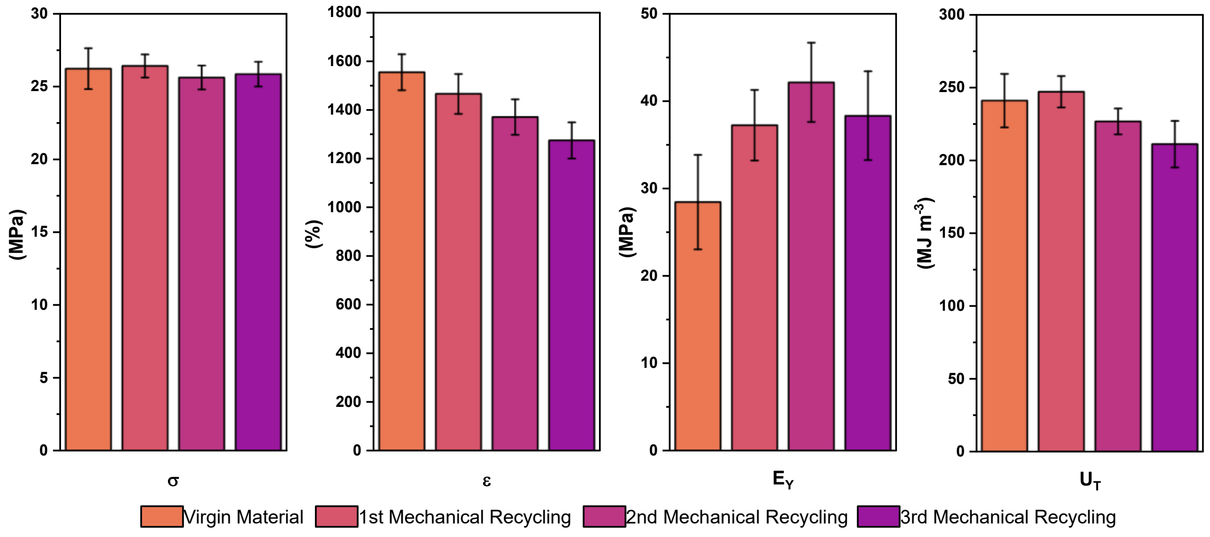

Figure S61: Summary of tensile mechanical properties after mechanical recycling.

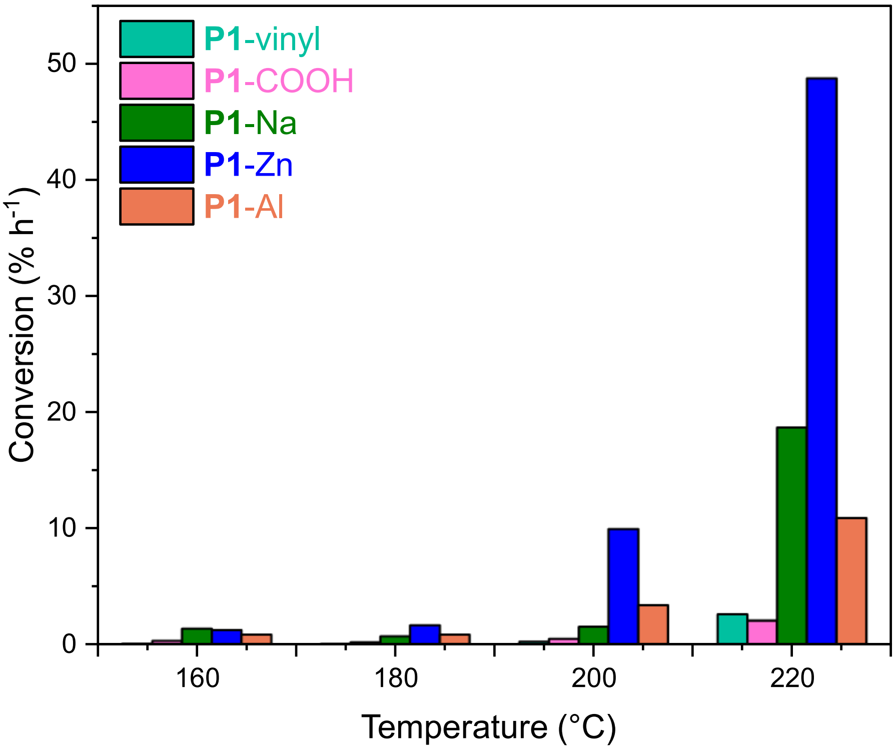

Figure S62: Conversion during self-depolymerization screening.

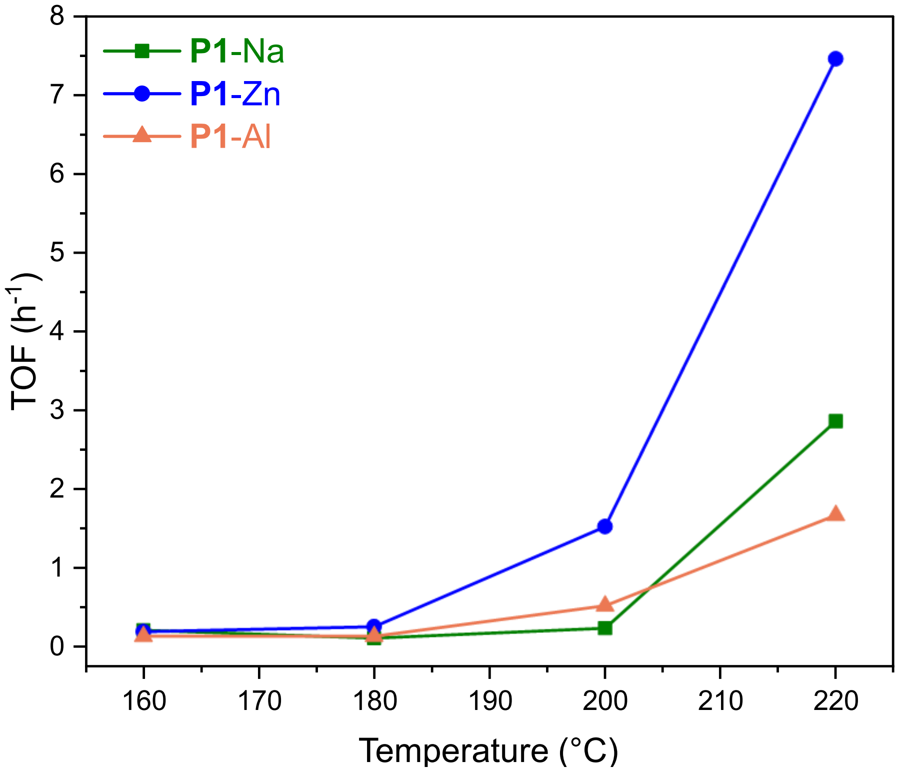

Figure S63: Rate of depolymerization during self-depolymerization screening.

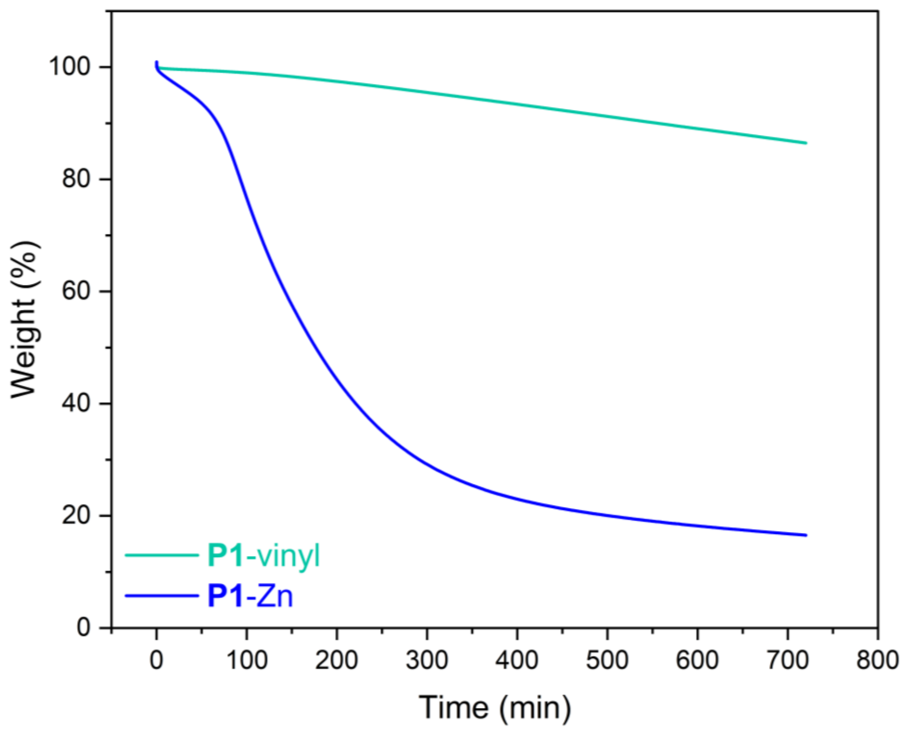

Figure S64: Isothermal TGA experiment (200 ˚C, 12 h).

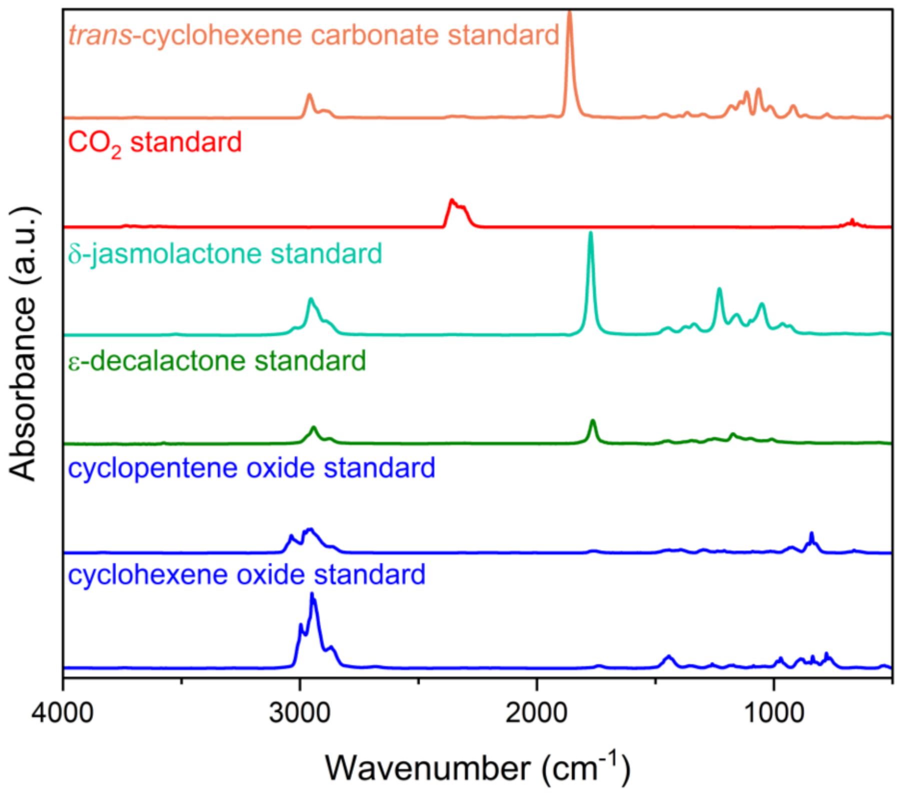

Figure S65: FTIR-TGA standards for expected monomers.

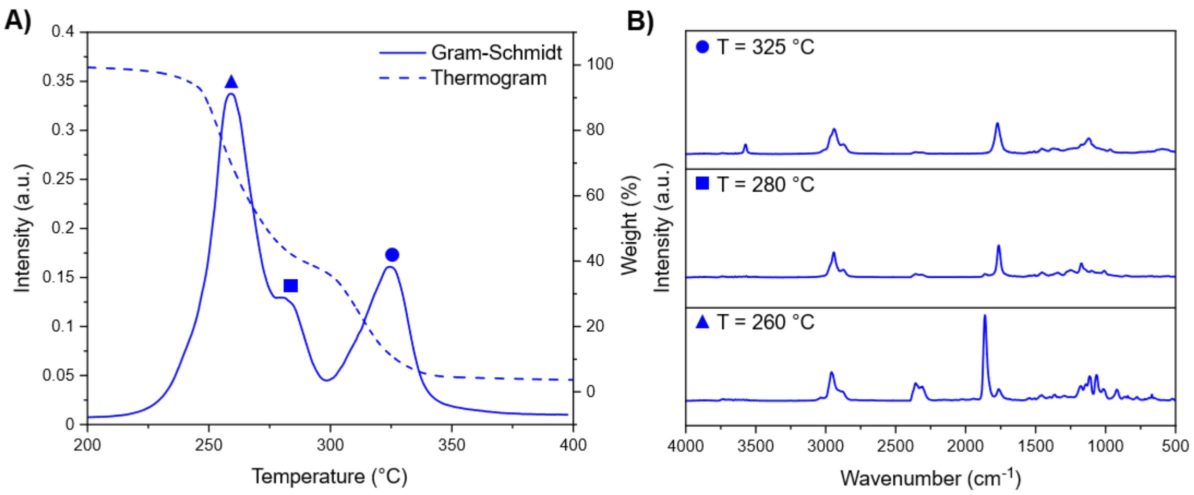

Figure S66: a) FTIR-TGA thermogram with associated Gram-Schmidt profile for **P1**-Zn. b) FTIR spectrum of exhaust gas at 260, 280, and 325 ˚C.

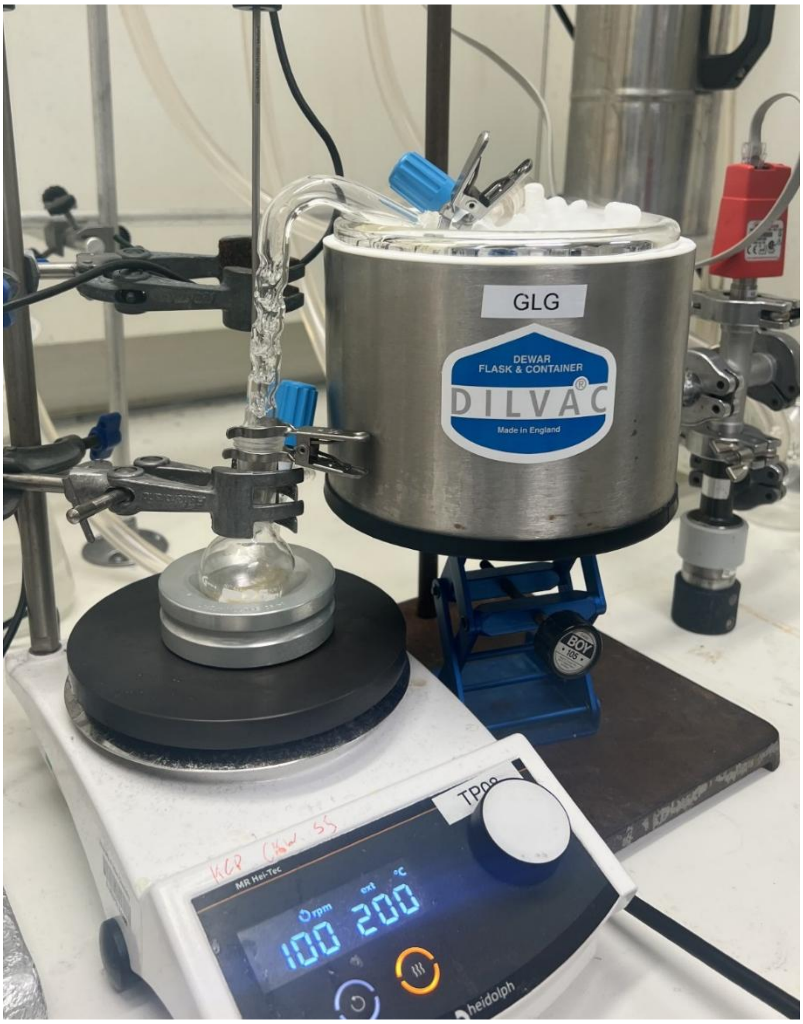

Figure S67: Digital photograph of 0.76 g scale self-depolymerization of **P1**-Zn.

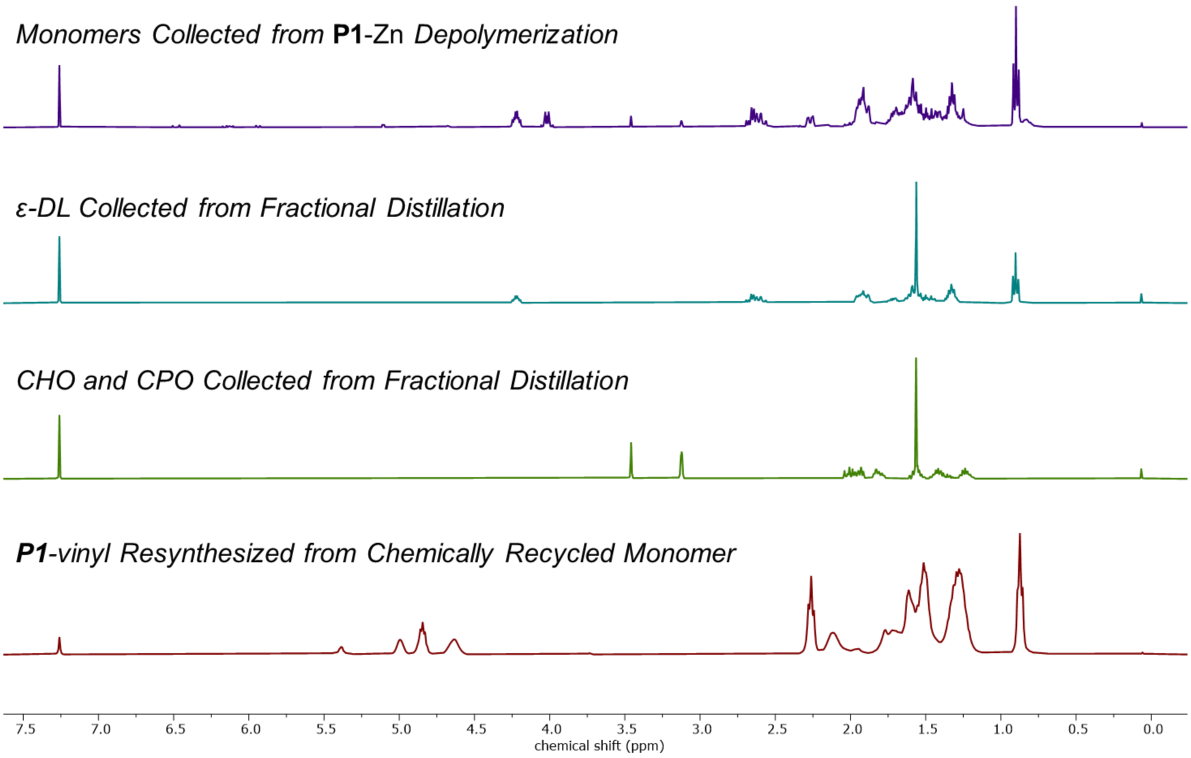

Figure S68: 1H NMR (400 MHz, CDCl3) spectrum of mixture of the monomers after P1-Zn recycling, CHO/CPO and ε-DL separated by fractional distillation (110 ˚C and N_2,120 ˚C and 1.9 x 10-1 mbar atmosphere, respectively), and **P1**-Zn resynthesized from the chemically recycled monomers.

Table S6: Summary of chemical recycling results

	Theoretical Maximum (g)	Recovered After Depolymerization (g)	Yield (%)	Recovered After Fractional Distillation (g)
ε-DL	0.517	0.392	76	0.36
CHO	0.014	0.011	79	0.03
CPO	0.062	0.022	35	
trans-CHC	0.123	0.121	98	-

The theoretical maximum amounts of CHO and *trans*-CHC sum to the mols of PCHC in the block polymer. CHO/*trans*-CHC selectivity determined by ^{1}H NMR.

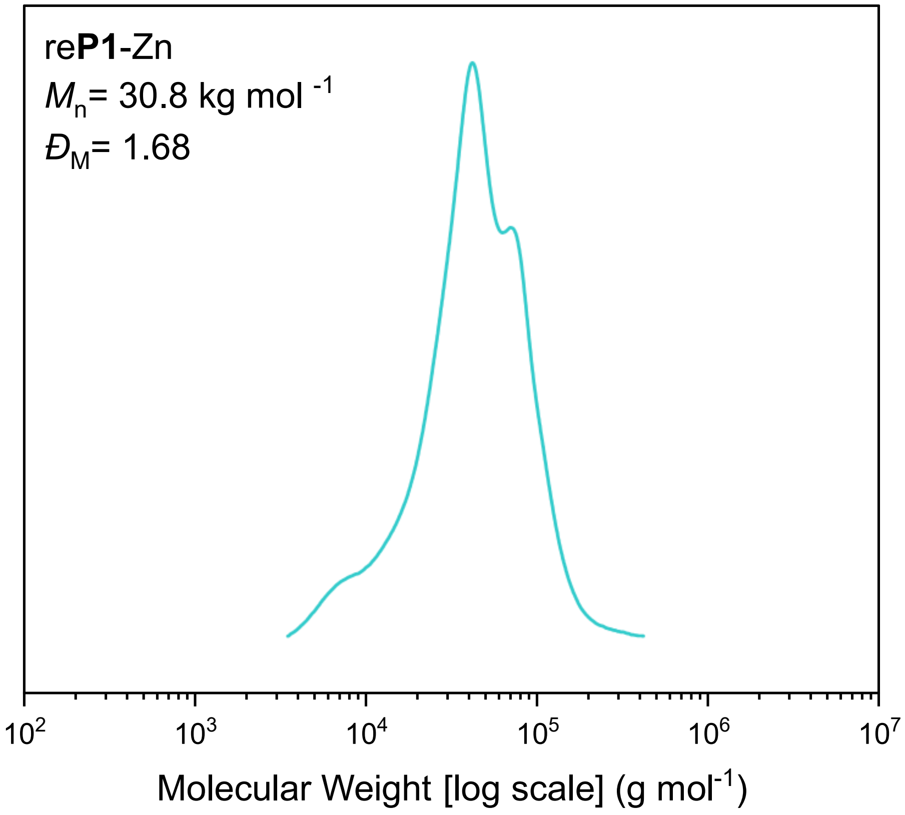

Figure S69: SEC (THF, 1 mL min-1) traces for re**P1**-vinyl. The instrument is calibrated with poly(styrene) standards.

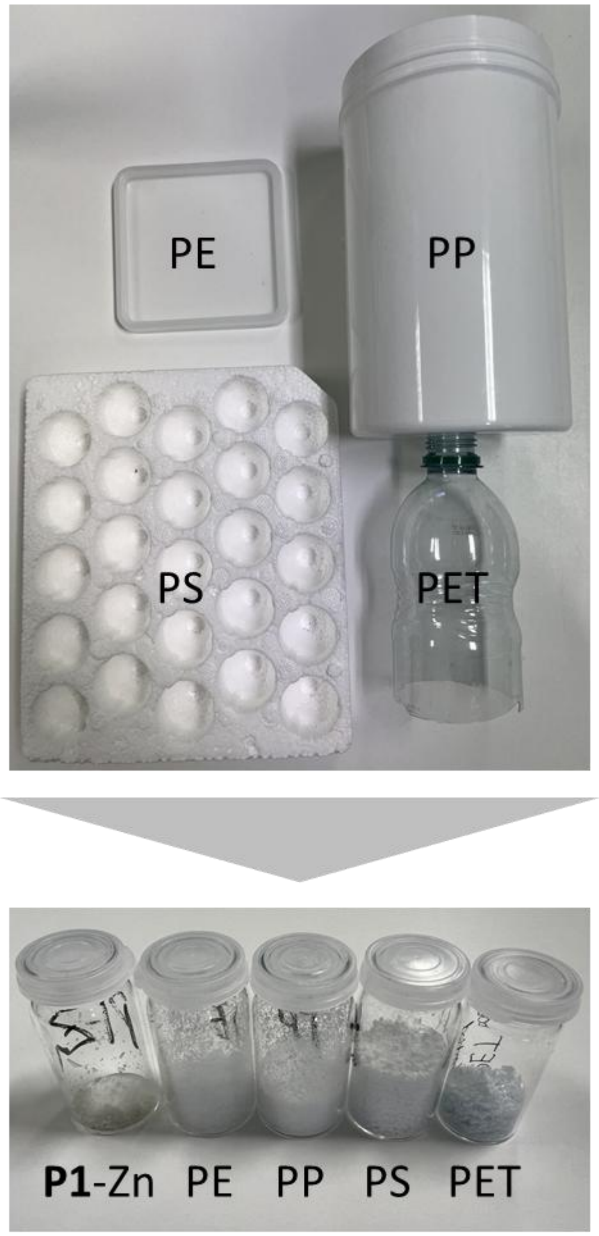

Figure S70: Digital photograph of waste polymers collected from laboratory and office recycling bins (polyethylene (PE), polypropylene (PP), polystyrene (PS), and polyethylene terephthalate (PET). Polymers were all shredded to form physical blends.

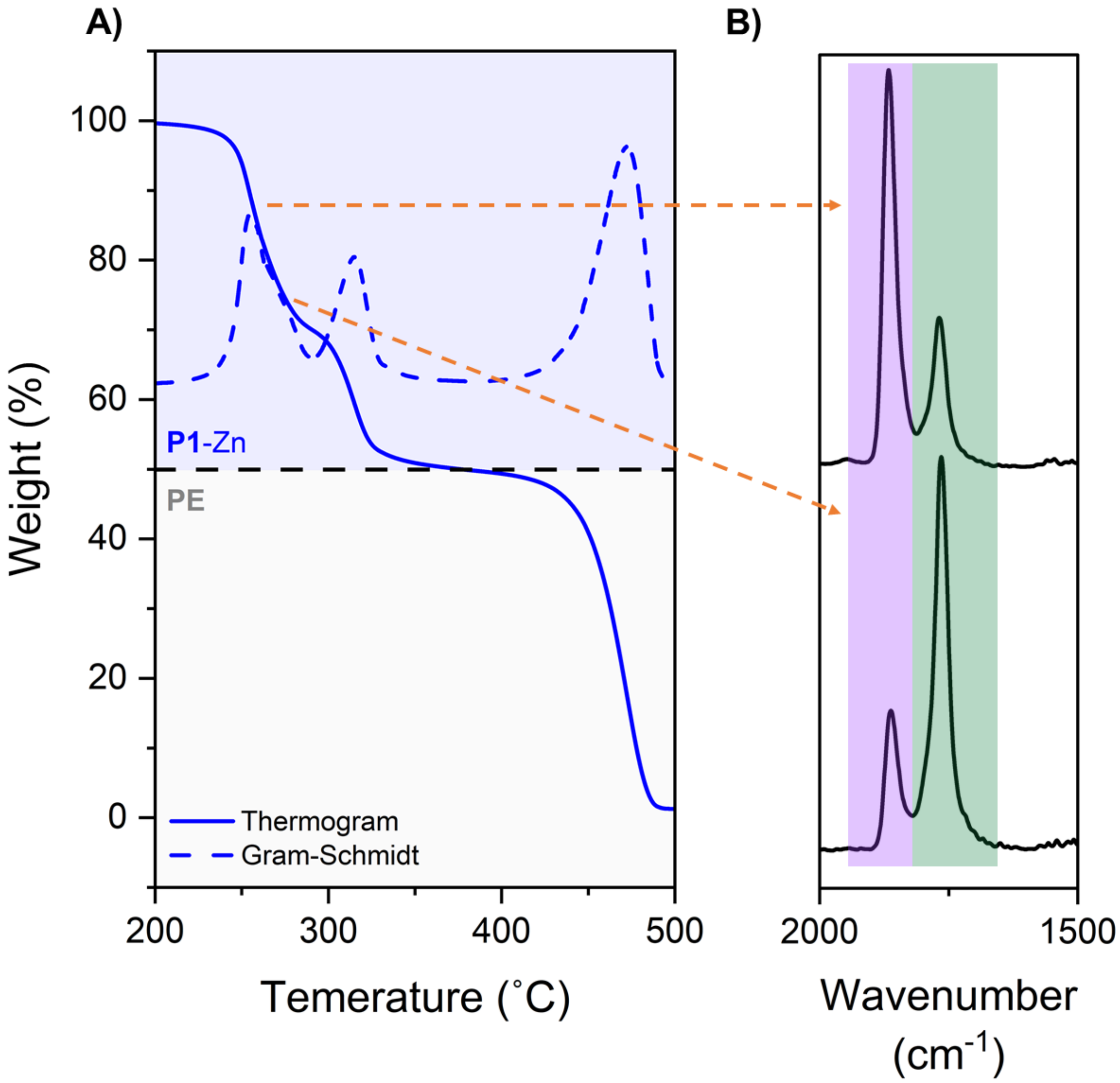

Figure S71: A) TGA thermograms (5 ˚C min^{-1}) for **P1**-Zn and polyethylene (PE) blend (50:50 wt%). B) TGA-FTIR spectra for **P1**-Zn at 260 and 280 ˚C.

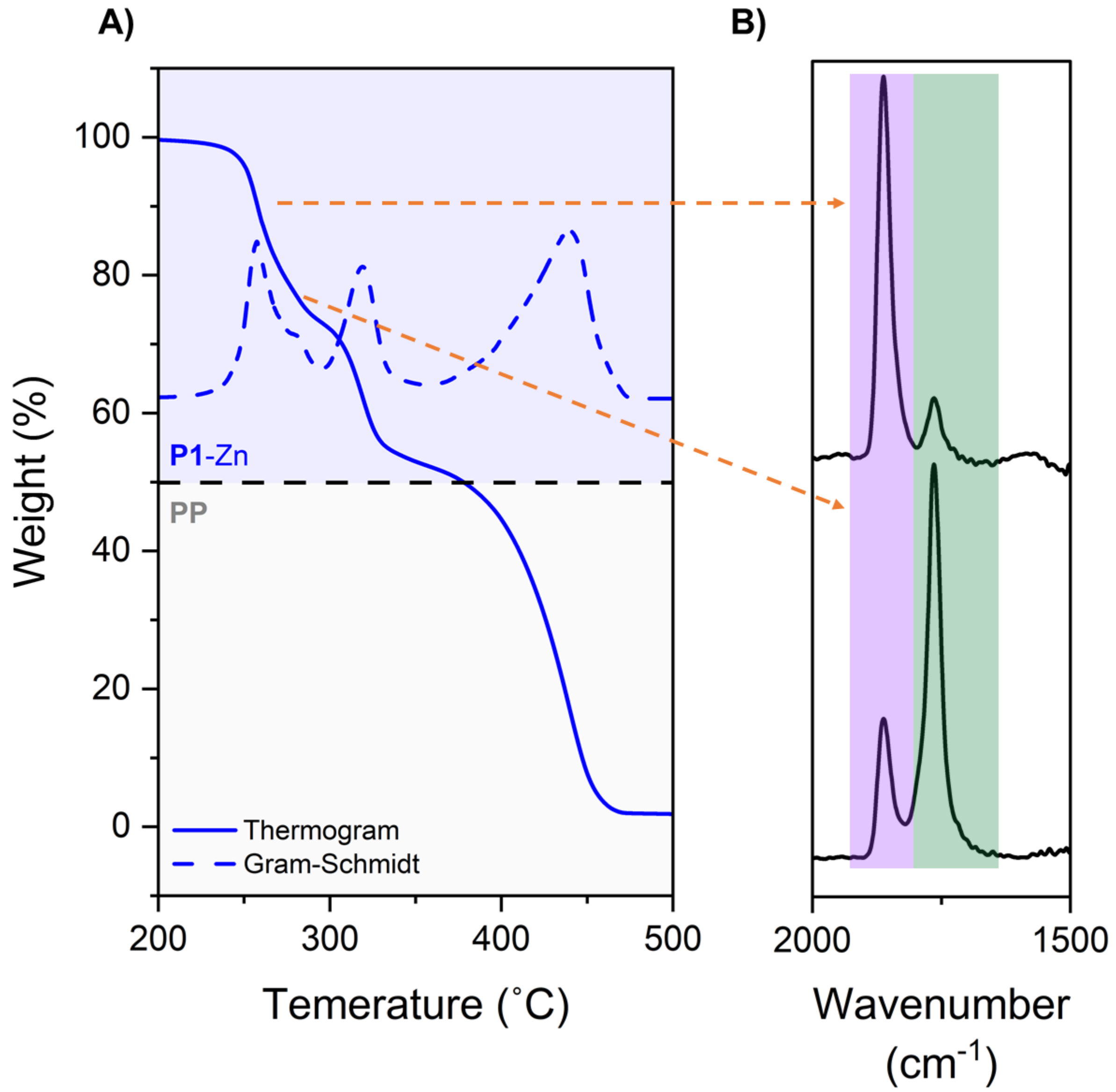

Figure S72: A) TGA thermograms (5 ˚C min^{-1}) for **P1**-Zn and polypropylene (PP) blend (50:50 wt%). B) TGA-FTIR spectra for **P1**-Zn at 260 and 280 ˚C, showing lactone (green) and *trans*-CHC (purple).

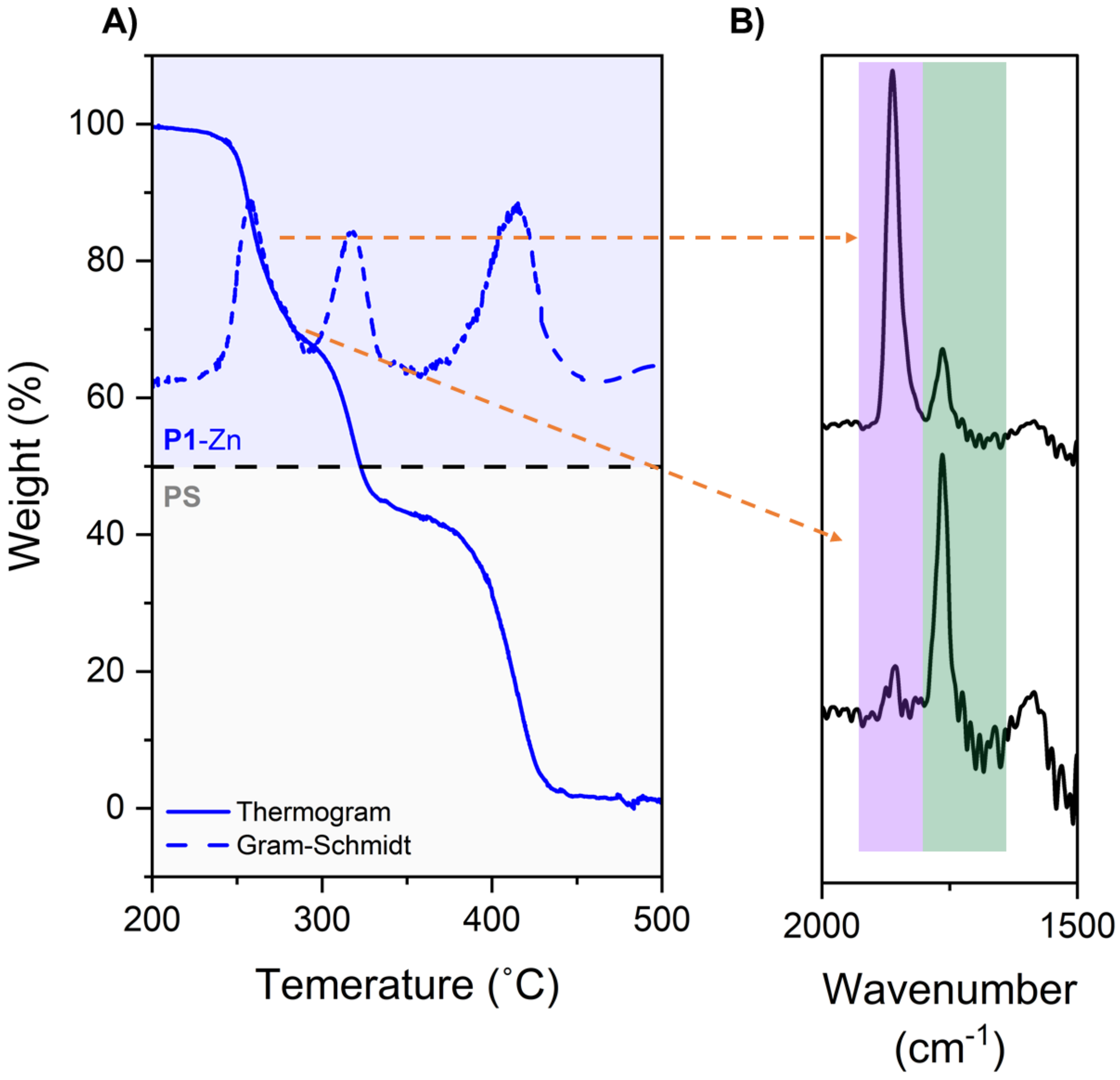

Figure S73: A) TGA thermograms (5 ˚C min^{-1}) for **P1**-Zn and polystyrene (PS) blend (50:50 wt%). B) TGA-FTIR spectra for **P1**-Zn at 260 and 280 ˚C, showing lactone (green) and *trans*-CHC (purple).

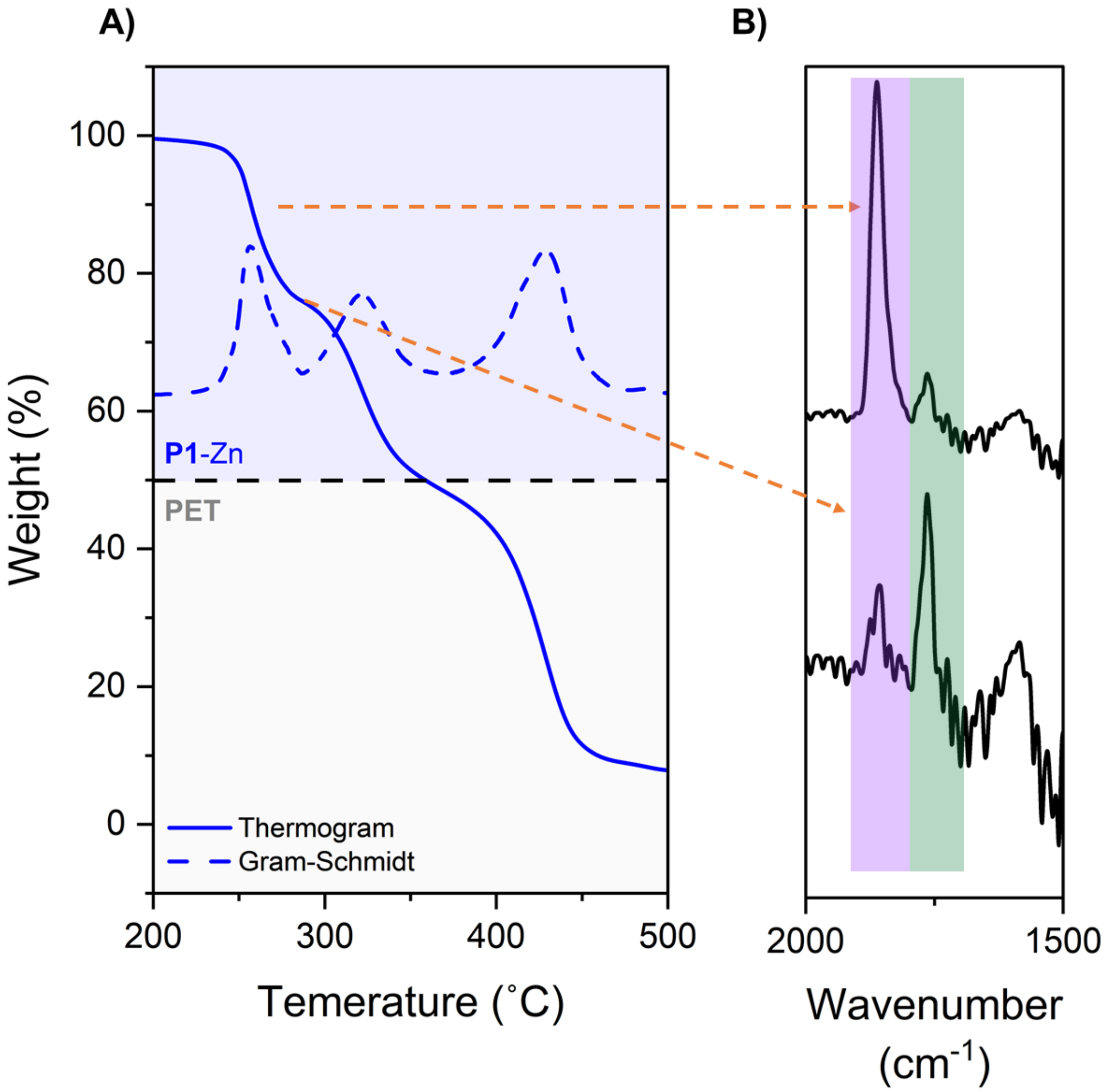

Figure S74: A) TGA thermograms (5 ˚C min^{-1}) for **P1**-Zn and polyethylene terephthalate (PET) blend (50:50 wt%). B) TGA-FTIR spectra for **P1**-Zn at 260 and 280 ˚C, showing lactone (green) and *trans*-CHC (purple).

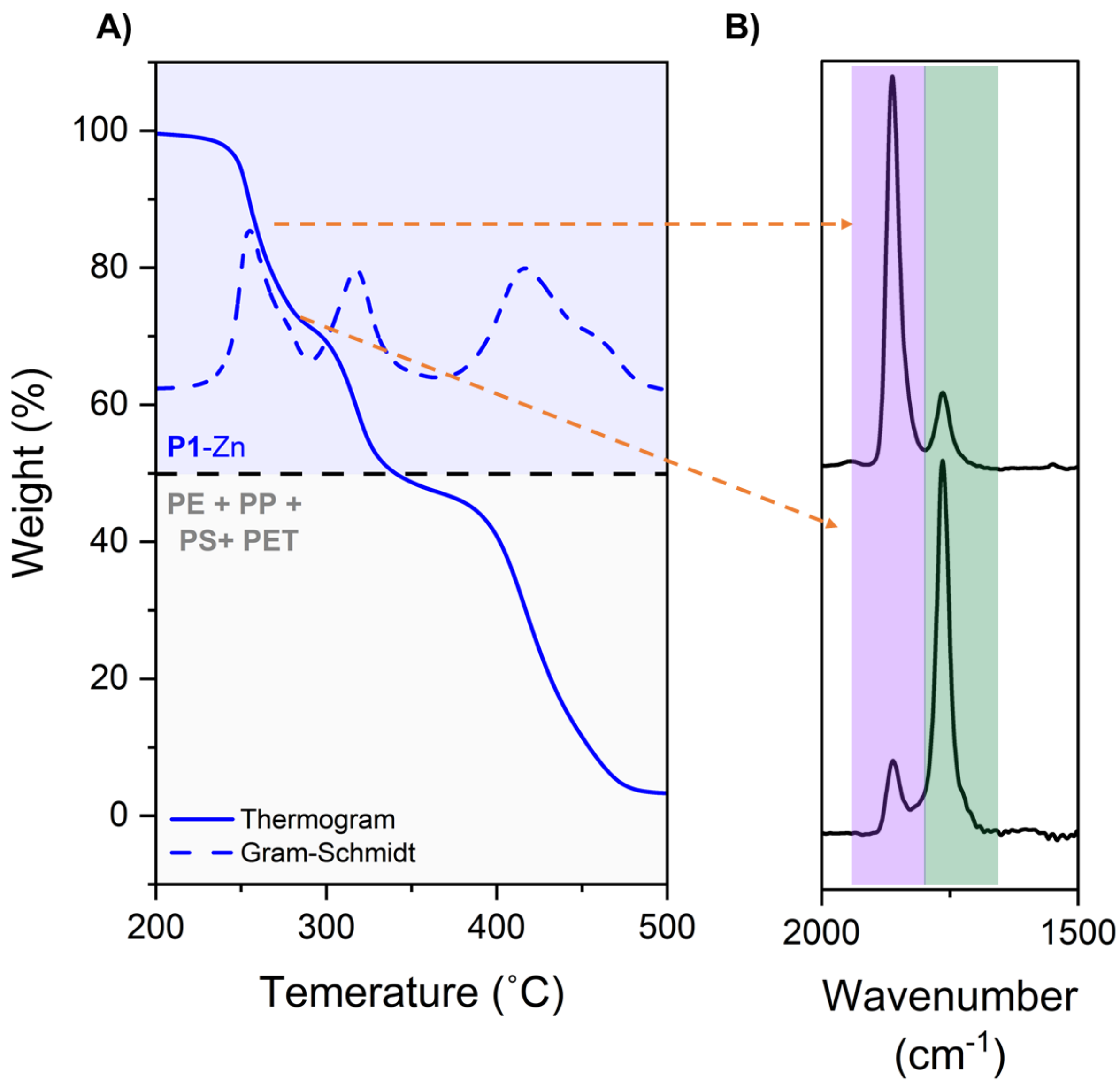

Figure S75: A) TGA thermograms (5 ˚C min^{-1}) for **P1**-Zn and polyethylene, polypropylene (PP), polystyrene (PS), and polyethylene terephthalate (PET) blend (50:12.5:12.5:12.5:12.5 wt%). B) TGA-FTIR spectra for **P1**-Zn at 260 and 280 ˚C, showing lactone (green) and *trans*-CHC (purple).

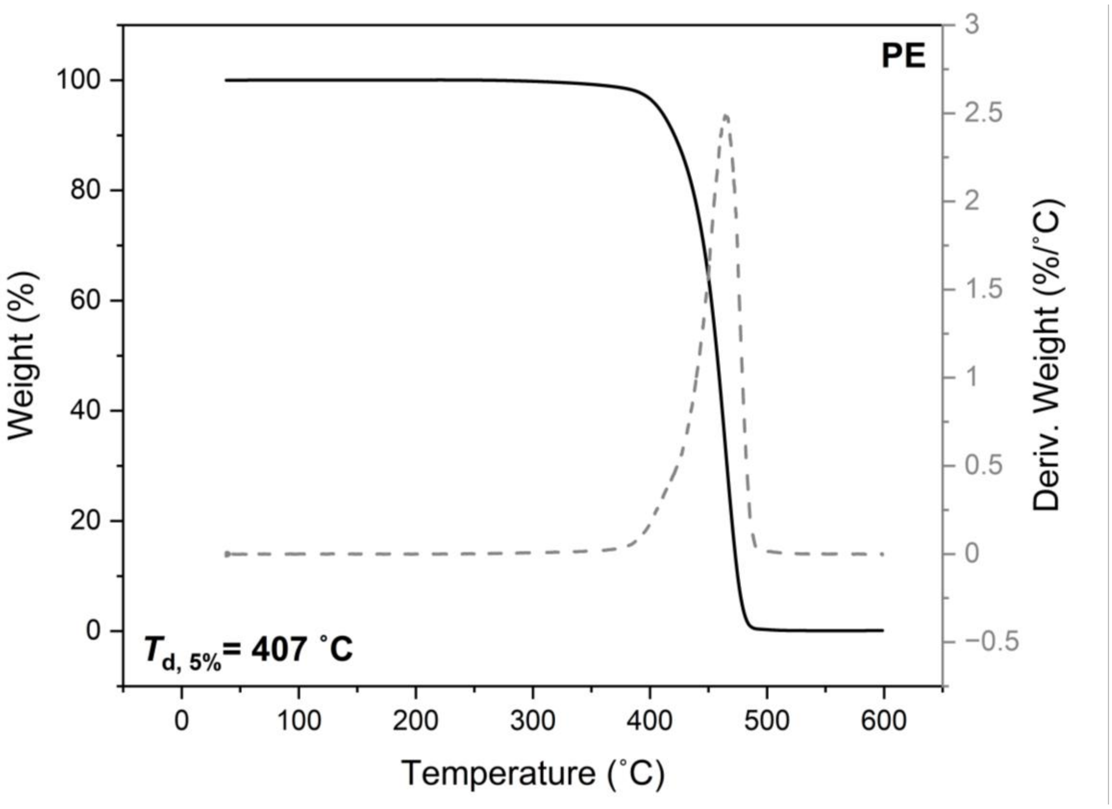

Figure S76: TGA profile for PE.

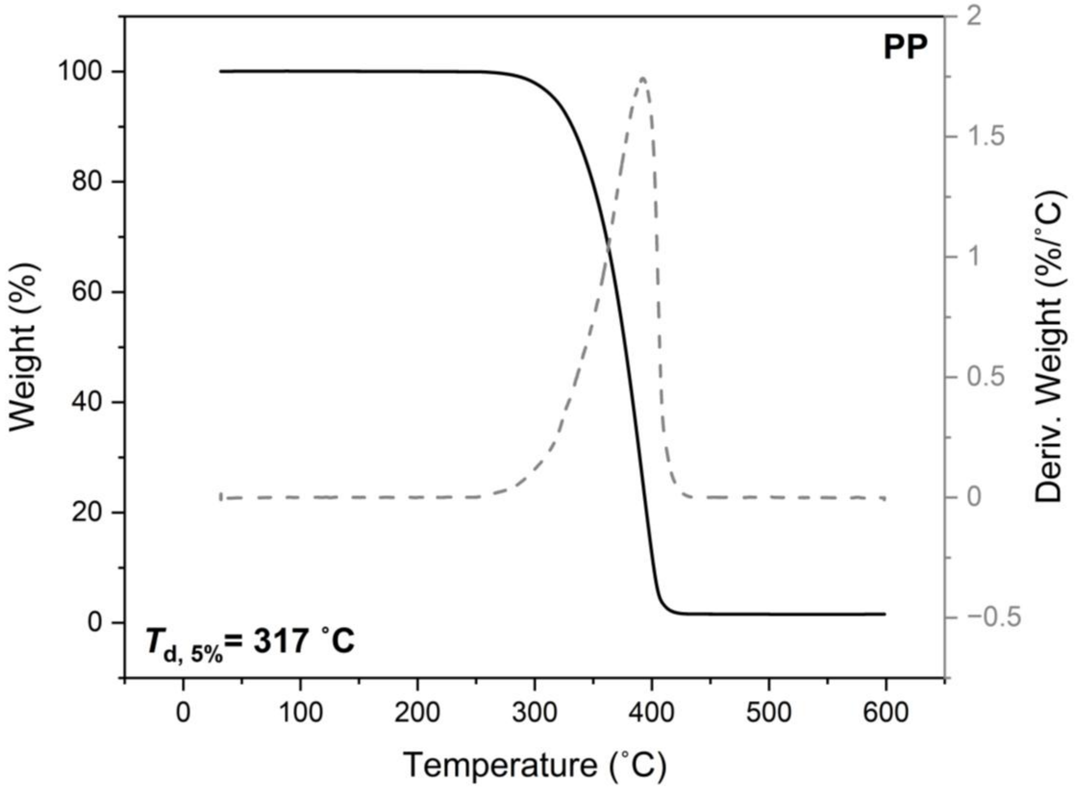

Figure S77: TGA profile for PP.

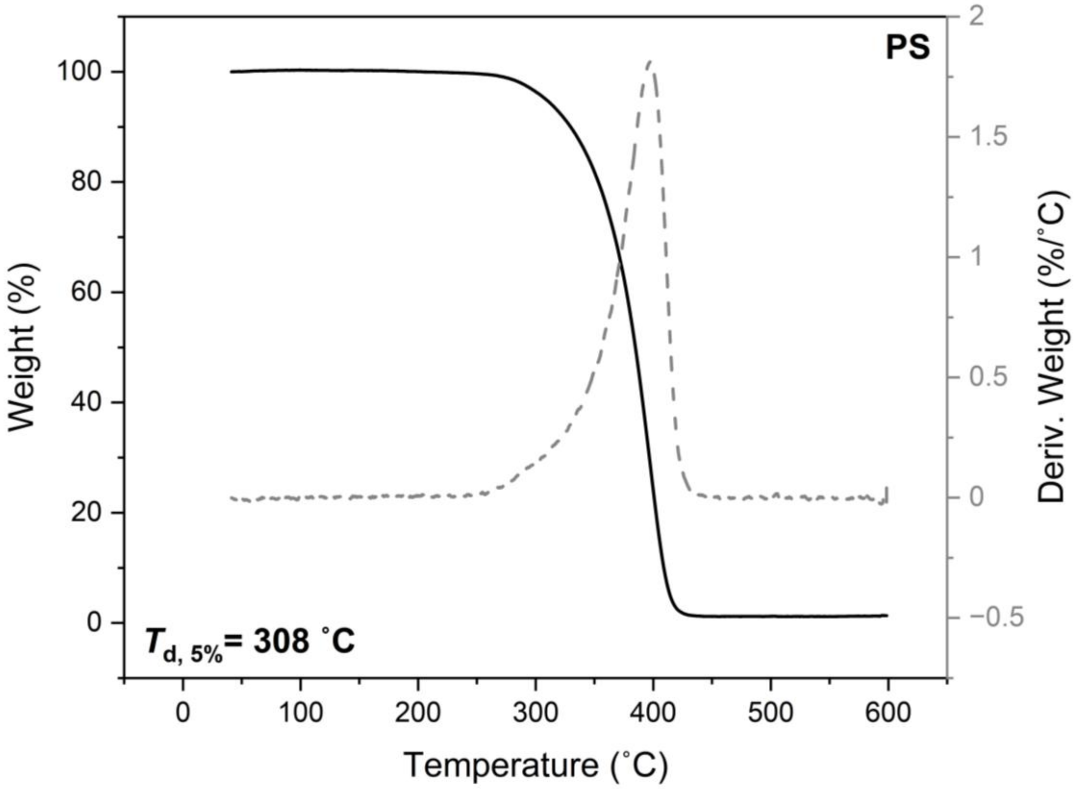

Figure S78: TGA profile for PS.

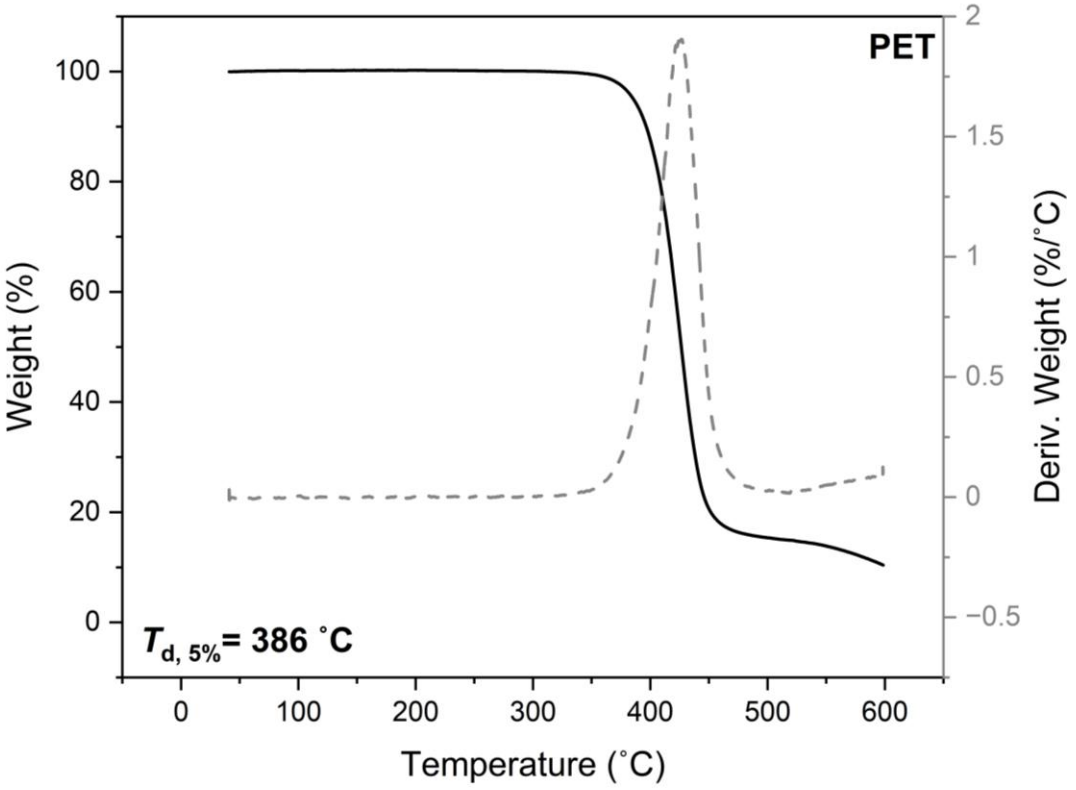

Figure S79: TGA profile for PET.

References

1. A. Spyros, D. S. Argyropoulos, R. H. Marchessault, A study of poly(hydroxyalkanoate)s by quantitative P-31 NMR spectroscopy: Molecular weight and chain cleavage. *Macromolecules* **30**, 327-329 (1997).
2. J. Filik *et al.*, Processing two-dimensional X-ray diffraction and small-angle scattering data in DAWN 2. *J. Appl. Crystallogr.* **50**, 959-966 (2017).
3. B. R. Pauw, A. J. Smith, T. Snow, N. J. Terrill, A. F. Thünemann, The modular small-angle X-ray scattering data correction sequence. *J Appl Crystallogr* **50**, 1800-1811 (2017).
4. G. S. Sulley *et al.*, Switchable Catalysis Improves the Properties of CO2-Derived Polymers: Poly(cyclohexene carbonate- b-ϵ-decalactone- b-cyclohexene carbonate) Adhesives, Elastomers, and Toughened Plastics. *J. Am. Chem. Soc.* **142**, 4367-4378 (2020).
5. N. Kazemi, T. A. Duever, A. Penlidis, Reactivity Ratio Estimation from Cumulative Copolymer Composition Data. *Macromol. React. Eng.* **5**, 385-403 (2011).
